# A seismically induced onshore surge deposit at the KPg boundary, North Dakota

**DOI:** 10.1073/pnas.1817407116

**Published:** 2019-04-01

**Authors:** Robert A. DePalma, Jan Smit, David A. Burnham, Klaudia Kuiper, Phillip L. Manning, Anton Oleinik, Peter Larson, Florentin J. Maurrasse, Johan Vellekoop, Mark A. Richards, Loren Gurche, Walter Alvarez

**Affiliations:** ^a^Department of Geology, University of Kansas, Lawrence, KS 66045;; ^b^Department of Vertebrate Paleontology, The Palm Beach Museum of Natural History, Fort Lauderdale, FL 33306;; ^c^Department of Geosciences, Florida Atlantic University, Boca Raton, FL 33431;; ^d^Department of Geology and Geochemistry, Faculty of Science, Vrije Universiteit Amsterdam, 1081 HV Amsterdam, The Netherlands;; ^e^Biodiversity Institute, University of Kansas, Lawrence, KS 66045;; ^f^School of Earth & Environmental Sciences, University of Manchester, Manchester M13 9PL, United Kingdom;; ^g^Black Hills Institute of Geological Research, Hill City, SD 57745;; ^h^Department of Earth and Environment, Florida International University, Miami, FL 33199;; ^i^Department of Earth and Environmental Sciences, Catholic University of Leuven, 3001 Leuven, Belgium;; ^j^Analytical, Environmental, and Geo- Chemistry, Vrije Universiteit Brussel, B-1050 Brussels, Belgium;; ^k^Department of Earth and Planetary Science, University of California, Berkeley, CA 94720;; ^l^Department of Earth and Space Sciences, University of Washington, Seattle, WA 98195

**Keywords:** KPg extinction, Chicxulub, Hell Creek Formation, tsunami, impact

## Abstract

The most immediate effects of the terminal-Cretaceous Chicxulub impact, essential to understanding the global-scale environmental and biotic collapses that mark the Cretaceous–Paleogene extinction, are poorly resolved despite extensive previous work. Here, we help to resolve this by describing a rapidly emplaced, high-energy onshore surge deposit from the terrestrial Hell Creek Formation in Montana. Associated ejecta and a cap of iridium-rich impactite reveal that its emplacement coincided with the Chicxulub event. Acipenseriform fish, densely packed in the deposit, contain ejecta spherules in their gills and were buried by an inland-directed surge that inundated a deeply incised river channel before accretion of the fine-grained impactite. Although this deposit displays all of the physical characteristics of a tsunami runup, the timing (<1 hour postimpact) is instead consistent with the arrival of strong seismic waves from the magnitude M_w_ ∼10 to 11 earthquake generated by the Chicxulub impact, identifying a seismically coupled seiche inundation as the likely cause. Our findings present high-resolution chronology of the immediate aftereffects of the Chicxulub impact event in the Western Interior, and report an impact-triggered onshore mix of marine and terrestrial sedimentation—potentially a significant advancement for eventually resolving both the complex dynamics of debris ejection and the full nature and extent of biotic disruptions that took place in the first moments postimpact.

## Significance

The Chicxulub impact played a crucial role in the Cretaceous–Paleogene extinction. However the earliest postimpact effects, critical to fully decode the profound influence on Earth’s biota, are poorly understood due to a lack of high-temporal-resolution contemporaneous deposits. The Tanis site, which preserves a rapidly deposited, ejecta-bearing bed in the Hell Creek Formation, helps to resolve that long-standing deficit. Emplaced immediately (minutes to hours) after impact, Tanis provides a postimpact “snapshot,” including ejecta accretion and faunal mass death, advancing our understanding of the immediate effects of the Chicxulub impact. Moreover, we demonstrate that the depositional event, calculated to have coincided with the arrival of seismic waves from Chicxulub, likely resulted from a seismically coupled local seiche.

The Chicxulub meteoric impact marks the end of the Cretaceous and the onset of profound planet-scale climatic changes that initiated a mass extinction in the earliest Cenozoic (1, 2). Intimately associated with the third-greatest global extinction, a variety of immediate and protracted results have been proposed for the Chicxulub impact, including atmospheric perturbations and long-term global climatic shifts (3), possible impact-induced volcanism (4), and eventual worldwide ecological collapse (1). More-instantaneous effects, much more poorly resolved, include seismic disturbances (5–7) and the triggering of seiches (harmonic waves that can develop in large bodies of water) and megatsunami (8–10). Some of the most visually apparent disturbances are the tsunami/seiches recorded in high-energy sediment packages up to 9 m thick in marine deposits throughout the Gulf Coastal Plain and Caribbean (8–10). It is problematic, however, to trace their geographic extent in the Western Interior Seaway (WIS) because the terminal-Cretaceous geologic record for that depositional system is not preserved. In addition, evidence of onshore inundation by Chicxulub tsunami is thus-far unknown.

**Fig. 1.**
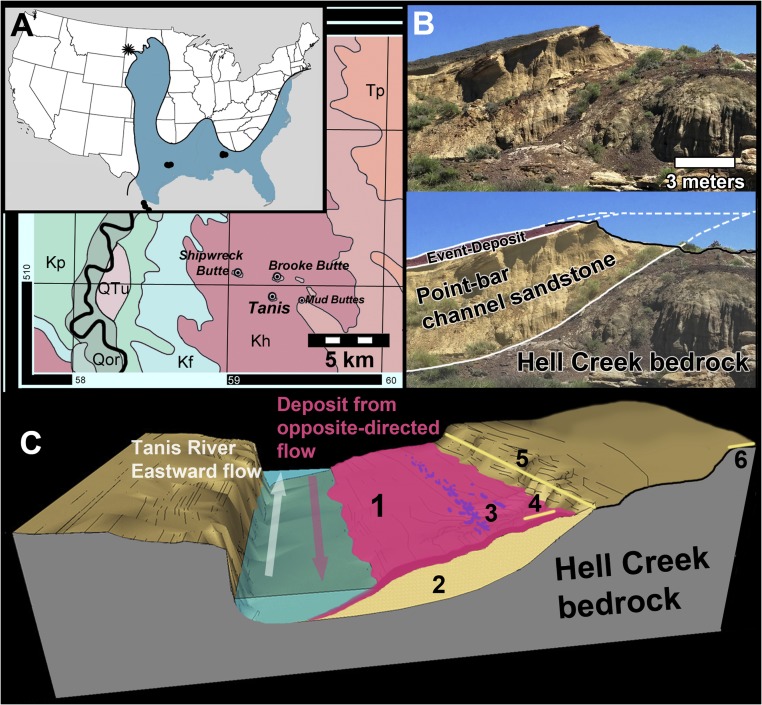
Map of the Tanis study locality. (*A*) Tanis within a regional context (large map) and on a national map (*Inset*). Reprinted with permission from ref. 14; black dots in *Inset* are previously documented KPg tsunami localities; star denotes Tanis. Kf, Fox Hills Formation; Kh, Hell Creek Formation; Kp, Pierre Shale; Qor, Holocene; QTu, Quaternary and Upper Tertiary; Tp, Slope Formation. (*B*) Photo and interpretive overlay of an oblique cross-section through Tanis, showing the contact between the angled point-bar sandstone and the gray Hell Creek bedrock. (*C*) Simplified schematic depicting the general contemporaneous depositional setting for the Event Deposit (not to scale). The Event Deposit (1) covers the slope of a prograding point bar of a meander (2), which incised into the Hell Creek bedrock during the late Cretaceous. Location of the densest carcass accumulations (3) along the slope; location of KPg boundary tonstein directly overlying the Event Deposit (4); location of KPg tonstein overlying the adjacent overbank (5); location of Brooke Butte (6), the closest KPg outcrop to Tanis.

Regrettably, in the geologic record, there is a lack of coeval records with high temporal resolution on the scale of minutes to hours. Consequently, and despite voluminous previous work on the Chicxulub impact, a full understanding of the effects and ecological impact during the first hours or days postimpact has not been resolved. Here, we report the Tanis site, which documents a turbulently deposited, rapidly emplaced sediment package directly overlain by the Cretaceous–Paleogene (KPg) boundary tonstein. The site, situated in the continental Hell Creek Formation in southwestern North Dakota (Fig. 1), displays inland-directed flow indicators and holds a mixture of Late Cretaceous marine and continental biota, implying that its emplacement is related to sudden onshore inundation surges. A suite of ejecta types, including ejecta spherules preserved within the deposit sediments (captured by the gills of fish entombed within the deposit and preserved as unaltered glassy spherules embedded in amber), indicate that deposition occurred shortly after a major bolide impact. Unaltered impact-melt glass exhibits a clear geochemical and geochronological link with the Chicxulub impact. A well-defined cap of iridium-bearing, fine-grained impactite tonstein directly overlying the deposit provides a well-constrained chronology—that is, after impact but before the finest ejecta settled—that can provide a detailed record of conditions shortly after the impact. The time frame indicated by the embedded ejecta and capping tonstein at Tanis overlaps with arrival times calculated for seismic waves generated by the Chicxulub impact, a peculiar coincidence that suggests the impact played a causative role in triggering the Tanis depositional event. Tanis is noteworthy in recording a brief period of time that directly followed (within tens of minutes to hours) the Chicxulub impact. Furthermore, the possibly impact-triggered depositional event is a phenomenon thus-far undocumented in continental facies. The Tanis site therefore provides another dimension to our understanding of how the Chicxulub impact could have affected life on Earth.

## Geologic Setting

The continental Hell Creek Formation is a Late Maastrichtian wedge of clastic fluvial sediments that prograded eastward into the epicontinental WIS during the last ∼1.3 My of the Cretaceous (11). The sediments comprise alternating bedded floodplain mudstones, paleosols, and crevasse splays, punctuated by point-bar sandstones from numerous incised meandering rivers flowing east into the WIS (12). While the geologic record of the WIS is missing beyond the Upper Campanian/mid-Upper Maastrichtian (13–15), most reconstructions support a long, narrow seaway still connected to the paleo-Gulf of Mexico at the close of the Cretaceous (e.g., refs. 13–17; Fig. 1). Cephalopod assemblages in the Pierre Shale strongly support a persistent interchange with southern taxa, indicating a sizable Late Maastrichtian corridor to the Gulf of Mexico (13) that lasted until the end of the Cretaceous. Persistent marine influence throughout the upper Hell Creek Formation, supported by marine and brackish fossils found as far west as the Little Missouri River at the Montana–North Dakota border (west of Tanis) and as far east as Bismarck, North Dakota (over 250 km to the east), as well as two marine incursions—the Breien and Cantapeta transgressions—indicate that the fluctuating, reticulated terminal-Cretaceous shoreline was not far away from the Tanis region in southwestern North Dakota (15, 18–22).

The KPg boundary is a synchronous benchmark in the geologic record, where it is delineated by a global ejecta layer composed of fine impact-derived material that began settling out from the atmosphere shortly after impact (1, 2). This ejecta layer, known locally in the Western Interior of the United States as the KPg tonstein, is the clearest and most precise marker that divides the Cretaceous and the Paleogene. It is distinguished by impact-related debris, including a distinctive iridium anomaly, shocked minerals, ejecta spherules, microkrystites, nanodiamonds, and occasionally unaltered impact-melt glass (2, 23). In the Western Interior, the KPg tonstein is easily recognized due to its conspicuous contrast in color and texture from the bounding fluvial and overbank sediments and its stratigraphic placement between uppermost Cretaceous paleosol and a thin, basal Paleogene lignite/carbonaceous shale. The KPg tonstein thus enables correlation of the KPg boundary over long distances. In southwestern North Dakota, as with the rest of the Western Interior, the KPg boundary always occurs either precisely at the Fort Union–Hell Creek formational contact, or slightly above (11). When ideally preserved, the KPg boundary clay in the Western Interior manifests as a 1- to 2-cm compact peach-colored, dual-layered tonstein clay bed (2, 23). The lower layer, which can vary from 0.7 to 1.7 cm in the local study region of southwestern North Dakota, consists of the coarsest material, such as ejecta spherules, whereas the fine-grained upper layer, which can vary from 4 mm to ∼1 cm in the local study region, contains an enrichment of platinum group elements (PGEs) such as iridium as well as the bulk of shocked minerals. However, at most localities in the study region, impact-generated debris is rare and often completely absent, rendering the KPg boundary identifiable only via biostratigraphy (11).

## Tanis Event Deposit

At Tanis, the Cretaceous and Paleogene strata are separated by the Event Deposit, a high-energy clastic sediment package immediately underlying the in situ KPg tonstein. The Event Deposit is a ∼1.3-m-thick bed that shows an overall grading upward from coarse sand to fine silt/clay and is associated with a deeply incised, large meandering river that flowed eastward during the latest Cretaceous. The deposit sharply overlies the aggrading surface of a point bar in a drapelike fashion (Fig. 1 and *SI Appendix*, Fig. S1) and is further characterized by bidirectional flow and a transition from upper- to lower-flow–regime sedimentary structures. The underlying point bar is characterized by epsilon cross-stratification, moderately to well-defined lateral accretion tabulae, and thickness exceeding typical crevasse splay deposits, features that define the point-bar deposits common throughout the Hell Creek Formation (12). The channel of the Tanis River incised deeply into the underlying strata, similar to other Hell Creek-incised channels and comparable to modern subtropical rivers (*SI Appendix*, Fig. S2). In the extant state of preservation, the point bar exhibits ∼10.5 m of isochronous elevation change along its inclined surface and its width extends <50 m perpendicular to paleoflow direction. These dimensions are in the upper size range for point bars in the Hell Creek Formation (12) and compare favorably with analogous modern rivers with large channels that are tens to hundreds of meters wide (e.g., Suwannee, Alapaha). The Tanis point bar also shows pedogenic and bioturbational signs of prolonged subaerial exposure until immediately before being covered by the Event-Deposit sediments (see below and *SI Appendix*).

**Fig. 2.**
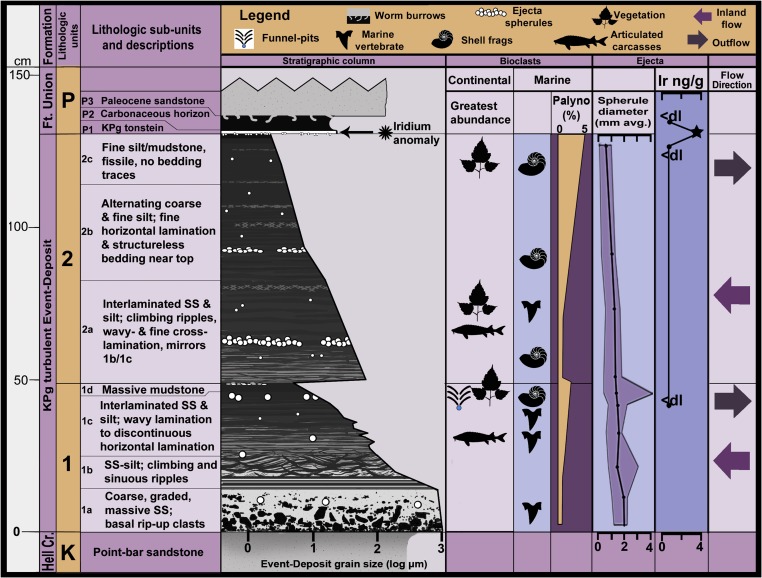
Tanis site stratigraphy and fossil distribution. Stratigraphic section of Tanis, outlining the lithological subdivisions and grain-size profile for the Event Deposit, abundance and primary stratigraphic distribution for a selection of continental and marine fossils, abundance of marine palynomorphs (palyno %), select impact-derived materials, and flow direction.

**Fig. 3.**
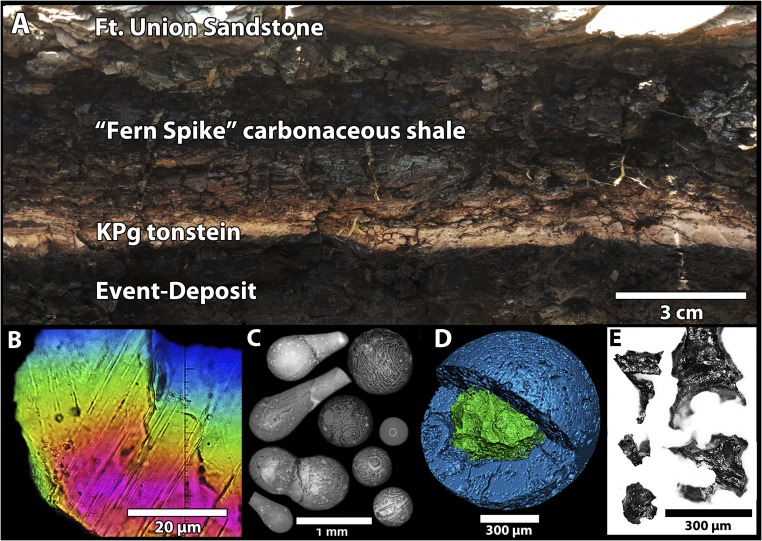
Chicxulub tonstein capping the Event Deposit at Tanis and representative impact-derived materials. (*A*) Iridium-enriched tonstein in situ atop the Event Deposit. (*B*) Shocked mineral with multiple intersecting planar deformation features (FAU.DGS.ND.161.977.T). (*C*) Clay-altered ejecta spherules (FAU.DGS.ND.161.33.T), some with prominent schlieren. (*D*) Micro-CT of a clay-altered ejecta spherule with unaltered glass core (FAU.DGS.ND.161.11.T). (*E*) Shards of unaltered impact glass (FAU.DGS.ND.161.45.T).

The Event Deposit (Fig. 2) is subdivided into two graded subunits based on a minor, abrupt increase in grain size, showing evidence for at least two successive surges. The ∼50-cm-thick basal unit 1 sharply overlies the point-bar surface and includes biological (flow-aligned carcasses and tree trunks) and sedimentary (cross-bed foresets, asymmetry/orientation of current ripples, truncated flame structures, etc.) flow structures indicative of a westward or inland flow direction that is opposite of the natural paleocurrent of the contemporaneous Tanis River. The base of unit 1 comprises a matrix-supported, massive coarse-sand conglomerate, with angular pebble- to small boulder-sized rip-up clasts derived from the underlying Hell Creek strata. As shown in Fig. 2, the massive sand (1a) at the base of unit 1 has a sharp nongradational basal contact with the underlying point-bar surface and vertically grades into a thin (∼3-cm) zone of plane-parallel bedding of interlaminated sand–silt (lower 1b); climbing ripples (mid 1b); sinuous, wavy lamination (upper 1b); low-angle cross-lamination (lower 1c); fine, discontinuous subparallel lamination (upper 1c); and nearly structureless fine silt/mud near the top (1d). Flow-direction reverses 180° toward the east—seaward—near the top of unit 1. The succeeding unit 2 resembles upper unit 1 in structure and grain size, starting with climbing ripples in an alternating sand–silt laminated interval (2a), and grading to structureless fine silt/mud at the upper terminus (2b and 2c). Flow in lower unit 2 is directed westward—inland—but reverses 180° in the upper portion, indicating eastward flow. The end of the inundation event is marked by organic-rich, fissile clay containing mats of fragmented plant matter (2c). The upper terminus is directly capped by a thin, in situ 1- to 2-cm-thick band of impactite tonstein (Figs. 2 and 3). The slightly thicker tonstein on the adjacent paleosurface of the river terrace just outside the main channel is indistinguishable from the dual-layered KPg ejecta layer elsewhere in the Western Interior; the lower layer is dominated by impact spherules and the upper layer is iridium rich (3.8 ppb) and contains the bulk of the shocked minerals (23). The tonstein on top of the Event Deposit represents mainly the upper part of the dual layer, with most of the spherule abundance distributed throughout the Event Deposit beneath. The tonstein is overlain by a thin (∼6-cm-thick) lignitic horizon of plant remains. The Event Deposit and underlying strata represent the typical Hell Creek *Wodehouseia spinata* palynofacies (24), and a “fern spike” within a depauperate palynofacies (25) characterizes the lignitic horizon.

Composition and structure of the Event Deposit and subjacent point bar imply an abrupt inundation of a paleosurface that was subaerially exposed for considerable time before deposition. Prolonged subaerial exposure is corroborated by colonization of the point bar by terrestrial organisms, open burrows that are filled with sediment from the overlying basal Event Deposit, pedogenic structures, and invertebrate fossils found entombed in some of the burrows. The sharp, nongradational basal contact with the underlying point-bar surface is further evidence for an abrupt event. Climbing ripples, pronounced grading of the deposit, water-escape structures, truncated flame structures, and steady vertical transition from upper- to lower-flow–regime flow structures provide additional proof that accumulation of the sediment package was brief and episodic, rapidly emplaced out of a dense suspension load (26). Coarse grain size, abundant subangular rip-up clasts, and imbricated debris in the basal portion indicate that the initial stages of deposition were rapid, turbulent, and high energy.

The minimum estimated runup height of the inundation was great, at least ∼10 m based on the observed paleorelief of the point bar underlying the Event Deposit (*SI Appendix*, Fig. S1). The sizable runup height, large-scale bidirectional flow, and thick draped sediment package at Tanis are unusual for the Hell Creek Formation. In addition, Tanis exhibits a depositional scenario that was unusual in being highly conducive to exceptional (largely three-dimensional) preservation of many articulated carcasses (Konservat-Lagerstätte). Such Konservat-Lagerstätten are rare occurrences in the fossil record because they require special depositional circumstances. Since Tanis is the only known site in the Hell Creek Formation where such conditions were met, the deposit attests to the exceptional nature of the depositional episode.

The lithology, entombed marine invertebrates, fossil preservation, and chronology of Tanis each preclude correlation with either the Cantapeta or Breien marine incursions (18, 19). These marine tongues transgressed over broad swaths of the Hell Creek, while the Tanis Event Deposit is restricted to a paleo-river valley and is conspicuously absent from the adjacent floodplains. The glauconitic lithology and graded basal contact of the marine tongues, indistinguishable from the Fox Hills Formation (19), also differ from the laminated sand–silt lithology and sharp basal contact of the Event Deposit. Tanis lacks *Ophiomorpha*, a trace fossil ubiquitous in the Hell Creek marine tongues (18, 19), and the Tanis marine mollusks consist almost exclusively of the ammonite *Sphenodiscus lobatus*, in sharp contrast with the *Crassostrea*- and *Corbicula*-dominated Breien and Cantapeta, where *S. lobatus* is absent (18, 19). Nacreous mollusk shell preservation at Tanis also contrasts with the poorly preserved, primarily limonitic steinkerns (internal casts of mollusks) from the marine tongues (19). The KPg tonstein overlying the ejecta-bearing deposit constrains Tanis to shortly after impact, technically the basal-most minutes/hours of the Paleogene. Biostratigraphy (megafloral and palynological) retains a terminal-Cretaceous signature, therefore in agreement with a depositional event that occurred precisely at the Cretaceous–Paleogene transition. This chronology is considerably younger than the Cantapeta and Breien marine sequences, which invaded the upper (but not uppermost) and the lower to middle parts of the Hell Creek Formation, respectively (11, 18, 19). Consequently, the Tanis Event Deposit cannot be correlated with the known Hell Creek marine transgressions.

## Ejecta, Connection with Chicxulub, and Chronology of the Deposit

**Fig. 4.**
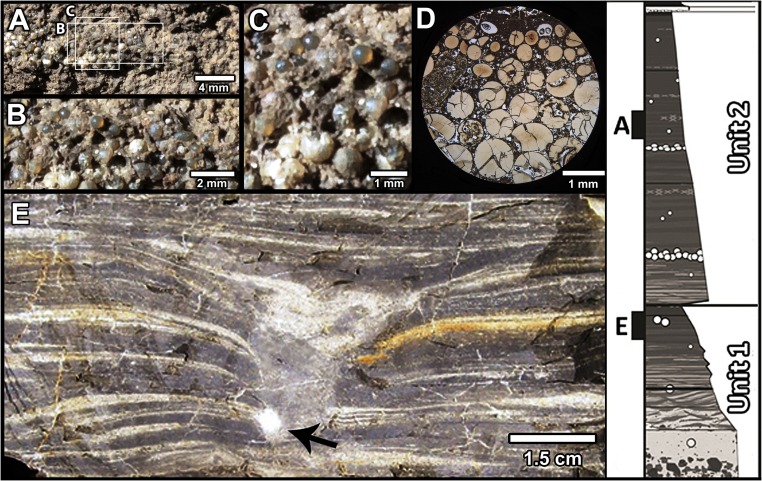
In situ ejecta at Tanis. (*A*–*C*) Field photos of an ejecta lens in situ. (*D*) Petrographic thin section of a spherule lens (FAU.DGS.ND.161.88.T). (*E*) Cross-section of down-warped “microcrater” caused by incoming ejecta, with arrow pointing to spherule (FAU.DGS.ND.161.65.T). (*Right*) Region of origin for the items pictured.

**Fig. 5.**
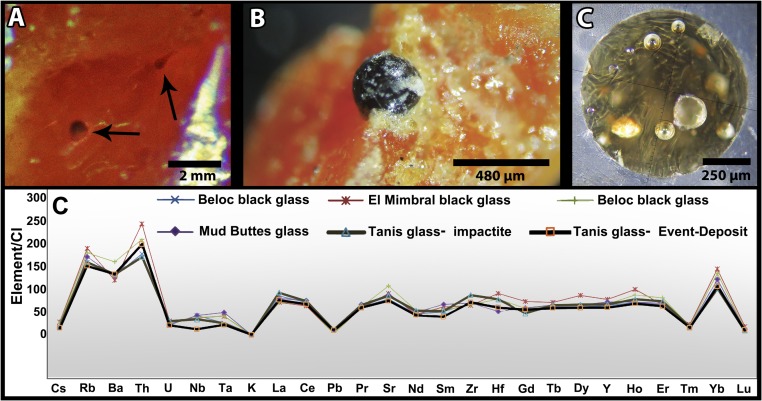
Chicxulub impact glass from Tanis, and geochemical comparison. (*A*) Magnified view of spherules within amber (FAU.DGS.ND.161.77.T). (*B*) Exposed unaltered spherule, in situ within amber (FAU.DGS.ND.161.735.T). (*C*) Thin section of unaltered glassy spherule recovered from amber (FAU.DGS.ND.161.997.T). (*D*) CI-normalized geochemistry highlighting the strong match between the ranges for Chicxulub black glass and the Tanis specimens.

Our assumption that deposition at Tanis occurred immediately after a large meteoric impact is substantiated by the following (Figs. 3–5): (*i*) ejecta spherules, (*ii*) microkrystites, (*iii*) shocked minerals with multiple intersecting sets of planar deformation features, (*iv*) unaltered impact-melt glass, and (*v*) an iridium anomaly (3.8 ppb) within the fine-grained tonstein capping the Event Deposit. Each of these features is independently a clear signature of impact and reaffirms an impact event shortly before the Tanis depositional event. These impact-derived materials are absent from the bounding strata below the Event Deposit and above the tonstein.

Splash-form and round ejecta spherules (*SI Appendix*, Fig. S10), the majority ranging from 0.3 to 1.4 mm in diameter, occur throughout the Tanis deposit, with a few at the contact between the capping KPg tonstein and the underlying Event Deposit. By comparison, spherules from ∼3,000 km away from the crater, in Gorgonilla Island, Colombia, average about 1 mm in diameter (27); those from ∼1,700 km away at Blake Nose, western North Atlantic, average 1 to 3 mm (28); those from ∼1,000 km away in Beloc, Haiti, average 3 to 4 mm, up to 8 mm or more (8); and those from ∼1,044 km away in El Mimbral, Mexico, average 3 to 5 mm, with blebs up to 15 mm (9). Although there is not a perfectly linear relationship between spherule size and distance from the crater (e.g., spherules at El Mimbral can be larger than some examples from closer to the crater, in Beloc), spherules show a general decrease in size with distance. The most common spherule size range at Tanis (0.3 to 1.4 mm) is reminiscent of the average size expected in the Western Interior relative to distance from the crater and is comparable to ejecta from nearby outcrops. The infrequent, large-sized outliers at Tanis are similar in size to the lowest size ranges from proximal localities such as El Mimbral and Beloc. However, they are much smaller than the average or largest examples from those localities.

Although repeated reworking might explain the isolated spherule occurrences in the deposit, other observations suggest that primary air-fall deposition has been recorded in several horizons. Spherules occasionally occur in thin, close-packed, size-graded lenses (Fig. 4 *A*–*D*) in upper unit 1 and unit 2. Spherules in the graded lenses far exceed the surrounding fine grain size and therefore exceeded transport capacity. This is supported by the fact that they are not accompanied by a coarser-grained influx, which would have signified a pulse in flow velocity that could have carried them in. Flow velocity sufficient to carry the spherules would have eroded the fine-grained contextual sediment and hampered its deposition; therefore, it is unlikely that the spherules in the lenses were transported or reworked subsequent to deposition. Rather, these observations indicate that they were deposited directly after settling through the air and/or water column, and thus their deposition should align closely with ejecta arrival times based on their ballistic trajectories.

As a second potential example of primary deposition of ejecta, the contact zone between units 1 and 2 contains scattered, uncommon, 3- to 8-cm-deep funnel-like cones of down-warped laminations, which typically contain a single, unusually large (∼3-mm) spherule at their base (Fig. 4*E* and *SI Appendix*, Fig. S12). These warped structures are rare, as fewer than 15 were recovered in >400 m^3^ volume of excavated sediment, and are overlain by undisturbed Event-Deposit sediment, indicating that they were produced syndepositionally. Spherules settling out of suspension are unlikely to have created such warped depressions, but rather, the 3- to 8-cm penetration and down-warping suggest that a descending spherule fell at terminal velocity on an exposed, soft surface between the two main surge pulses or, at most, was covered by a few centimeters of water.

**Fig. 6.**
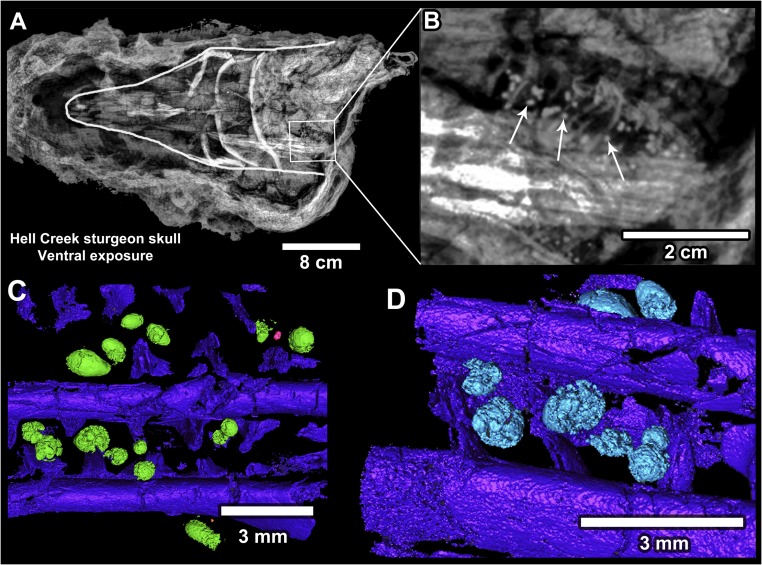
Acipenseriform fish with ejecta clustered in the gill region. (*A*) X-ray of a fossil sturgeon head (outlined, pointing left; FAU.DGS.ND.161.115.T). (*B*) Magnified image of the X-ray in *A* showing numerous ejecta spherules clustered within the gill region (arrows). (*C* and *D*) Micro-CT images of another fish specimen (paddlefish; FAU.DGS.ND.161.29.T), with microtektites embedded between the gill rakers in the same fashion.

Additional spherules were recovered from amber blebs attached to tree trunks or large branches (Fig. 5 and *SI Appendix*, Fig. S14). Those spherules are completely unaltered impact glass, shielded from chemical weathering by their enclosure in amber. Even more remarkable are spherules concentrated in the gill rakers of more than 50% of acipenseriform (sturgeon and paddlefish) fish carcasses within the deposit (Fig. 6 and *SI Appendix*, Fig. S15). Passive suspension feeding is a common specialization among some acipenseriforms (i.e., certain paddlefish taxa), which sieve food with their gills while swimming open-mouthed (29). It is most likely that the Tanis paddlefish inadvertently aspirated the spherules by this mechanism when the ejecta settled through the water column. Spherules within the fish carcasses at Tanis suggest that the impact event and associated macrofossils were temporally correlated.

The geochemistry and radiometric age of unaltered impact glass from Tanis directly correlate with the Chicxulub impact. Although most Tanis spherules are diagenetically altered to smectitic clay, some very rare spherules still contain a core of unaltered glass (Fig. 3*F*). Tanis is only the fourth (1, 8, 27, 30) outcrop to contain unaltered Chicxulub impact glass. The glass is dark and vesicular, with pockmarked surfaces, includes some internal crystals of melilite and encapsulated debris, and has extremely low water content (0.02 to 0.03%), consistent with impact origin. The unaltered impact glass is geochemically nearly indistinguishable from the major element (*SI Appendix*, Fig. S16 and Table S3) and trace element (Fig. 5*D* and *SI Appendix*, Table S3) ranges exhibited by Chicxulub black glass (30). ^40^Ar/^39^Ar analysis of the Tanis glass yielded a weighted radiometric date of 65.76 Ma ± 0.15 My, following the calibration of Kuiper (31), identical in age with Chicxulub impact dates from elsewhere (32).

Because the Tanis deposit contains ejecta throughout and is also capped by the KPg tonstein, the depositional event took place during a narrow window of time: after impact but before deposition of the fine-grained KPg tonstein. Given this constraint, we can deduce that the Event Deposit was emplaced within a matter of hours after the Chicxulub impact event. This chronology can possibly be further constrained by the timing of incoming ejecta embedded within the deposit. The time span between ejection and deposition of primary air-fall debris is governed by the ballistic trajectory of the spherules, also taking atmospheric drag into account (5). We assume a scenario in which ejecta-curtain material, launched at about a 45° elevation angle and seen as the glassy or altered-glass spherules at Tanis, arrived before the shocked quartz that was launched at steep angles in a “warm fireball” produced by release of CO_2_ from shocked limestone after departure of the ejecta curtain (33). The travel times to Clear Creek, Colorado, and Brownie Butte, Montana (33), have been recalculated (34) for Tanis, revealing that ejecta-curtain spherules launched at assumed elevation angles of 30° to 60° reach the top of the atmosphere above Tanis from 13 to 25 min after impact. Shocked quartz from the warm fireball, launched at angles from an assumed 70° to the limit of the forbidden zone at 79°, begins to reach the atmosphere above Tanis about 38 min after impact and ceases reaching Tanis about 2 h after impact. Based on this constraint, if the ejecta embedded in the Event Deposit represents primary air-fall as suggested by its mode of deposition, then the surges arrived sometime between ∼13 min and 2 h after impact. The fine-grained KPg tonstein subsequently settled on top, beginning in the ensuing hours. This is consistent with ejecta arrival times calculated by previous studies (35).

## Biota in the Event Deposit

**Fig. 7.**
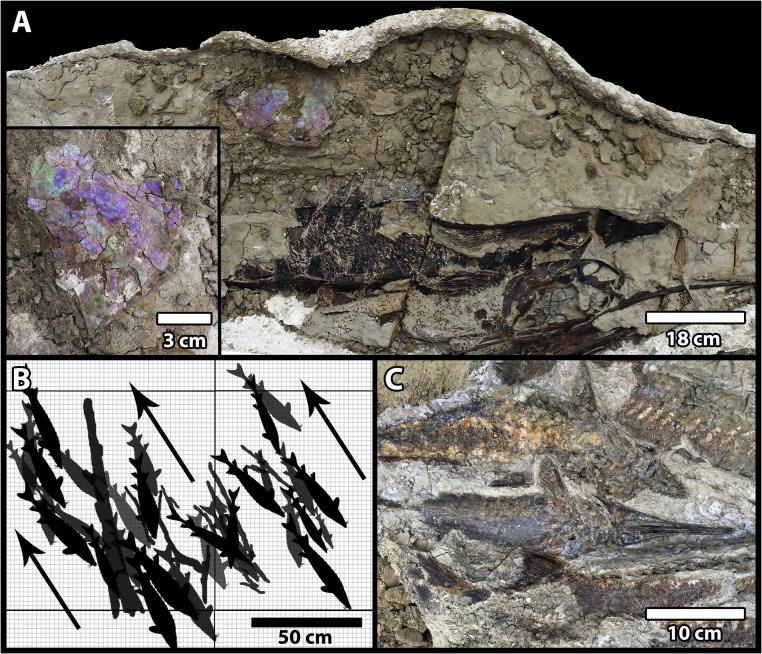
The Tanis Konservat-Lagerstätte. (*A*) Plaster field jacket with partially prepared (freshwater) acipenseriform fish (FAU.DGS.ND.161.116.T) next to a nacreous ammonite shell (*Inset*). (*B*) Partial site map showing carcasses oriented by flow. (*C*) Field photo showing mass grave of fish carcasses, aligned by flow.

Biological debris supports a rapid depositional event, and its imbrication indicates deposition via bidirectional currents. Fossil fish and logs are in some instances oriented obliquely through the Event Deposit, spanning the entire thickness. Such vertical orientation indicates that the entire deposit was emplaced quickly. A prolonged duration of time, or emplacement by multiple episodes separated by considerable time (i.e., repetitive phenomena such as tidalites), would have degraded the carcasses, contradicting what we see at Tanis. Taphonomy of the carcasses, including three-dimensional and near-identical states of preservation, demonstrate that the carcass assemblage represents a sudden mass-death accumulation, likely caused by extremely rapid burial in the fine-grained sediment. Interwoven articulated vertebrate carcasses with heads pointed toward the incoming flow direction, and elongate trunks/branches from trees that are strongly parallel-oriented by flow, support a bidirectional paleocurrent direction at the time of deposition (Fig. 7 and *SI Appendix*, Fig. S26).

The absence of scavenging despite the shallow burial of plentiful, large carcasses and the lack of root traces along the upper surface of the Event Deposit may suggest a depleted local biodiversity after deposition. Megaflora (*SI Appendix*, Fig. S20) diagnostic for the terminal Cretaceous (36), and terrestrial palynomorphs (*SI Appendix*, Fig. S21) indicating subzone E (24) of the *Wodehouseia spinata* assemblage (= uppermost Cretaceous, including the KPg boundary), are consistent with a terminal-Cretaceous event, before appearance of the first Paleogene taxa. The dense accumulation of vertebrate carcasses has not been reported in any other Hell Creek flooding event, despite the Hell Creek being a fluvially dominated depositional setting, and is reminiscent of rafted organic material associated with major inundation surges (37).

Very well-preserved isolated fragments and partial shells of (marine) ammonites (aragonitic and nacreous, no signs of dissolution), predominantly *S. lobatus*, are diffused throughout the deposit. Marine dinocysts include markers for the latest Maastrichtian (38, 39), but no Danian markers. These marine fossils contrast starkly with the many continental (= freshwater) paddlefish and sturgeon carcasses in the same strata. The mixed fossil assemblage of well-preserved continental and marine fossils supports injection of biological remains from a contemporaneous marine environment, likely the WIS, possibly combined with slightly older unconsolidated or unlithified seafloor oozes scoured during inundation. As mentioned in the *Geologic Setting* section, brackish/marine indicators existed within several to tens of kilometers from the Tanis region throughout the uppermost Hell Creek Formation, indicating that the WIS shoreline was not appreciably distant. δ^18^O values from −0.5 to −4‰ Vienna Pee Dee Belemnite (VPDB) support a brackish to fully marine origin for ammonites and vertebrate marine fossils, ruling out freshwater tolerance that occurs with certain marine taxa in the Hell Creek (e.g., *Myledaphus* ray, some orectolobiform sharks). The mix of fully marine and freshwater taxa superficially resembles the Cantapeta and Breien tongues, but as discussed in *Tanis Event Deposit*, the lithology, faunal composition, preservation, and chronology are incompatible.

## Depositional Mode

In modern fluvial depositional environments, as with the Hell Creek Formation, major high-energy depositional events or hydrological surges are related either to massive storms or to river flooding. The sedimentological features at Tanis, particularly the large-scale bidirectional flow, high runup, thick deposit, and draped sediment package of relatively even thickness, are incompatible with storms and river flooding (37, 40) and do not ally with other common terrestrial or marginal-marine depositional mechanisms such as tidalites. The depositional mode and sedimentology at Tanis compare most favorably with an inundation surge (37, 40), with the physical characteristics of a tsunami (refs. 40–42 and *SI Appendix*, Figs. S8 and S9) that could have emanated from the impact site up the WIS or, alternatively, due to a more localized seiche.

The large inland-directed surge complex at Tanis, incompatible with either river flooding or storm deposits, was a rare and unusual occurrence in the Hell Creek Formation. Because this depositional event occurred immediately after the Chicxulub impact, which itself was a rare occurrence, the two events could likely have borne a causative, rather than just a coincidental, relationship. A large-scale inundation such as the one that affected Tanis could have resulted from a variety of impact-related triggering mechanisms. Onshore inundation by a massive tsunami caused by the Chicxulub impact (i.e., propagating directly from the impact site), which has already been documented in marine facies at more proximal localities (9, 10), might have been capable of producing the sedimentary surge deposit preserved at Tanis. However, such a tsunami would have been greatly attenuated in the shallow WIS, even assuming the WIS was an uninterrupted, open corridor at that time, which is not presently known. Similarly, a locally triggered seiche could have been equally capable of onshore inundation, resulting in a sedimentological end product nearly indistinguishable from tsunamite. Seismic waves generated by the Chicxulub impact have been suggested to be powerful enough to cause (*i*) a pulse in marine volcanism at diverging plate margins (4), (*ii*) terrestrial seismic ground movements in the Western Interior (5), and (*iii*) acceleration of Deccan volcanism (7). Such seismic waves were probably also sufficient to trigger seiches at large distances. The capability for seismic shaking to trigger seiche activity is reaffirmed by historical observations, in which S waves from the lesser moment magnitude M_w_ ∼9.2 Tohoku earthquake (Japan, 2011) caused seismic seiches with an amplitude >1.5 m in Norwegian fjords nearly 8,000 km from the epicenter (43), a greater distance than between the Chicxulub crater and Tanis (∼3,000 km).

## Emplacement Mechanism

The timing and correlation of the depositional event to the impact-induced effects (seismic waves and arrival of ejecta) provide constraints for deciding which triggering scenario was most plausible. Most importantly, it appears implausible that a tsunami from the Gulf could have caused the Tanis depositional event for three principal reasons: (*i*) while the WIS is thought to have remained connected to the Gulf in the latest Cretaceous, it is unconfirmed whether a connection was, in fact, present at that time; (*ii*) the variably shallow epicontinental WIS would have greatly attenuated the tsunami waves; and (*iii*) the travel time for a tsunami from Chicxulub to Tanis would have been a minimum of 18 h based on tsunami travel calculations (*SI Appendix*).

At a paleoepicentral distance of ∼3,050 km from the center of Chicxulub, Tanis would have received P, S, and Rayleigh waves 6, 10, and 13 min after impact, respectively. A seismically induced seiche wave could have been generated soon thereafter, with constituent surge pulses each lasting tens of minutes, depending on the period of the seiche wave. (The latter cannot be determined with any precision because the average depth of the water body is not known.) The seismic wave arrivals would have been followed closely by the arrival of impact-melt spherules from the ejecta curtain. Based on ballistic trajectory calculations (5, 33, 34) and assuming that most of the spherules were ejected from Chicxulub at an angle of ∼45° to 50° from the horizontal, spherules would have begun arriving at Tanis ∼15 min postimpact. The vast majority would have fallen at Tanis within 1 to 2 h of impact. This time frame is entirely consistent with the calculated timing of a seismic seiche generated in a local arm of the WIS in the Tanis region. Thus, seismic waves from Chicxulub arrived at the Tanis region just minutes before the window of deposition and long before a tsunami from the Gulf could have reached it. The correlation in timing between the arrival of seismic waves from Chicxulub and the Tanis depositional episode supports the plausibility that seismic wave energy triggered the depositional episode.

The Chicxulub impact generated a very large earthquake, with reconstructed estimates supporting a moment magnitude in the range of M_w_ ∼10 to 11.5 (44, 45). Globally induced seiche magnitudes from historical earthquakes can be used to scale the potential maximum amplitude of a seiche at Tanis triggered by Chicxulub. For example, the great 2011 Tohoku earthquake in Japan (M_w_ ∼9.2) generated a well-documented ∼1.5 m amplitude seiche in a Norwegian fjord nearly 8,000 km from the epicenter (43). Given that seismic ground motion increases by a factor of ∼30 with every factor of 2 increase in moment magnitude, we straightforwardly infer that the Chicxulub earthquake could have easily generated seiches worldwide with amplitudes of the order 10 to 100 m. The runup height of the Tanis Event Deposit is at least 10 m, compatible with this estimated seiche magnitude and, as explained in *Ejecta, Connection with Chicxulub, and Chronology of the Deposit*, possessed the right timing based on ballistic trajectory calculations for the arrival of impact spherules at Tanis. Moreover, these calculations show that large-amplitude seiches were likely induced in enclosed or semienclosed bodies of water worldwide, and that some of the resulting deposits (e.g., ref. 46) might be mistakenly attributed to tsunami.

Additional (remote) scenarios could potentially have coincided with ejecta arrival, with unknown affects or influence on the propagation of a seiche. For example, previous studies have suggested that the temperature differential caused by interaction of ejecta with the atmosphere, which could have had a marked effect on a ∼4,000-km-diameter area around the Chicxulub crater, was capable of rapidly inducing violent meteorological events (47). The projected gale-force winds would have largely cooccurred with ejecta arrival (47) and, if so, could have affected the WIS during the same time interval as the Tanis depositional event. Similarly, strong seismic ground motion could have caused landsliding in the WIS near Tanis, resulting in a local surge deposit. Although such mechanisms have not been quantified sufficiently in terms of either amplitude or timing, they might be considered as potentially testable hypotheses in future studies. At this point, we consider a seiche to be the most obvious and best-supported mechanism to explain the Tanis Event Deposit.

Observations at Tanis expand our knowledge of the Chicxulub impact’s damaging effects and their far-reaching scope. The highly probable link between impact-induced seismic shaking and the onshore inundation surge at Tanis reveals an important additional mechanism by which the Chicxulub impact could have caused catastrophic conditions in the Western Interior, and possibly worldwide, far from the impact site. Thus, we identify a potential additional mechanism for abrupt, extensive damage to widely spaced regions and ecologies. The global extinction event, therefore, could have had a rapidly delivered precursor, both at the local and global scales, minutes after impact.

## Materials and Methods

### Analyses of Major and Trace Elements.

Geochemical analysis of ejecta was carried out at the Florida Center for Analytical Electron Microscopy and Activation Laboratories Ltd. via laser-ablation, inductively coupled plasma mass spectroscopy (LA-ICP-MS), ICP-MS, and energy-dispersive spectroscopy (EDS). LA-ICP-MS utilized a Perkin-Elmer Sciex ELAN DRC II under standard operating parameters (*SI Appendix*, Table S1), coupled to a laser-ablation unit (New Wave, 213 nm, UP-213). The data were reduced using GLITTER data reduction software (GEMOC). ICP-MS utilized a ThermoFisher Element 2, and PGE concentrations were measured following the methods of Becker et al. (48). EDS was carried out on a JEOL 8900r equipped with five wavelength-dispersive spectrometers. The measurements were counted for 10-s periods at 20 nA, 15 kV.

### Scanning Electron Microscopy.

Scanning electron microanalysis was carried out at on a JEOL 8900r operated at 15 kV, and specimens were examined via electron backscatter, secondary electron imaging, and EDS.

### Micro-CT.

Micro-computed tomography (micro-CT) analysis was performed using a 5-µm-resolution scanner via a transmission-type X-ray source operated at 70 kV and 100 µA. Resolution for each specimen was a voxel size of ∼5 to 10 µm, and 3D reconstructions and meshes were assembled in Avizo 8.1 and in the reconstruction software Octopus.

### Grain Size.

For grain-size fractions above 370 µm, traditional sieving techniques were employed; sediment finer than 370 µm was quantified using a hydrometer following standard procedures. Three identical samples from each stratigraphic sampling level were analyzed and then averaged to produce the final values. Graphs of grain-size distribution were generated for each sampled interval, and average grain sizes for each interval were graphed for the total stratigraphic thickness of the deposit.

### ^40^Ar/^39^Ar Dating.

Unaltered impact glass was dated using ^40^Ar/^39^Ar geochronology. Single grains of impact-melt glass were irradiated together with Beloc tektite and IrZ sanidine for 18 h at the Oregon State University TRIGA reactor in the cadmium-shielded cadmium-lined in-core irradiation tube facility. Sanidine from the IrZ bentonite, located a few centimeters above KPg (49) and ∼300 km northwest of Tanis, is used as standard. If the impact-melt glass from Tanis originated from the Chixculub impact, all samples (Tanis, Beloc, and IrZ) should yield the same ^40^Ar*/^39^Ar_K_ ratio corrected for small neutron flux gradients and their corresponding R value, or (^40^Ar*/^39^Ar_K_)_tektite_/(^40^Ar*/^39^Ar_K_)_IrZ_ = 1. The age calibration model (e.g., refs. 32, 50, and 51) is not relevant for this purpose, but the calibration model of ref. 32 is used to calculate ages. ^40^Ar/^39^Ar analyses were performed at the geochronology laboratory of the Vrije University Amsterdam. Single glass shards or sanidine was fused with a Synrad CO_2_ laser beam, and released gas was exposed to NP10 and St172 getters and analyzed on a Helix MC noble-gas mass spectrometer. The five argon isotopes were measured simultaneously with ^40^Ar on the H2-Faraday position with a 10^13^ Ω resistor amplifier, ^39^Ar on the H1-Faraday with a 10^13^ Ω resistor amplifier, ^38^Ar on the AX-compact discrete dynode (CDD), ^37^Ar on the L1-CDD, and ^36^Ar on the L2-CDD. Gain calibration is done by peak jumping a CO_2_ reference beam on all detectors in dynamic mode. All intensities are corrected relative to the L2 detector. Air pipettes are run every 10 h and are used for mass discrimination corrections. The atmospheric air value of 298.56 from Lee et al. (52) is used. Detailed analytical procedures for the Helix MC are described in Monster (53). The correction factors for neutron interference reactions are (2.64 ± 0.02) × 10^−4^ for (^36^Ar/^37^Ar)_Ca_, (6.73 ± 0.04) × 10^−4^ for (^39^Ar/^37^Ar)_Ca_, (1.21 ± 0.003) × 10^−2^ for (^38^Ar/^39^Ar)_K_, and (8.6 ± 0.7) × 10^−4^ for (^40^Ar/^39^Ar)_K_. All errors are quoted at the 2σ level and include all analytical errors. All relevant analytical data for age calculations are found in *SI Appendix*, Table S2.

### Light Microscopy, Thin Sections.

Thin sections for optical microscopy and electron microprobe analysis were prepared by National Petrographic Services, Houston, Texas, using standard procedures. Initial observations were made with an Optima ZM-160AT dissecting scope and an Ernst Leitz Wetzlar light microscope; traditional petrographic observations were made with an Olympus BH2 and Leica DM750P in normal, polarized, and cross-polarized light.

### Palynology.

Palynological slides were prepared by Global Geolabs Ltd., Medicine Hat, Canada, using standard palynological processing procedures. A 10% solution of HCl was added to each polypropylene beaker of sediment. After dissolution of carbonate fractions, HCl was decanted and replaced with distilled water, which was decanted and replaced several times to remove any remaining calcium ions. A 70% solution of HF was added before centrifuging at 4,450 × *g* for 5 min. After removal of HF and neutralization of the residue, 25 mL of ZnBr_2_ was added, and the tube was ultrasonicated for approximately 10 s. Specimens were allowed to sit for 10 min and then centrifuged at 4,450 × *g* for 15 min. Buoyant “float” was removed and centrifuged for an additional 2 min. In a 20-mL glass tube, 3 mL of Schultz solution was added, vortex-mixed, and placed in hot bath. Schultz solution was removed and neutralized via multiple steps of centrifuging and washing, and a 10% solution of NN_4_OH was added for 2 min. This was then neutralized via multiple steps of centrifuging and washing. Note: the marine dinocysts are incredibly fragile and thin walled; they can degrade rapidly with excessive use of this last oxidation step, leading to underrepresentation in the finished slides. The sieved fractions were pipetted onto a slide and mixed with polyvinyl alcohol. After drying of the polyvinyl alcohol, one drop of clear casting resin was added, followed by a coverslip.

### Isotope Geochemistry.

Powdered samples were analyzed with a Gas Bench II linked to a Thermo Finnigan duel-inlet MAT 253 Stable Isotope Ratio Mass Spectrometer. Isotopic data were reported according to the VPDB international standard, with analytical precision to ±0.4‰.

### Isolation of Ejecta Particles.

Ejecta spherules and shocked quartz were primarily retrieved via bulk sediment processing using standard sieving procedures, but this led to underrepresentation of spherules due to tendency of the delicate smectite to fracture and disintegrate when subjected to the rigors of disaggregating, washing, and sieving. When practical, spherules and spherule lenses were also handpicked from the outcrop or gills of fish carcasses during the extensive excavations. Relict impact glass and shocked quartz were recovered through sieving, and glass was also directly removed from the cores of some partially altered clay spherules. Sieved sediment ≤500 µm was subjected to magnetic separation to recover microkrystites. Several ejecta spherules were isolated from amber specimens via gentle crushing of the amber with a wooden laboratory spatula, and the remaining amber fragments were saved in a clean vial for later analysis.

## Supplementary Material

Supplementary File
