# Supplementary material for: A seismically induced onshore surge deposit at the KPg boundary, North Dakota
Source: Proc Natl Acad Sci U S A. 2019 Apr 1;116(17):8190–9. doi: 10.1073/pnas.1817407116 (PMC6486721; doi:10.1073/pnas.1817407116)
Supplement: Supplementary File [file pnas.1817407116.sapp.pdf]

Supplementary Information for

A SEISMICALLY INDUCED ONSHORE SURGE DEPOSIT AT THE KPG BOUNDARY, NORTH DAKOTA

Robert A. DePalma, Jan Smit, David A. Burnham, Klaudia Kuiper, Phillip L. Manning, Anton Oleinik, Peter Larson, Florentin J. Maurrasse, Johan Vellekoop, Mark A. Richards, Loren Gurche, Walter Alvarez

Correspondence to: Robert A. DePalma, Rdepalma@pbmnh.org; David A. Burnham, Dinosaur@ku.edu.

This PDF file includes:

Supplementary text
Figs. S1 to S31
Tables S1 to S7
References 1-74

Tanis equivalents, and infrequency of their discovery in the local fossil record

Tanis study area and local context. A deposit similar to Tanis has thus-far not been reported in the terminal-Cretaceous sediments of the Western Interior Seaway for good reason- there is no geologic record of the WIS during that time interval. Far east of Tanis, exposures of the Hell Creek Formation exist toward the direction of the WIS, however most or all of them

predate the KPg boundary. The Tanis depositional event, as interpreted, would have affected a scattering of localized areas, and not a broad-swathed region. Because of this, and partially due to incompleteness or inaccessibility of the geologic record, Tanis is a rarity in the preserved terminal-Cretaceous portions of the study region. Therefore, the odds of randomly finding a similar deposit are low. Complicating this is the incompleteness of coeval strata in immediate area; KPg boundary outcrops are preserved as isolated, widely spaced buttes. In the study region, there are no preserved remains of large rivers or bodies of water susceptible to seiche excitation that intersect the KPg boundary. The nearest local KPg sections are Brooke Butte (~1.1 km north) and Mud Buttes (~2.2 km east). At these localities, as with virtually all other regional KPg boundary localities, the boundary is preserved in overbank and floodplain facies, areas that would not have been susceptible to a Tanis-like inundation. In addition, because channels in the Hell Creek cannot easily be correlated or traced over long distances (***1, 2***), it may prove impossible or impractical to trace the Tanis tract beyond the research site, if it has not already been obliterated by erosion. In addition, the Tanis site, itself, went so long without being discovered in part because the private sections of the ranch were closed to academic research for many years due to friction between the landowners and a local research group that had long been working in the immediate area. This left the area underexplored by academic researchers for considerable time. Otherwise, the site would have been quick to identify due to its unusual nature, particularly because of its uncommon sedimentology and the conspicuous assemblage of many exposed, articulated fossil fish that are rare in the Hell Creek Formation (until this study, fewer than 5 articulated fish fossils were known from the Hell Creek [e.g. ***3***], as opposed to the hundreds/thousands at Tanis).

Sedimentology and stratigraphy of Tanis

Point-bar surface of the Tanis River. The prograding slope of the Tanis point-bar directly underlies the sheet-like, graded sequence of the Event-Deposit (**Figs. 1, S1**). Areal extent of the entire site is limited to approximately 2 acres due to limited exposure and erosion of coeval strata. The point-bar is associated with a deeply incised river that was situated near the WIS coastline during the latest-Cretaceous (**Fig. 1**). The Tanis River incised deeply into the underlying Hell Creek mud-, silt-, and sand-dominated sediments, and the point-bar sand body is approximately 11.5 meters thick at the thickest observable exposure. It is characterized by epsilon cross-stratification (***1***), and an overall fining-upward trend. Composition is weakly cemented fine-grained yellow-tan sand, with occasional indurated dark orange-brown benches along the angled lateral

accretionary tabulae (**Fig. 1**). Flow indicators, including cross-bed dip angle, asymmetry and orientation of current ripples, and occasional biological debris (e.g. ***4, 5***), indicate a paleocurrent of approximately S 50-75 E for the Tanis River.

Overall characteristics of structure and size for the sand body beneath Tanis present a classic example of a laterally accreting, epsilon cross-stratified point-bar as commonly preserved throughout the Hell Creek Formation (***1***). It does not resemble either a crevasse-splay or thalweg-fill, the two other dominant types of sand bodies in the Hell Creek (***1***). The thickness exceeds the that of typical Hell Creek crevasse-splay or thalweg fills (***1***), and is in the upper size range for the average Hell Creek point-bars, which typically range from 10 to 20 meters thick (***1***), indicating a sizable paleo channel of the Tanis River. Scatterings of sparse plant debris, occasionally present along the planar surfaces of the epsilon tabulae at Tanis, are common in point-bar deposits, as are patches exhibiting pedogenic structures, which occur in upper regions of point-bars that are stable enough to support vegetative growth (***1***). The flow direction indicated by the Tanis point-bar, trending southeast, was almost universal for all large rivers during Hell Creek times (***1***), which emptied eastward into the WIS.

The prograding slope of the point-bar takes on a faint stair-step profile, leading to the development of intermittent terrace paleosols (**Fig. S1**), a common feature of point-bar deposits (***1***). The localized terrace deposits bear incipient pedogenic structures such as bedding traces obscured by slickensides, root traces, burrows from terrestrial invertebrates, and mottled coloration due to oxidation/reduction. Most burrows bear oxidized reddish-orange walls indicating prolonged subaerial exposure and moderately well-drained conditions. Most of the burrows show evidence of being occupied for a prolonged period due to their complexity and overprinting. Burrows were open up until the time of inundation and were rapidly covered by a sudden event, leaving little to no time to escape, as evidenced by in-filled sediment contiguous with the basal portion of the overlying Event-Deposit and occasional traces of their original occupants. The most extensive burrow complexes were found in the mid portions of the point-bar, and have tunnels or galleries that penetrate downward over 2 meters, indicating that they had not yet reached the water table.

Large river systems are the primary cause of steep variations in paleotopography in the Hell Creek Formation, which is generally rather flat, comparable to the modern U.S Gulf coastal Plains. Terminal-Cretaceous paleotopography in southwestern North Dakota near Tanis follows this trend, and most KPg boundary sections occur at similar vertical

elevations. Some of the larger Hell Creek rivers cut tens of meters into the bedrock (***6***), and point-bar deposits from incised channels are common throughout the Hell Creek Formation (***1, 2***), including multiple large examples in the Tanis vicinity. The incised channel depositional setting at Tanis led to the emplacement of latest-Cretaceous sediments at a vertical elevation slightly lower than that of the nearest correlating units at Brooke Butte (~1.1 km to the north) and Mud Buttes (~2.2 km southeast). Elevation of the local KPg tonstein is variable even within the same outcrop; at Brooke Butte, the undisturbed, in situ KPg tonstein differs in elevation by more than 5 meters at different locations along the butte, and differs by over 12 meters in areas affected by massive slump-faulting at the same locality. Pearson et al. (*7*) noted "A channel large enough to concentrate the original flood-plain biota cannot be constrained within the sediments of the highest few meters of the Cretaceous. Such large channels would have…eroded more deeply into the Cretaceous rock, resulting in a lower observed stratigraphic position…" At Tanis there is an observed ~10.6 meter change in uninterrupted, isochronous paleorelief, the highest point approximately 9.4 meters below the KPg boundary at Brooke Butte **(Fig. S2**), and 3 to 7 meters below the KPg boundary at Mud Buttes. This extent of elevation change is well represented in the fossil record (i.e. ***6***), as well as modern subtropical rivers, which can demonstrate even greater elevation change than at Tanis over an identical distance (**Fig. S2**). Biostratigraphy (see below) corroborates that the Tanis event occurred at the Cretaceous-Paleogene boundary and was not part of the lower Hell Creek sequence.

Unit 1 of the Event-Deposit. Unit 1 (**Fig. S5**) is a massive, normally graded bed, approximately 50 cm thick, and sharply overlies the point-bar paleosurface. Broadly, Unit 1 grades from coarse sand-dominated at its base, to interlaminated fine sand-silt, and then fine silt-dominated in its uppermost portion (**Figs. 2, S4, 5**), visible as a distinct color change that grades from buff-tan in the lower portions, to dark grey in the upper portions. Whereas the whole of Unit 1 forms a relatively consistent drape over the angled paleosurface of the point-bar, the lower, sand-dominated portions (1a-b) gradually thin up-slope and pinch out in the upper 1/3 of the point-bar, while the silt-dominated portions (1c, d) continue up-slope to the maximum observed extent of run-up. Flow indicators such as dip direction of cross-bed foresets, climbing ripples, and direction of truncated flame structures (**Fig. S7**), indicate a preferred paleocurrent direction of ~N 60-64 W for most of Unit 1 (1a-c), and nearly 180° opposite (~S 62-66 E) for Unit 1d. This is in agreement with flow indicators from vertebrate and invertebrate biological debris (see below).

Basal Unit 1 (1a) is ~13 to 27 cm thick and is characterized by coarse-grained sand and plentiful angular to sub-angular rip-up clasts in a matrix-supported conglomerate, with virtually no sedimentary structures. Rip-up clasts (**Fig. S5**), primarily composed of silt- and mud-dominated paleosols, typically range from ~5 mm to 15 cm, with some exceeding 25 cm. They are chaotically oriented, and largely constrained to the lowermost portion of 1a. The successive sub-unit (1b), ~10 to 15 cm thick, is also sand-dominated, however is subdivided from 1a by containing a higher content of silt, better-defined sedimentary structures, and no rip-up clasts. Basal 1b is fully sand-dominated and characterized by a thin (~4 cm thick) zone of discontinuous plane-parallel bedding comprised of medium- to fine-grained sand interlaminated with very coarse silt, followed by abundant cross-bedding and climbing ripples. Sets of climbing ripples are generally ~3 to 5 cm thick, with angle of climb increasing upward and ranging from ~10° to 48°. The climbing ripples and medium-grained sands of 1b grade into the fine sand/coarse silt-dominated subdivision 1c (**Figs. S5, 6**), which is ~18 to 25 cm thick. While lowermost 1c is dominated by interlaminated fine sand and coarse silt, this upwardly grades to a fine silt-dominated composition in uppermost 1c. In basal 1c, climbing ripples are preserved in ~2 to 4 cm thick sets, with angle of climb upwardly increasing from ~40° to greater than 50° where gentle sinusoidal laminations are preserved in ~2 to 4 cm-thick sets, before transitioning to fainter low-angle cross-lamination and fine discontinuous horizontal lamination in uppermost 1c. Flame structures are associated with loading of an upper sand/coarse silt layer into very soft silt in mid-lower 1c (**Fig. S8**), and point obliquely in an inland (westward) and up-hill direction compatible with the aforementioned flow indicators. Most of the flame structures (ranging from 0.5 to 4 cm high) are composed of fine silt, however some are composed of mats of vegetation and plant debris rather than sediment (e.g. **Fig. S8 C, D**). Middle-upper 1b and lower-middle 1c are noteworthy in being characterized by abundant mm- to cm-scale alternations of light-colored sand/silt with dark grey medium-fine silt. Overlying 1c is a dark, well-defined, relatively continuous ~3 to 5 cm-thick band of very fine silt/mud (1d), which is nearly structureless but does contain some faint indications of ripples and bedding traces. The contact between this band and Unit 2a immediately above it is very sharp, in some areas slightly scoured, and not gradational.

Unit 2 of the Event-Deposit. Unit 2 is ~80 cm thick, directly overlies the dark mud band of Unit 1d, and is an overall fining-upward sequence beginning with a sand-silt-dominated basal portion and grading to a fine silt-mud-dominated upper terminus. The color grades from buff-tan in the lower portions to dark grey in the upper portions, corresponding to the proportion of interlaminated sand or fine-grained silty sediment. Unit 2 is thicker than Unit 1, and more silt-dominated. Unit

2 lacks a basal conglomeratic sand as seen in Unit 1, but otherwise is a mirror of middle-upper Unit 1 both sedimentologically and structurally. Flow indicators such as dip direction of cross-bed foresets and climbing ripples indicate a preferred paleocurrent direction of ~N 63-67 W for Unit 2a and most of 2b, and nearly 180° opposite for Unit 2c (~S 60-65 E). This is in agreement with flow indicators from vertebrate and invertebrate biological debris (see below). Basal Unit 2 (2a) is ~30 to 35 cm thick and is a near-replicate of Unit 1c, grading upward from climbing-ripples with upwardly increasing angle of climb, to sinusoidal wavy lamination, and fine, low-angle discontinuous cross-lamination. The alternating sand and fine silt laminae in lower 2a (identical to those in 1b-c) are sand-dominated, while the laminae in the upper portions of 2a are more silt-dominated, with far less sand content (**Fig. S6**). Climbing ripple sets are equivalent in dimensions and angle of climb to those of Unit 1, and are most-common in the basal half of 2a. The succeeding subunit, 2b, is ~30 to 35 cm thick. The transition from Unit 2a to 2b was selected as the point at which sand is no longer present in the laminae, which in 2b are comprised only of alternating fine and coarse silt. Bedding traces become faint and difficult to discern in upper 2b, where grain size is predominantly medium to fine silt. Unit 2c, ~15 to 20 cm thick, is distinguished by its near-absence of any bedding traces or laminae, and very fine silt composition, which grades upward into a dark, organic-rich very fine silt/mud with abundant mats of plant material at the top. The uppermost sediment of 2c is dark, organic-rich, flaky, and highly friable (**Fig. S6**). This is directly overlain by a 1 to 2-cm-thick, in-situ band of smectitic tonstein that is enriched with ejecta spherules, shocked minerals, iridium, and other impact-derived materials (**Figs. 3, S1**).

Interpretive sedimentology. Whereas the entire Event-Deposit sediment column shows overall decrease in grain size from base to top (**Fig. S4**), constituent Units 1 and 2 record two immediately successive pulses in flow energy, each of them normally graded and individually showing clear signs of waning flow (drop in grain size, transition from upper- to lower-flow regime structures, etc.). Virtually all of Unit 1 (1a-c) records the landward (northwestward-directed) inundation phase, opposite the normal paleocurrent of the underlying river demonstrated by the point-bar, while the uppermost fine-grained band (1d) records a backwash phase directed 180° seaward (southeast). Lower Unit 1 (1a and lowermost 1b) is characterized by nearly structureless massive sand with rip-up clasts, and some discontinuous plane-parallel bedding (lowermost 1b), representing upper flow regime conditions. Truncated flame structures in 1b are noteworthy in that they are indicators of rapid sedimentation (***8***), and are common in tsunami-like inundations, however are not as frequent in river or storm deposits (***9***). The ones observed at Tanis are nearly identical to examples from modern tsunami inundation deposits (***9***), and, as with

the modern examples, give an indication of flow-direction. The climbing ripples, wavy lamination, low-angle cross-lamination, and discontinuous horizontal lamination of upper Unit 1 (1b-c) represent waning flow during lower flow regime conditions (***8***). The fine silt/mud of Unit 1d, which contains southeastward flow indicators recorded by very faint and uncommon bedding traces/laminae, represents lowermost flow regime conditions and was laid down during waning flow and outwash of the first surge pulse. The conspicuous mm- to cm-thick alternations of thin white sand laminations with dark silt layers in 1b-c (**Figs. S5, 6**), were caused by micro-variations of flow during deposition and are commonplace in onshore-deposited inundation deposits (e.g. ***10-12*****; Fig. S9**).

Unit 2, a graded bed comprised of medium/fine sand to fine silt/mud, was laid down by waning flow, largely under low flow regime conditions, and represents a second inland-directed surge pulse that immediately succeeded Unit 1. Unit 2a begins with climbing ripples, wavy laminations, and cross-lamination, and lacks a basal massive sand deposited under upper flow regime conditions (i.e. Unit 1a), and thus this second surge carried less overall energy than the first. Most of Unit 2 (2a, most of 2b) records inland-directed flow, while the upper portion (primarily 2c) records outwash in a seaward (southeast) direction. The highly fissile fine-grained siltstone and mudstone, with no obvious bedding traces and containing abundant mats of fragmented plant debris at the upper terminus of 2c, is a common feature following cessation of an abrupt, short-lived inundation event (***11***).

Evidence for rapid and abrupt deposition of the Event-Deposit. The two surge packages of the Event-Deposit were deposited in rapid succession by an abrupt event for a series of independent reasons: 1) there is no evidence of a considerable hiatus between them such as mud cracks, root traces, or burrows/other bioturbation, 2) delicate biological debris (including articulated vertebrate carcasses and branchs with attached leaves) transcends from Unit 1 up into Unit 2, none of which would have remained unmolested during a hiatus, 3) ejecta of identical morphology and geochemical composition occurs in both Units 1 and 2, but not in the bounding strata, 4) the two units bear nearly identical lithology and structure that represent rapid sedimentation out of suspension, and 5) the deposit directly overlies a paleosurface that did not frequently experience subaqueous sedimentation, and was subaerially exposed for an extended time before inundation. We therefore conclude that the Event-Deposit was abruptly emplaced upon a subaerially exposed paleosurface, and that the two sub-units were deposited in rapid succession as elaborated upon below:

Sedimentology indicates that the Event-Deposit was rapidly emplaced during an episodic event. The structure and grain size profile of the Event-Deposit sediment package bears indications of rapid sedimentation out of suspension, which additionally are nearly indistinguishable from modern inundation surges (***11, 13***). These include 1) climbing ripples; 2) normal grading from coarse to fine-grained sediment; and 3) a steady sequential transition from high-energy upper flow regime deposition to low-energy, low flow regime. The climbing ripples are clear indications of deposition out of suspension by a rapidly waning flow (***8, 14***). The rate of deposition (= rate of energy decay) increases upward, as indicated by the steepening climb of the ripples from sub-critical to super-critical, and then sinuous ripples (***8***). Graded units with climbing ripples similar to those at Tanis are typically deposited quickly, on the order of ~tens of minutes to an hour (***8, 15***), indicating that the Event-Deposit did not take long to form. This rate of deposition is in agreement with the duration of modern onshore inundations, in which individual surge pulses last 10's of minutes and the entire inundation concludes which within one to several hours (***13***). This rapid deposition is supported by additional features, such as truncated flame structures and de-watering structures, both of which are associated with rapid deposition of copious sediment. The ejecta in the deposit further demonstrates the rapidity of deposition. As ejecta is present throughout the sediment column of the Event-Deposit, it is clear that its deposition occurred after impact. However, because the deposit it capped by KPg tonstein, comprised of microscopic debris from the impact, we deduce that deposition of the Event-Deposit occurred very shortly after impact before the tonstein had a chance to settle out of the atmosphere. Stratigraphic placement of the Event-Deposit directly atop a paleosurface that was exposed for an extended time, was bioturbated, and in some places underwent pedogenesis, indicates an abrupt depositional event in a location where such deposition did not frequently take place.

Chicxulub tonstein, and distribution of ejecta within the Event-Deposit. The tonstein at Tanis is typical of the KPg boundary elsewhere in the Western Interior (***16***), which manifests in a dual-layered microstratigraphy characterized by impact ejecta spherules in the lower layer (**Tables S3, 4**), and shocked minerals, plus anomalous levels of iridium above normal background concentration (typically below detection limits), in the upper layer. We interpret the tonstein overlying the Event-Deposit to primarily represent the upper layer. Ejecta spherules from the contact between the tonstein and Event-Deposit are sparse, but identical to isolated examples from throughout the Event-Deposit (**Fig. S10**), and compare favorably with specimens isolated from samples from Mud Buttes provided by Jeffrey Person, NDGS. Primarily preserved as clay-altered glass, a few (very rarely) contain unaltered fragments of glass at their cores (**Fig. S10 B**), not previously reported in North America. Micro-crystalline, nickel-iron-rich microkrystites have been recovered from this layer as well (**Fig. S11**),

also hitherto not reported from North America. Near the top, we observed a concentration of shocked minerals (**Fig. S11**), plus an iridium anomaly of 3.8 ppb (**Fig. 2, Table S5**). This layer represents the settling of fine impact-generated debris which initiated shortly after impact and continued to settle during the ensuing days to months.

A thin ~6 cm carbonaceous bed overlying the KPg tonstein (**Figs. 3, S1**) revealed an impoverished palynofacies dominated by ferns (>67%), comprised mostly of *Cyathidites* and *Laevigatosporites*, with minor *Azolla* and *Deltoidospora*. This correlates with the well-documented "fern-spike" that occurs in strata immediately succeeding the impact layer (***16***). The succession of the KPg tonstein; an organic-rich lignitic stratum preserving the fern-spike; and an overlying basal Paleogene sandstone body is a classic terrestrial manifestation of the KPg boundary as preserved throughout the Western Interior and directly correlates with the stratigraphy of nearby KPg sections such as Mud Buttes.

Ejecta spherules, impact-melt glass. Ejecta spherules exhibit a diverse variety of splash-form shapes such as elongated ovoid-, teardrop-, dumbbell-, and globular-shaped spherules (**Fig. S10**). Some lumpy/irregularly shaped spherules have a rough surface from devitrification/dissolution, are highly vesicular, and are very scarce. Because they are so rare, larger than average size, and prone to mechanical flattening while altered to smectite, they can be easily mistaken for mud clasts unless the internal structure is examined. Therefore, it is reasonable that they have gone unnoticed if present at other Hell Creek and Western Interior KPg localities. The vast majority of spherules are smaller, and exhibit glassy or dull surface textures. Some have well-defined wavy schlieren patterns formed during ballistic travel. All such spherules began as fluid impact-melt glass that solidified in space, and have since been diagenetically altered to clay minerals after burial (***17-20***).

Spherules are scattered throughout the Event-Deposit, and display an overall fining-upward trend in average diameter from base to upper bounding surface of the deposit (**Fig. 2**). Rip-up clasts in lower Unit 1 are all much larger than the spherules and hence the spherules there were well within transport capacity, almost certainly syn-depositionally reworked. Spherules scattered throughout upper Unit 1 and all of Unit 2, typically as isolated spherules but occasionally in small graded lenses, averaged 0.2 to 1 mm in diameter, with average diameter decreasing from base to top of the deposit. These spherules are all much larger than contextual grain size (= carrying capacity), and therefore they likely settled to the bottom after arriving via primary air-fall. It is most probable that the spherules entered the flowing surge as atmospheric fallout, were transported as bedload for a short period, and then were deposited shortly thereafter, within tens of minutes after

entering the water. While the majority of spherules (~0.3-1.4 mm) are within the size range expected relative to their distance to the crater, the much smaller proportion that are larger (2+ mm) are unusual outliers. While we do not here speculate on how they relate to the dynamics of the impact event, we do make a comment on how they relate to nearby KPg outcrops. Our observation of local KPg boundary outcrops near Tanis revealed that diagenetically altered smectitic spherules can frequently weld to the interstitial smectite of the tonstein, making them difficult to see and perhaps leading to them being under-recorded. A detailed and systematic excavation, as we conducted at the Tanis deposit, is rarely undertaken. Given the rarity of large spherules at Tanis, such a systematic excavation might be the only reliable way to search for them in the tonstein elsewhere. In support of this, cursory examination by our team of a KPg cross-section north of Tanis at Brooke Butte revealed low spherule abundance and no sizes larger than 1 mm, however a systematic excavation by shaving down the tonstein covering ~54 m^2 of the same outcrop revealed a much higher abundance of spherules, including a handful (6) of larger spherules comparable to the ones associated with down-warped “funnels” at Tanis.

Cone-shaped disturbances of down-warped sediment punctured the dark alternating mud-silt laminae of Unit 1c and d. In cross-section, they appear as inverted funnel-shaped cones filled with disturbed, unstructured sediment (**Fig. S12**). Sediment laminae are prominently bent downward near the walls, and a single large clay-altered ejecta spherule is found at the bottom of many of these cones. These structures are rare (fewer than 20 have been found), and always occur within the same general stratigraphic horizon in uppermost Unit 1c, d. The association with a single spherule demonstrates that the direct fall of the spherule has caused them, and hence they represent strong evidence for primary air-fall ejecta accretion. Normal horizontal sedimentation resumes immediately above, indicating that the punctures were formed during accumulation of the Event-Deposit. Preliminary experimental investigation in which a glass sphere was embedded at terminal velocity in sediment that had been sieved from Tanis and laminated to replicate the structure of the Event-Deposit, produced identical cone-shaped structures with warped edges (**Fig. S12 C**), indicating that ejecta spherules were physically capable of causing the structures, but also not ruling out other causes such as water escape structures. However, water escape would still not explain the association of single impact-melt spherules with nearly all of the cone-shaped depressions, or the down-warped edges.

Unaltered glassy ejecta spherules were additionally recovered from amber attached to trees that were clearly alive or recently felled at the time of deposition, as indicated by delicate in-situ coniferous needles and bent, unbroken thin distal

branch tips that were still pliable at the time of deposition (**Fig. S14**). Starting with the first systematic excavations in 2012, diagenetically altered ejecta spherules have been regularly observed (and subsequently noted by unrelated individuals/students) wedged between the gill rakers of acipenseriform fish (**Fig. S15**). The surrounding matrix and the other body regions within the fish have comparatively very few spherules. It is unlikely that these spherules were collected after death of the fishes, but rather that they were collected from the water column while the fish were free-swimming. It is thus-far unclear how prevalent this spherule-enrichment is among the fish assemblage, as fewer than 20 have as-yet been checked, however it is a clear indication that the fish were alive immediately prior to deposition.

Ejecta geochemistry, and connection with the Chicxulub Impact. The alteration phase of diagenetically altered Tanis spherules is semi-translucent dark tan-brown in color, sometimes with greenish hues. It is generally Si—Al—Mg—Fe-rich respective to the other major oxides. Spherules from the tonstein capping Tanis, within the Event-Deposit, Mud Buttes, and other distant KPg localities are all similar in absolute and proportionate trace element values (**Table S3**), and overall composition is similar to alteration products reported from El Mimbral, Beloc, and other localities (***18, 20-23;*** **Fig. S16**). Alteration phases hold little value for comparative purposes, however, due to different diagenetic histories from widely spaced geographic regions.

Unaltered glass cores from the interior of diagenetically altered spherules is dark brown-black in color, vesicular, and has a heavily scalloped surface morphology (**Figs. 3, 5, S10**), similar in appearance to examples recovered from El Mimbral Mexico, and Beloc Haiti (***17***). Three tiny fragments of unaltered glass were also found in the Mud Buttes sample, not previously reported from that site. The glass is characterized by high SiO_2 (63.82-66.03%) and Al_2O_3 (15.06-15.96%), and low MgO (2.43-2.75%), FeO (4.41-4.83%), and CaO (5.14-5.93%), broadly reminiscent of all reported unaltered KPg glass, but specifically indistinguishable from compositional ranges exhibited by Chicxulub black glass (***17, 24, 25***; **Table S4**). Chondrite-normalized trace element analysis revealed a marked similarity with the ranges exhibited by Chicxulub black impact glass (**Fig. 5**), and binary variation diagrams reinforce the close affinity (**Figs. S17-19)**. The refractive index of Tanis glass is low, ranging from 1.53-1.55 for glass from the capping tonstein and 1.52-1.54 for glass from within the Event-Deposit, comparing favorably with impact-melt glass and falling within the 1.51-1.55 ranges reported for Chicxulub glass from elsewhere (***26***; **Table S5**). The low refractive index and very low water content (0.02% to 0.03%) are indicative of

impact, and exclude a volcanic origin. $^{40}Ar/^{39}Ar$ dating of the glass is in agreement with other dates established for the KPg boundary (***27-29***) producing an age of ~65.76 Ma +/-0.15 Ma (**Table S2**), following the calibration of Kuiper (***27***).

Ejecta spherules collected from amber runnels on buried tree trunks at Tanis (**Fig. S13 B**) had identical composition as the unaltered shards of black glass (**Table S4**). Optical microscopy and SEM of thin sections revealed a smooth, amorphous internal structure for the glassy spherules (**Fig. S14**), which are commonly vesicular, and with overall morphology nearly identical to the diagenetically altered clay examples (**Figs. S10, 13**). Internal structure is nearly homogenous, with faint schlieren and occasional angular debris (**Fig. 5**), in addition to clusters of melilite crystals near the spherule rims, similar to unaltered examples from Gorgonilla (***24***), and reminiscent of relict crystallites seen in clay-altered spherules (**Fig. S14**). Geochemical and trace element composition of the glass, water content, and refractive index are identical to the other unaltered glass fragments from the site, confirming a genetic link to the other Chicxulub ejecta.

Correlation with the KPg boundary. The KPg tonstein that conformably caps the ejecta-bearing Event-Deposit is a pristine in-situ horizon of impact-related debris generated by a well-documented impact event (see above). As such, the tonstein, itself, provides a strong isochronous stratigraphic benchmark (***30***), integrating Tanis within both a local and global stratigraphic framework and correlating the depositional event with the Chicxulub impact. Identical ejecta in the Event-Deposit and tonstein unites those two lithological units with the same impact event, and correlates them with the KPg boundary.

Biostratigraphy strengthens correlation with the KPg boundary by demonstrating that the point-bar and overlying Event-Deposit bear an uppermost, terminal-Cretaceous signature, immediately preceding the Paleogene. Palynostratigraphy is among the most useful and commonly employed biostratigraphic tools to define the KPg boundary and bounding strata, and is most-robust in conjunction with other methods rather than solo (***16***). We supplemented our palynological data with megafloral calibration, which corroborated each other neatly. Megafloral biostratigraphy indicates that the incised bedrock occupies the mid- to lower-HCIII megafloral zone (***31***), which correlates with the bedrock's actual stratigraphic elevation with respect to nearby Hell Creek outcrops (**Fig. S20**). Palynostratigraphy (**Fig. S21, Table S6**) corroborates this assessment, placing the bedrock within the "D" subzone of the *Wodehouseia spinata* assemblage, which occupies the upper, but not terminal, Hell Creek (typically spanning the upper ~5 to 28 meters of the Formation)

sensu Nichols (***32***), his figure 5. The incised point-bar sands, and the Event-Deposit, bear a much younger signature than the incised bedrock that they superpose. This couplet is allied with the terminal-Cretaceous "E" subzone of the *Wodehouseia spinata* assemblage **(Fig. S21)**, which occupies the uppermost ~0 to 5 meters of the Hell Creek Formation and incorporates the KPg boundary (***32***). Megafloral biostratigraphy is in agreement with this, placing the couplet within either the FU0 or uppermost HCIII megafloral zones, both of which are constrained to the uppermost, terminal-Cretaceous, up to and including the KPg boundary (***31***). The Event-Deposit and point-bar sands are therefore much younger than implied by their lower elevation respective to the nearby KPg outcrops that are preserved in floodplain and overbank facies, which is expected in an incised valley depositional setting (*7*).

The base of the Danian *sensu-strictu* is defined as either 1) "the horizon equivalent to the moment of impact", or "the base of the millimeter-thick airfall unit" (***33***). As the Tanis depositional event contains ejecta, the base of the Danian should be placed at the onset of the Event-Deposit, identifying the site as basal-most Paleogene in age. This chronology, immediately following the close of the Cretaceous, would be too rapid to reflect the palynological and megafloral shift between the Cretaceous and Paleogene, which would have taken years or millennia. Therefore, terminal-Cretaceous palynological and megafloral signatures would be expected for an abrupt, instantaneous event that occurred precisely at the Cretaceous-Paleogene transition. Thus, the biostratigraphy at Tanis is in agreement with it immediately following the Chicxulub event, and the combination of 1) the KPg boundary tonstein, 2) megafloral biostratigraphy, and 3) palynostratigraphy, each independently unite Tanis with a narrow temporal window at the basalmost Paleogene.

Biota in the Tanis deposit, and taphonomic observations

The anomalously high local abundance of tightly packed vertebrate carcasses and flora at Tanis preserves the first Konservat-Lagerstätte known from the Hell Creek Formation (**Figs. S22-24**). This vouches for an uncommon taphonomic and depositional mode at the site, because Konservat-Lagerstätten, rare in the fossil record, require special depositional circumstances to preserve them. Nearly all of the vertebrate remains are comprised of articulated, nearly perfect carcasses, many with three-dimensional preservation. Biota in the Event-Deposit 1) indicates flow direction of paleocurrent, 2) indicates contribution of debris from marine facies, and 3) bears the taphonomic signature of a catastrophic assemblage (death-assemblage).

Marine indicators. Marine microfossils consist primarily of dinoflagellate cysts, which were recovered throughout the Event-Deposit (**Table S6, Figs. S25**) but are completely absent from the upper and lower bounding strata. There is an inverse relationship between grain size (= system energy) and palynomorph recovery, with greater abundance and preservation with decreasing grain size. Greatest abundance and preservation is generally in the uppermost, fine-grained siltstones, and poorer elsewhere in the deposit. Similar variation of abundance and completeness is common in modern and fossil inundation deposits (***12, 34-37***). Recovery is also much poorer in sediment taken from desiccated previously excavated blocks kept in long-term storage or debris matrix generated during fossil preparation than from the fresh outcrop, probably owing to the fragility of the thin-walled dinocysts. Because of the inconsistency in abundance and condition of dinocysts in old/debris matrix, and the chance for contamination, we caution the unreliability in its utility. The dinoflagellate cysts (***38, 39, 40-47***) fall into three categories: markers for the latest-Maastrichtian (= contemporaneous with the deposit); taxa with wide temporal ranges throughout the Maastrichtian (= compatible as contemporaneous), and uppermost Cretaceous taxa that went extinct before the latest-Maastrichtian (= reworked older material). No definitive markers for the Danian were recovered. This mixed representation is compatible with a large, turbulent event, which would have disturbed the bottom sediments and produced a palynological mixture just as we see at Tanis, and is consistent with observations of modern inundation deposits (***12, 34-37***). A similar amalgamation of reworked and contemporaneous skeletal debris also characterizes nearly all turbulently deposited sediments at the KPg boundary level which have previously been attributed to seiche or tsunami activity, particularly along the Gulf Coastal Plains (***33, 35***).

Marine invertebrate macrofossils are dominated by very well preserved, mostly nacreous aragonitic fragments and partial shells of ammonites. Thus far, the material consists almost exclusively of *Sphenodiscus lobatus* (**Figs. S3, 22**), but also several small fragments of c.f. *S. pleurisepta*, which spans from the uppermost lower Maastrichtian *Baculites clinolobatus* zone to the uppermost upper Maastrichtian *Hoploscaphites nebrascensis* zone of the WIS (potentially also extending to the terminal-Cretaceous WIS, however that is unclear since the terminal portion is missing) and also in terminal-Maastrichtian assemblages elsewhere around the Gulf coastal plains (***48***). Most ammonite fossils are preserved as extremely delicate, thin (0.7 to 1.2 mm thick), small (4 mm to 2 cm) angular fragments, however larger (~10 cm) fragments and nearly complete shells of *S. lobatus* have also been recovered. While the high degree of breakage suggests that the

majority if not all of the recovered ammonite fragments were from prior-dead animals, many fragments are angular, with little to no obvious signs of abrasion, suggesting that they are from recently dead organisms rather than extensively churned or reworked. And while nacre is among the strongest component of marine shells, large, thin shells such as those of *Sphenodiscus* require little stress to fracture, and readily disintegrate when subjected to even minimal hydraulic stresses (***49***). Such breakage could make a recently dead assemblage appear older or reworked. In all instances at Tanis, the ammonite shell fragments are scattered and disbursed in the deposit, and do not occur in discrete lenses. The ammonite shell fragments possess isotopic signatures consistent with a marine habitat, ranging from -0.61‰ to -1.5‰ δ^{18}O VPDB. Teeth from marine vertebrates found at the site (fish, reptilian), ranged from -2.6‰ -6.88‰ δ^{18}O VPDB, compatible with fully marine to nearshore/brackish water values (**Fig. S3**). By comparison, freshwater fish had δ^{18}O VPDB values of -9.0‰ and lighter, confirming their freshwater provenance.

The source of the marine fossils at Tanis is thus-far unknown, although marine indicators in the uppermost Hell Creek Formation are peppered throughout the region, ranging from slightly west of Tanis (near Montana-North Dakota border) to over 250 km eastward, indicating that influence from the WIS (such as tidally influenced zones, a marine arm or embayment, or estuary) could have been very close to the Tanis region (***2, 50-54***), perhaps as close as several km. The marine fossils are primarily small and/or have large surface area, such as the very thin ammonite shell fragments, and consequently they represent the easiest-transported by water flow. This is supported by the lack of thick, bulky marine fossils such as oysters or other robust elements. The marine taxa could be derived from older strata exposed during the terminal-Cretaceous, or from living stock in the WIS, including shell-debris from recently dead organisms lying on the coastline or seafloor. Such a mixed provenance is also in keeping with assemblages seen in modern inundation deposits (***12, 34-37, 49***). Upstream sources for late-Maastrichtian marine fossils during the terminal Cretaceous (i.e. contemporaneous erosion down into the Fox Hills and older Pierre Shale) have not been reported, so there was unlikely a contribution of marine fossils via normal fluvial transport. They were almost certainly brought in from a source to the East, via the inland-directed flow surges. Ammonites could have been reworked from the older off-shore Pierre shales or from near-shore to brackish Breien and Cantapeta deposits and brought in via the inland-directed surge pulses, however the latter two deposits contain a wholly different set of marine fossils and completely lack *Sphenodiscus*. The Breien and Fox Hills are also stratigraphically much

lower than Tanis, making erosional exposure to that depth difficult and highly unlikely. The soft WIS bottom, east and southeast of Tanis, was the most-likely contemporaneous source for reworked marine fossils, and deposits from the Cantapeta or an as-yet undocumented marine incursion could have been a minimal secondary source (if even a source at all).

Terrestrial fossils. Preservation of plant material supports a rapid, abrupt depositional event, and also supports the inferred flow direction. Aside from very large tree trunks or branches that rest on the base of the deposit and cross-cut the entire Event-Deposit thickness, the majority of plant material occurs in finer sediments starting immediately above the densest accumulation of carcasses, between 4.8 and 40 μm grain size, and is commonly associated with sinuous, wavy ripples, low-angle cross-lamination, and discontinuous fine parallel lamination. Woody material is often preserved as partially compressed, three-dimensional fossils that preserve vascular microstructure throughout their interiors (**Fig. S22 D**), differing from the typical mode of soft-sediment plant preservation in the Hell Creek Formation, which consists of lignification or impressions that lack depth or internal structure. The presence of leaves or needles on many of the branches, and the completeness of the individual leaves (which lack signs of prior decay such as leaf skeletonization, maceration etc.; ***55***) suggests very little transport. In addition, such completeness of the plants suggests that they were alive or recently felled at the time of deposition, and were not already dead and decaying. Highly angular fragments of burnt wood (**Fig. S24 E**), and burnt patches on large tree trunks, are abundant throughout the deposit. When the wood was burnt, or if the burning was contemporaneous with the depositional event, is uncertain. There is no obvious pattern of charcoal distribution throughout the sediment column, and there would almost certainly have been some degree of syn-depositional reworking. Elongated plant material such as branches, twigs, and logs, are primarily oriented along a southeast/northwest azimuth (~S 65 E to S 55 E; **Fig. S26**), with some less-frequently oriented roughly 90° to that azimuth, a pattern of orientation associated with transport by water flow (***56***). Some trunks and large branches cross-cut the entire Event-Deposit at an oblique angle through the sediment column (**Fig. S27**). Pliable fronds and carcasses are sometimes seen snagged by other debris and bent in the direction of flow (**Fig. S7**). The flow direction indicated by the plant material is in agreement with the orientation of carcasses (see below) and sedimentary structures (see above).

Articulated, well-preserved vertebrate carcasses are highly concentrated at Tanis and occur throughout the stratigraphic thickness of the Event-Deposit, however the zone of densest concentration vertically spans from uppermost Unit 1c to lower Unit 2a (**Figs. 2, S6, 23**). The most abundant (hundreds or more) vertebrates at Tanis consist of complete,

partially inflated carcasses of paddlefish and sturgeon taxa (Acipenseridae, Polyodontidae). Concentration of vertebrate carcasses is typically greatest in grain sizes between 55 and 74 μm, and commonly associated with strata exhibiting supercritical climbing ripples (15°-45° climb) or sinusoidal ripples (>45° climb). Preservation and completeness do not markedly vary and are universally excellent. Of the specimens that exhibit articulation, nearly all are perfectly preserved. Most carcasses are at least partially three-dimensional, and extremities or appendages are often oriented obliquely in the sediment column, not constrained to any single horizontal plane of lamination. The carcasses are fully articulated and there are no signs of decay or bloating prior to deposition, such as disorderly/disrupted skeletal articulation (particularly in the abdominal region), disarticulated or blown-out skulls, deformation of the bodies, etc. Jaws are preserved in a widely gaped, open position in more than 80% of the fish carcasses. In a similar proportion of individuals, the fins are highly flabellate (fanned out), preserved in a maximally extended posture. In all of these specimens, the individual fin rays are orderly, equally spaced, and well-preserved. Most fish carcasses remain straight along their body axis, however some are slightly arched or curved along the spine.

The carcasses at Tanis are strongly flow-oriented, particularly evident in the elongated bodies of fish (**Figs. S26, 28**). Except for carcasses that are twisted and tangled against large branches or tree trunks, causing scatter in the plotted orientation, the majority of carcasses are oriented along a southeast/northwest azimuth (**Figs. S23, 26, 28**), with their heads indicating the direction of flow (***57***). Some of the carcasses were less-frequently oriented ~90° to the preferred azimuth (= flow direction), a feature well documented during current-driven transport of organic debris (***56***). Flow direction indicated by overall carcass orientation is concordant with indications given by plants and sedimentary structures (**Figs. S7, 26**; see above).

In addition to articulated carcasses from fresh-dead individuals, several examples exist of isolated or small clusters of bones with minimal adhered soft tissue. These specimens were clearly already dead at the time of deposition, yet still shortly enough after death that they retained some traces of intact tissue. One example of this taphonomic mode is a partial ceratopsian ilium with associated impressions of tissue, within Unit 2 of the Event-Deposit (**Fig. S29**). While slightly higher upslope on the point-bar than the densest carcass assemblage, the specimen occurred within the same stratigraphic horizon of the Event-Deposit (**Fig. S29**) and was associated with several articulated fish carcasses. In modern analogs, completely

skeletonized bones/bone clusters that retain sizable portions of sun-hardened tissue can be readily transported by water flow (**Fig. S29**). We infer a similar taphonomic history for the ilium at Tanis. The isolated, extensively transported bones, and uncommon isolated bones with associated soft tissue (e.g. ilium) were certainly dead prior to deposition of the Event-Deposit and therefore are not part of the mass death assemblage. The articulated, well-preserved carcasses at Tanis, however, are supported as a single mass-death assemblage by multiple taphonomic factors, described below.

Features supporting a mass-death assemblage. The carcasses at Tanis represent a mass-death assemblage based on 1) universally excellent condition, with prevalent three-dimensional preservation, 2) shared mode of death, and 3) a "catastrophic" distribution of body sizes that more closely resembles that of a standing population than an attritional assemblage. The completeness and excellent preservation of the vertebrate carcasses, such as intact body envelopes, fully preserved skeletons, nearly pristine fins, the lack of displaced/disarticulated skeletal elements, and prevalent three-dimensionality of carcasses, is indicative of rapid burial while alive or immediately after death. The uncommonly good preservation indicates that the carcasses were fresh at the time of deposition, likely owing to rapid burial (***58***), and the near-identical state of preservation between individuals suggests that they share similar taphonomic histories. This contrasts with depositional horizons that contain widely differing preservation and taphonomic histories among individuals, such as the Green River Formation, which contains a time-averaged, attritional assemblage (***59***). The depositional scenario at Tanis (i.e. rapid deposition by a single, abrupt event) offers further support that the articulated carcasses represent a death assemblage. Deposition by a single event reduces the likelihood for prolonged, gradual accumulation of articulated individuals over time (i.e. within a lacustrine deposit). Together, this evidence of shared/similar taphonomic history and universal exceptional state of preservation indicate that 1) the individuals died at a similar point in time, and 2) that death occurred soon before, or at, their time of burial.

The carcasses at Tanis also share a similar mode of death. Taphonomy of the fish elucidates clear signs of tetany, a condition that signifies rapid, sudden death due to 1) poisoning, 2) asphyxiation, or 3) physical choking up or blockage of the gills (***57, 60-62***). Tetany in fish manifests as 1) extremely flabellate (open in fan-like fashion) fins which are fully or nearly fully deployed, 2) a widely gaped, open mouth, and 3) a slight warp or bend to the main axis of the body (***57, 63***). The signs of tetany are easily distinguished from the effects of normal decay or bloating based on the overall condition of the carcass.

For example, opening of the mouth or splaying of the fins resulting from decay would also be accompanied by clear signs of degradation in the rest of the carcass, such as disarticulation or shifting of skull bones, displacement of other skeletal elements, or severe degradation of the body wall and other soft parts, especially fins, which are among the first parts to exhibit signs of decay (***64***). The fish carcasses at Tanis show exceptional preservation with no signs of decay or degradation, and the majority display clear signs of tetany, including widely splayed flabellate fins, widely gaping mouths, and to a lesser degree, slightly arched bodies (**Fig. S30**). Fish represent the most plentiful type of vertebrate at Tanis, and therefore their probable unified cause of death can justifiably be extended to the full remainder of the Tanis carcasses that exhibit an equivalent degree of uncommonly high preservation and completeness.

The rarity of acipenseriform fish in the Hell Creek Formation further argues against a gradually accumulated attritional deposit of carcasses. An attritional deposit of acepenseriforms does not exist anywhere else in the Hell Creek, owing to the fact that 1) they decay and disarticulate rapidly after death, and 2) there is a paucity of depositional environments conducive to their articulated preservation. Fewer than 5 articulated acipenseriforms had been reported from the Hell Creek prior to this study, and none were preserved in a depositional scenario comparable to Tanis (***3***). The previous Hell Creek specimens were preserved in the body cavities of hadrosaur skeletons, which provided a quiescent, shielded micro-depositional environment (***3***), contrary to the typical depositional environments within the Hell Creek Formation, none of which are conducive to preservation of an attritional fish assemblage. Due to their fragile anatomy and near-lack of an osseous skeleton, acipenseriforms disintegrate rapidly after death, particularly in a submerged aqueous environment, as with most fish (***56***). Attritional deposits of articulated acipenseriforms are rare outside of a quiescent lacustrine environment (***59***), and are understandably absent from the moderate- to high-energy Hell Creek river systems. Horizons of densely packed fish carcasses are extremely rare in any fluvial sequence (***57***), thus they would not be expected as an attritional deposit at Tanis under normal fluvial conditions. Taking into account the rarity of depositional environments conducive to preserving an attritional assemblage of acipenseriform fish, and the rarity of finding a single articulated fish in the Hell Creek Formation, it is unlikely that the Tanis afipenseriforms represent a long-accumulated, attritional assemblage.

Frequency of body sizes, which can reliably distinguish between a catastrophic assembage (= death-assemblage) or attritional assemblage, indicates that a genuine death assemblage is present at Tanis. Catastrophic assemblages, whether

modern or fossil, more closely resemble a standing population (***56, 65***), and graphically appear as a steadily decreasing frequency from small to large body size (***65***). Attritional deposits, on the other hand, have an abundance of small and large body sizes (= young and old individuals), and a drop in intermediate stages, resulting in bulges at both ends of the frequency graph. At Tanis, the body size distribution for the acipenseriform fish, which comprise the majority of carcasses at the site, clearly exhibits a catastrophic rather than attritional signature (**Fig. S31**). Considering that the largest proportion of carcasses (Acipenseriformes) exhibits indication of catastrophic assemblage, we extend this inference to the remainder of the equally well-preserved carcasses that comprise the Tanis death assemblage.

Depositional mechanism

In addition to the sedimentology at Tanis being incompatible with the normal fluvial processes in the Hell Creek Formation (i.e. river flood or storm), and differing from circum-coastal sedimentation such as tidalites, the Tanis deposit is highlighted as an unusual occurrence by containing a mass-accumulation of fish, (exceptionally rare in any fluvial setting [***57***]) and by containing the only known Konservat-Lagerstätte in the Hell Creek Formation. Together, these features suggest that a depositional mechanism other than normal/common Hell Creek fluvial activity produced the Tanis deposit.

Tsunami or seiches have been proposed as the cause of many high-energy KPg sediments found in marine facies (***33, 35, 66, 67***). Whereas some sedimentological aspects of tsunami deposits found in marine facies (i.e. rapid deposition out of suspension, contorted or loaded/deformed bedding, graded units with basal rip-up clasts, etc.) could superficially resemble other submarine phenomena (such as turbidity flows, shelf collapse, etc.), there are no large-scale turbulent processes in continental facies that could sedimentologically mimic tsunami or seiches. Thus, interpreting sedimentation from an onshore inundation event (i.e. Tanis) is much more straightforward than in marine facies.

Submarine slumping is capable of displacing large volumes of water, and numerous major slumping events, some massive enough to result in contorted, turbulent submarine bedding over 5 meters thick, are preserved throughout the WIS of Colorado, Wyoming, and South Dakota during the latter half of the Cretaceous (***54***). Such movement could have caused large tsunami, based on historical analog, however no onshore inundation deposits have thus-far been attributed to those episodes

of submarine slumping. Therefore, there is no precedent or basis for comparison with Tanis, and speculation on this mechanism should be reserved for another study.

In the following description, we enumerate the similarities/dissimilarities between the Tanis Event-Deposit and other potentially applicable phenomena, including tidal influence, storm events, river flooding, and tsunami/seiche. Because of the close similarities in mode and tempo shared by onshore seiche and tsunami inundations, the sedimentological descriptions for tsunamite are equally diagnostic for a seiche deposit.

Sedimentological comparison between Tanis and tsunami/seiche. Sedimentologically, the deposit at Tanis closely resembles tsunamite (**Fig. S9**), which is typically distinguished by exhibiting: a) rapid sedimentation by a short-lived event involving one or several surge pulses of short duration, b) evidence of 180° change in flow direction, c) inclusion of highly atypical allochthonous bioclasts, and d) a contextually anomalous, turbulently deposited, and normally graded sediment package (***11, 13***). Multiple subsidiary criteria (**Table S9**), described below, are useful in diagnosing tsunamite, and reveal a close sedimentological resemblance to the Tanis Event-Deposit.

Sedimentary structures at Tanis (including bedforms, truncated flame structures, cross-bedding), and the orientation of biological debris (such as branches, fronds, and animal carcasses), record at least two 180° changes in flow direction during deposition (**Fig. S7**). Bidirectional flow, which represents inundation and backflow phases, is considered an indicator of tsunami-like inundation and contrasts with river flooding or typical wave-action during storms (***12, 13***). Bidirectional flow is also documented in tidalite deposits during incoming and outgoing tides (***68***), however these deposits differ greatly from tsunamites (and Tanis) in both structure and origin, in part because tidalites record many repetitive flow reversals via multiple stacked reactivation surfaces and graded lamina bundles, laid down repetitively over long periods of time, and are typically heavily bioturbated between each daily cycle. Flow reversal can also be associated with storm events, however it does not commonly occur multiple times throughout the sediment column as at Tanis, but rather occurs only at the end of the storm surge (***13***).

The turbulently deposited sediment package at Tanis dropped rapidly out of suspension, and is markedly distinct from the contextual bounding strata. The event rapidly covered the subaerially exposed, bioturbated, well-drained ground

surface (**Fig. S1**), and the rapidity of the event is further supported by the carcasses and debris that cross-cut the entire deposit (**Fig. S27**), truncated flame structures, climbing ripples, and absence of bioturbation. The brevity of the event is compatible with tsunami, which are short-lived events, with each surge pulse typically lasting only tens of minutes (***13***). By contrast, storm deposits typically form more gradually, over a more prolonged period of time than tsunami (***13***). In addition, the depositional mode at Tanis (deposited rapidly out of suspension), and sediment package that is strongly distinct from the contextual bounding strata, both ally closely with tsunamites, which are recorded as lithology distinct from the contextual bounding strata (***13***), and are predominantly laid down out of suspension. This depositional mode contrasts with typical storm sediments, which are most commonly deposited as bedload (***13***). The extensive rafts of organic debris within the Event-Deposit, and capping it, are also compatible with a tsunami-like inundation surge, and are extremely rare in flood deposits (***13, 69***). The abundant, angular, pebble- to small boulder-sized rip-up clasts at Tanis, and sharp basal contact (**Fig. S5**), are consistent with tsunami deposits and distinguish the deposit from a storm event. Angular rip-up clasts and sometimes mud laminae are frequently seen in the basal portions of tsunami deposits (***11, 13***), but are highly uncommon for storm deposits, where they are largely absent even when abundant source material is available, due to breakdown and homogenization during constant repetitive wave action (***13***). The rip-up clasts at Tanis also contrast with normal tidalites.

At Tanis, the ~1.3 meter-thick sheet of the Event-Deposit forms a drape of consistent thickness over the angled paleotopography (**Fig. S1**), a diagnostic feature of tsunamite that is produced as the sediment-laden water column quickly releases entrained particles and debris out of suspension (***11, 13***). This broad drape contrasts with storm deposits, which are discontinuous, and settle out as scattered localized patches in macrotopographical low points (***13, 70***), and is uncharacteristic for river flood deposits (***13***). The deposit thickness is also inconsistent with both storm and river flood deposits, which are typically much thinner (***13, 70***). The run-up height at Tanis, at least ~10 meters, also compares favorably with tsunami, which frequently exhibit an inundation run-up of tens of meters (***13***). Run-up height greater than several meters is also extremely uncommon and uncharacteristic for storm events, which commonly display run-up height of one meter or less (***13***). This is supported by 130 years of historical records around the Gulf Coastal Plains where the majority of storm surge run-up heights were between ~1.3 and 2.7 meters (**71**). Even the largest on record, from an uncommonly forceful hurricane (Katrina), was ~8.47 to 10 meters high, slightly less than or marginally equivalent to Tanis (**71**), and did not result in sedimentation that resembled that at Tanis.

The Tanis Event-Deposit exhibits normal-grading, with a massive sand base, internal mud laminae, and few lithological subdivisions, all of which are consistent with tsunamite (***11, 13***). This sedimentology directly contrasts with storm deposits, which are characterized by scores of subhorizontal planar laminae that are packaged into many repeatedly graded lamina sets from persistent repeated short-period wave-action even during storm surges (***13***). Additionally, the broad, two-surge pulse at Tanis is compatible with the small number of massive waves that characterize tsunami (***11, 13***), and contrasts with the numerous, smaller waves that characterize storm events.

Several horizons of well-formed truncated flame structures at Tanis (**Fig. S8**), some composed almost entirely of fragmented plant matter, are nearly identical to examples from modern tsunami (***9***). The truncation occurred syn-depositionally in response to flow-deformation during landward inundation, indicated by the truncated tips pointing in an uphill gradient and directed inland (westward)— opposite the southeast flow direction of contemporaneous rivers or the downwslope direction of the point-bar. Truncated flame structures in a continental depositional setting, such as the ones at Tanis, are commonly cited as among the most diagnostic features of tsunami, and are routinely used to distinguish tsunamite from storm/storm surge deposits or river-flooding events, which do not characteristically produce such flame structures (e.g. ***9, 11***).

Anomalous marine taxa in a terrestrial sedimentary environment are considered an additional robust indicator of inundation from marine facies (***11, 34, 72***). The mixed palynological assemblage at Tanis (**Fig. S25**) reflects an amalgamation of contemporaneous taxa with slightly older, reworked taxa, a common observation in tsunamites, which nearly always introduce a mixture of coeval and slightly older/reworked marine sediments or organisms (***13***). Comparable to observations of modern tsunamites, abundance of marine palynomorphs at Tanis showed moderate variation (***12, 34-37***), with much poorer recovery and greater damage in the lower, more-energetic units. Peters & Jaffe (***11***) interpreted this as an effect of variations within the mixed source-water, scoured bottom-sediments, and hydrological dynamics during sedimentation for similar variations seen in modern tsunami deposits (***34, 36***). Mollusk fragments, the most common marine bioclasts reported from both historic and paleotsunami tsunami deposits (***34***), are widely dispersed at Tanis, a distribution allied with onshore inundation (***13***), and in stark contrast with discrete laminae of shells that are characteristic of storm deposits (***13***).

Calculation of tsunami travel time from Chicxulub to Tanis. Matsui et al. (*73*) previously simulated the Chicxulub tsunami within the proto-Gulf of Mexico region, calculating a ~10 hour travel time from Chicxulub to the Mississippi Embayment. To calculate the remaining travel time to Tanis, we used the following equation of Halif & Sabki (*74*), which they used to calculate the speed of a shallow water tsunami wave, to approximate the tsunami speed within the WIS:

$$V= \sqrt{g h_w}$$

where, V is the tsunami velocity, h_w is the bathymetric depth, and g is gravity, outputting velocity under frictionless conditions and not considering drag or progressive attenuation as variables. While the exact paleobathymetric depth of the terminal-Cretaceous WIS is not known, reasonable estimates range from 100 to 400 m, which we considered as high and low thresholds for our calculations. This would result in tsunami speeds of ~111 to 223 km/h, taking ~8 to 16.4 hours to span from the proto Gulf of Mexico to Tanis. Added to the travel time already calculated by Matsui et al. (*73*) between the Chicxulub crater and the proto Gulf of Mexico, a tsunami would have taken a minimum of ~18 to 26.4 hours to make landfall in North Dakota. We point out that even if the tsunami traveled twice as fast, reaching Tanis within the same amount of time Matsui et al. (*73*) calculated for it to reach the Mississippi Embayment (about half the distance from Chicxulub to Tanis), the arrival would still have occurred far later than the time frame of the depositional event at Tanis indicated by incoming ejecta.

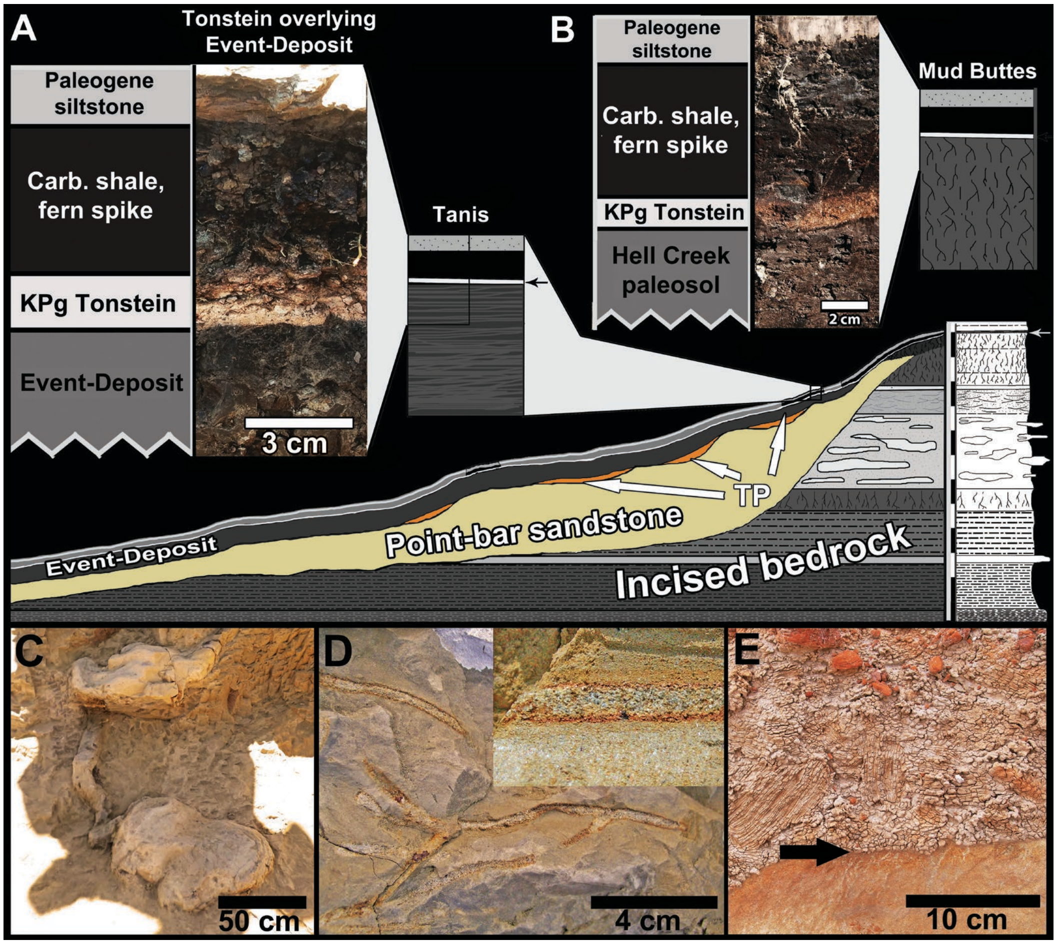

Fig. S1. Stratigraphic cross-section of Tanis, with select key features.

The Event-Deposit forms a drape of relatively even thickness, sharply overlying the angled point-bar slope, and is capped by a thin layer of tonstein overlain by a fern spore-dominated horizon (A), indistinguishable from Mud Buttes (B), and common for the Western Interior. Terrace paleosols (TP) underlying the Event-Deposit bear abundant pedogenic structures such as (C) vertebrate burrows, (D) invertebrate ichnofossils, and (E) oxidation halos, indicating prolonged subaerial exposure and occupation prior to inundation. Tunnels of insect burrows (D) are filled with sediment contiguous with the basal Event-Deposit and bear reddened oxidized walls, indicating subaerial exposure and open chambers up until the time of inundation. Stratigraphic scale in meters; Mud Buttes image courtesy Jeff Person, NDGS.

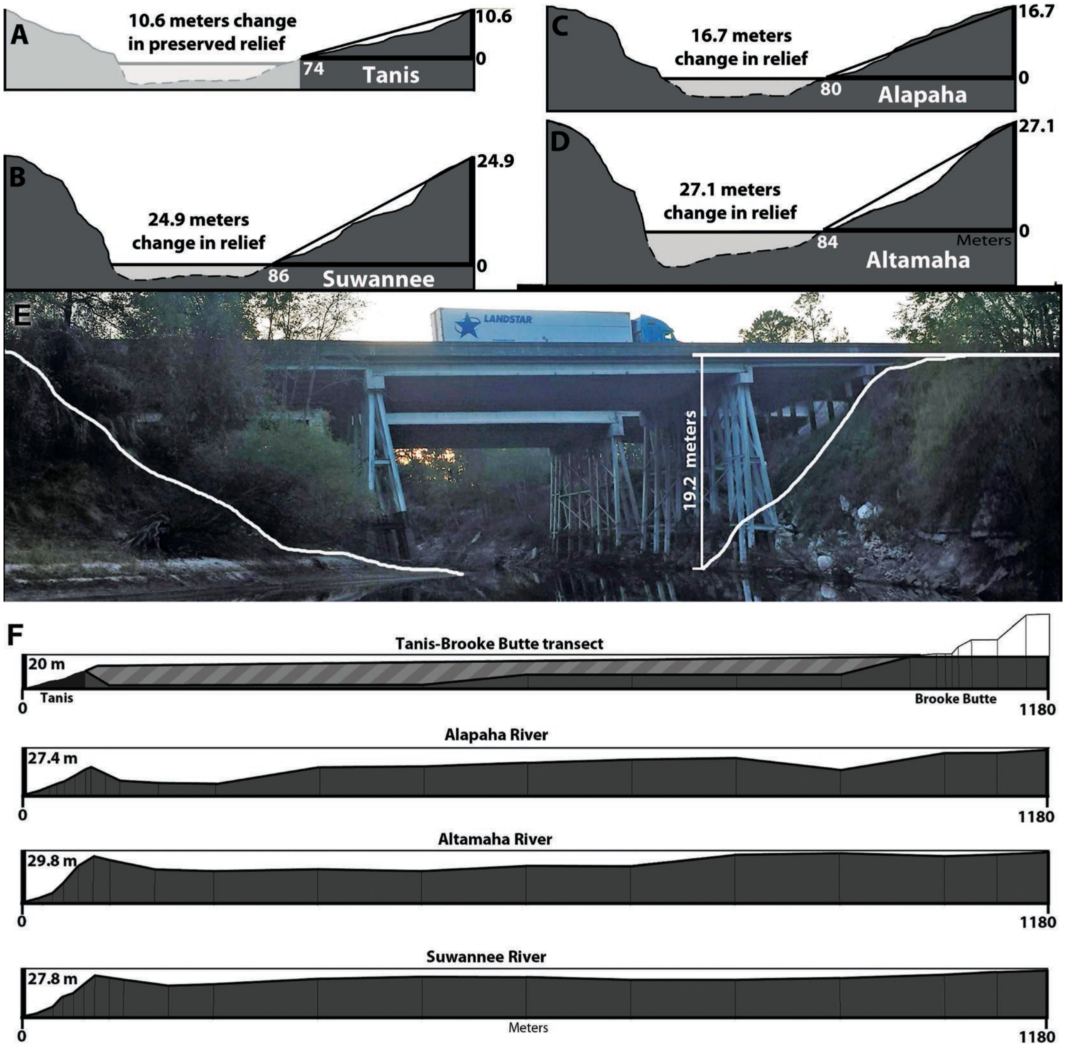

Fig. S2. Tanis gradient compared to modern river analogs.

The observed portions of the point-bar at Tanis (**A**, shaded) indicate at least ~10.6 meters of paleorelief. This compares favorably with proximal elevation change in modern sub-tropical rivers incised into soft substrate, such as the Gulf coastal plains. The steep point-bar embankments of modern analogs including (**B**) the Suwannee, (**C**) Alapaha, and (**D**) Altamaha rivers are nearly identical in scale to the preserved portions of Tanis, and they enable a conservative tentative reconstruction of the Tanis river valley (A). A modern Image of the Suwannee River (E), with a steep point-bar embankment, provides a very close analog to how Tanis would have appeared prior to inundation. An extended cross-section spanning from Tanis to the nearest KPg boundary outcrop at Brooke Butte (F, top), compared with modern cross-sections measured an equivalent distance from the river's waterline (F, below), shows a remarkable similarity in scale and elevation change over distance. Total elevation change and degree of change over a 1180 meter transect are compatible with observed data from the Tanis region, even exceeding the Tanis elevation change. Most of the observable transect between Tanis and Brooke Butte has been eroded down into older strata; the missing portions were reconstructed (striped area) by drawing a straight line between Tanis and the Brooke Butte KPg boundary, the most conservative approach possible. Cross sections shown with 2X vertical exaggeration, measurement in meters.

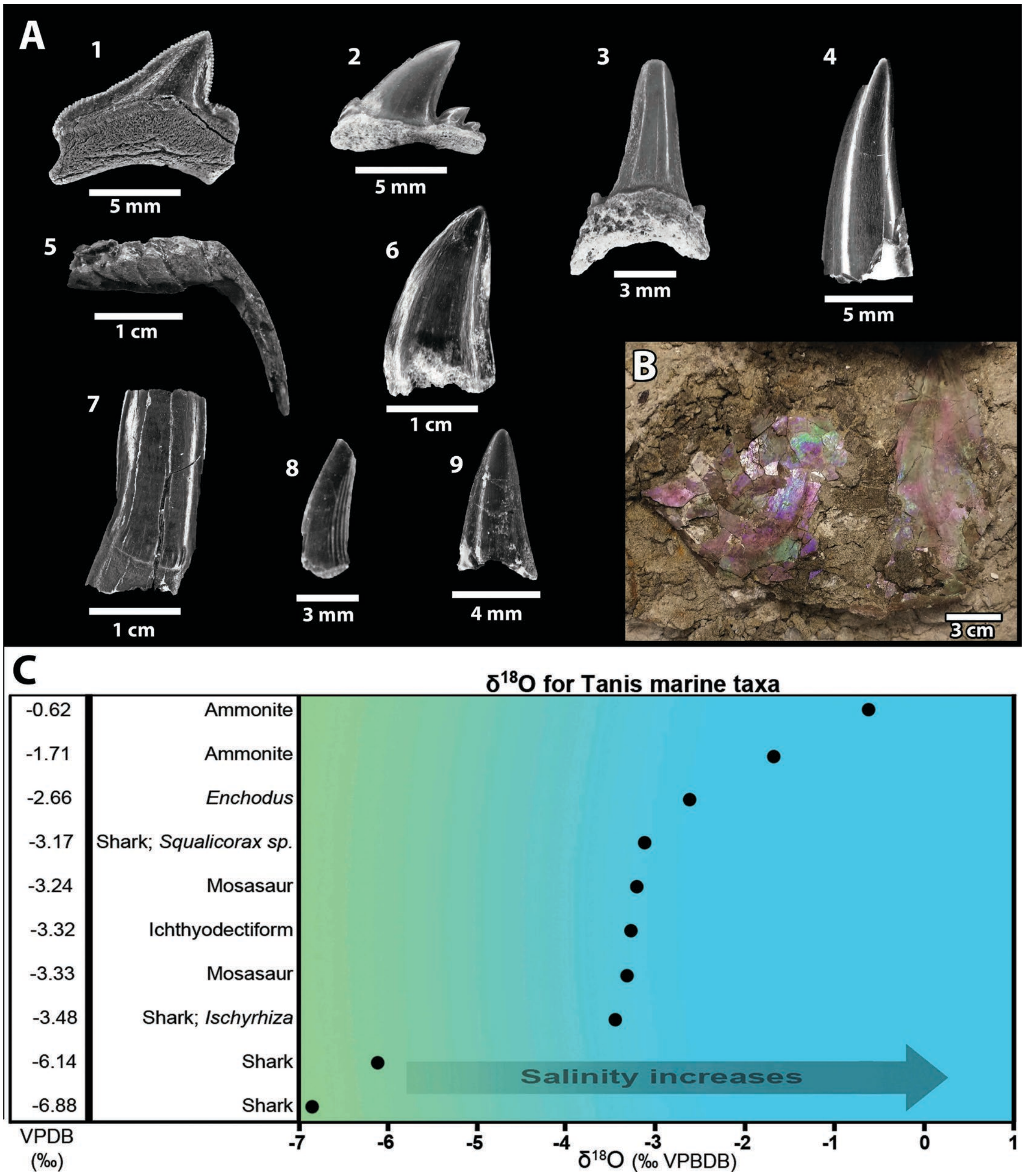

Fig. S3. Representative marine fossils from Tanis, uncharacteristic outside the marine tongues in the Hell Creek Formation.

(**A**), teeth of marine vertebrates, including (1-3, 9), sharks; (4), Ichthyodectiform fish; (5), *Enchodus*; (6), Mosasauridae indet.; (7), sawfish *Ischyrhiza*; and (8), *Enchodus*. Marine mollusks, primarily the ammonite *Sphenodiscus lobatus,* have been found as isolated fragments and large sections of partial shells (**B**). Note the nacreous preservation, which does not occur in mollusk shells in the Hell Creek marine tongues. (**C**), stable isotope data for suspected marine fossils, demonstrating their brackish to fully marine provenance. (A1-9): FAU.DGS.ND.161.433.T, FAU.DGS.ND.161.312.T, FAU.DGS.ND.161.719.T, FAU.DGS.ND.161.152.T, FAU.DGS.ND.161.133.T, FAU.DGS.ND.161.137.T, FAU.DGS.ND.161.139.T, FAU.DGS.ND.161.332.T.

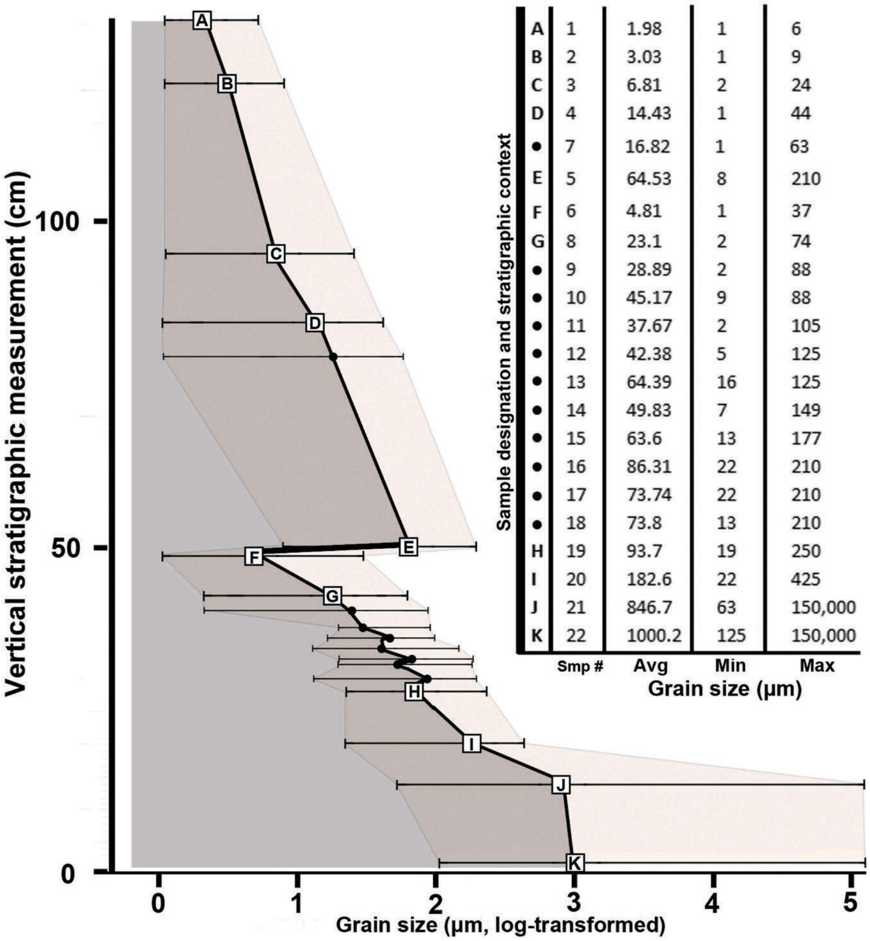

Sample designation and stratigraphic context	Smp #	Avg	Min	Max
A	1	1.98	1	6
B	2	3.03	1	9
C	3	6.81	2	24
D	4	14.43	1	44
●	7	16.82	1	63
E	5	64.53	8	210
F	6	4.81	1	37
G	8	23.1	2	74
●	9	28.89	2	88
●	10	45.17	9	88
●	11	37.67	2	105
●	12	42.38	5	125
●	13	64.39	16	125
●	14	49.83	7	149
●	15	63.6	13	177
●	16	86.31	22	210
●	17	73.74	22	210
●	18	73.8	13	210
H	19	93.7	19	250
I	20	182.6	22	425
J	21	846.7	63	150,000
K	22	1000.2	125	150,000

Fig. S4. Grain-size profile for the Event-Deposit.

The grain size distribution shows an overall and repeated fining-upward trend. Data points are grain-size averages, bars outline minimum and maximum values. Table shows raw data from the designated stratigraphic intervals.

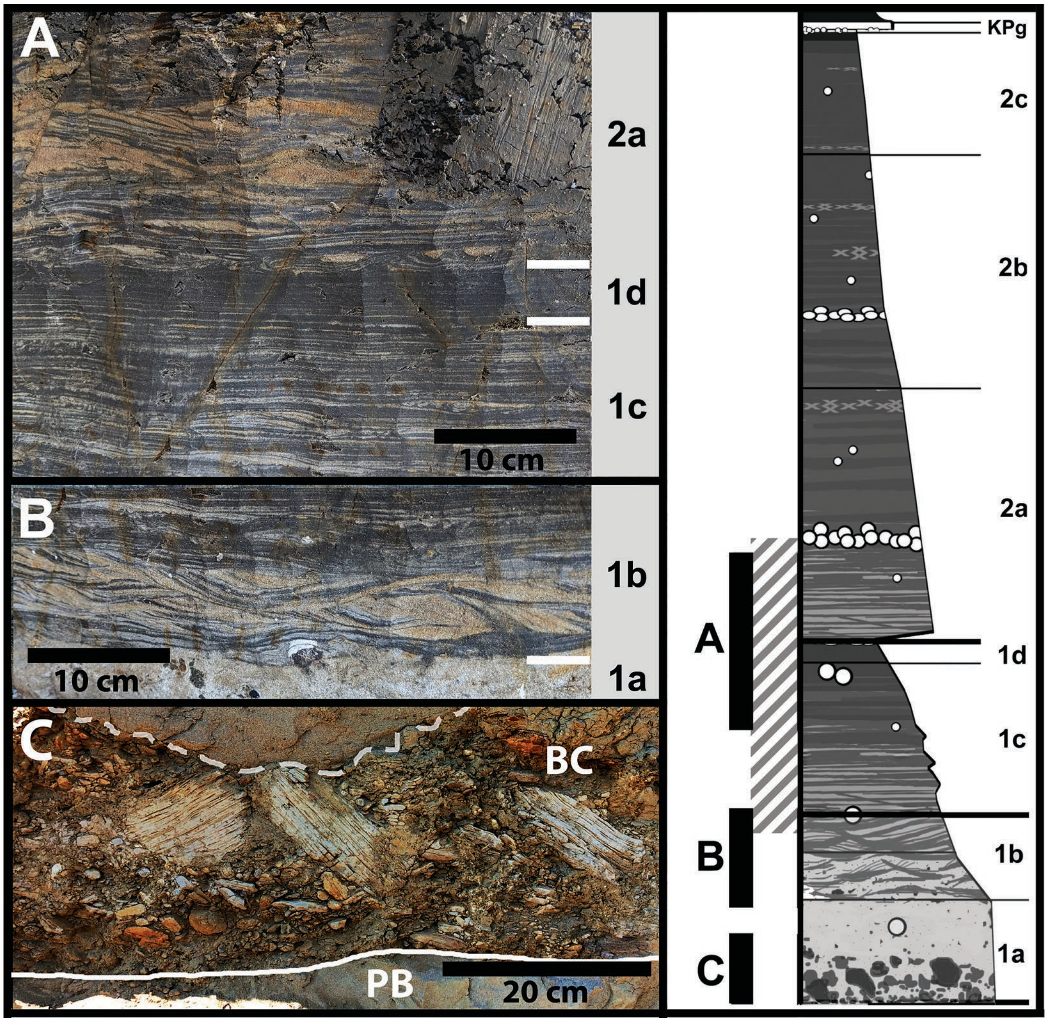

Fig. S5. Basal Unit 1 of the Event-Deposit.

(**A**), Pronounced grading from interlaminated medium- to fine-grained sediments (1c) to a dark band of fine-grained silt/mud (1d), followed by an additional pulse of laminated medium- to coarse-grained sediments (2a), illustrating a waning and then resurgence of system energy, which characterizes the Unit 1-2 transition. (**B**), Detail of Unit 1b, showing steady decrease in grain size and turbulence from base to top. (**C**), Basal conglomerate (BC) with turbulently deposited large rip-up clasts in lower Unit 1a, sharply contacting the point-bar surface (PB). Cartoon at right shows regions of focus in the images, striped regions indicate stratigraphic level of greatest carcass abundance.

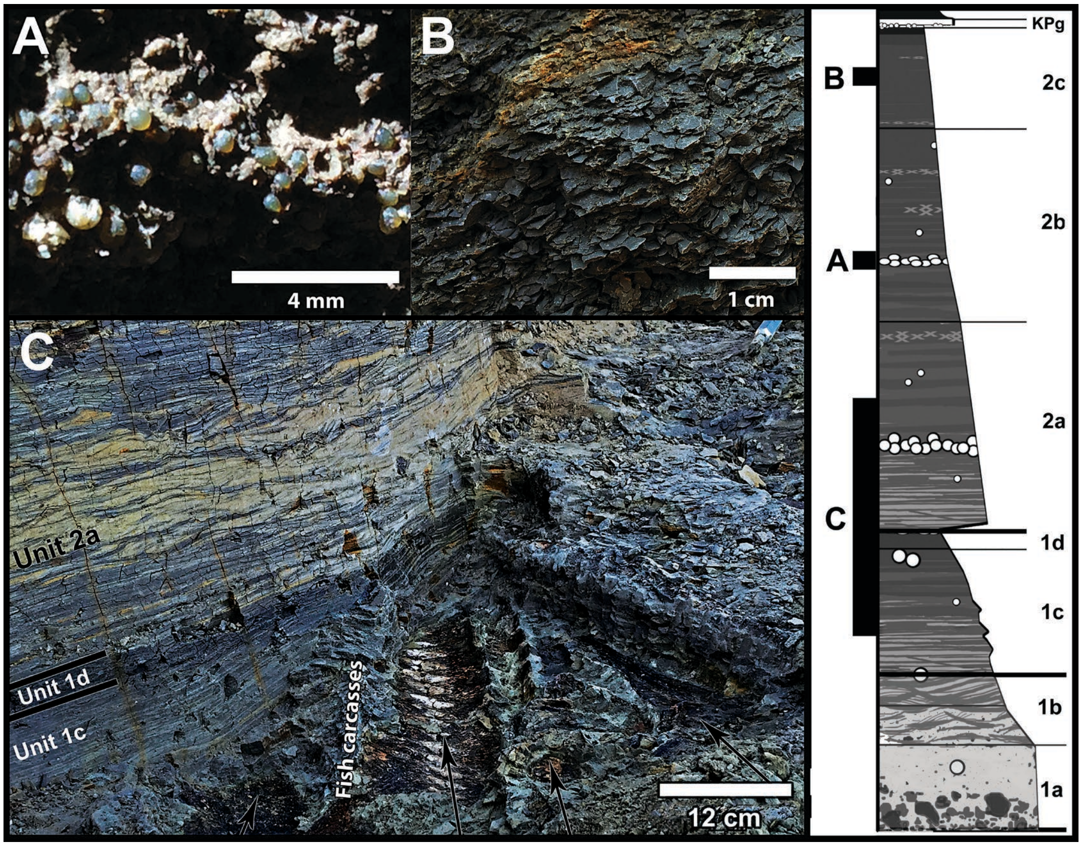

Fig. S6. Unit 2 detail and cross-section.

(**A**), Field photo of a spherule lens in situ (FAU.DGS.ND.161.88.T), from upper-middle Unit 2. (**B**), Detail of upper Unit 2c, showing the highly fissile, flaky texture and very fine grain size. (**C**), Field photo of cross-section showing uppermost Unit 1 transitioning to Unit 2. Arrows point to fish carcasses. Cartoon at right shows regions of focus in the images.

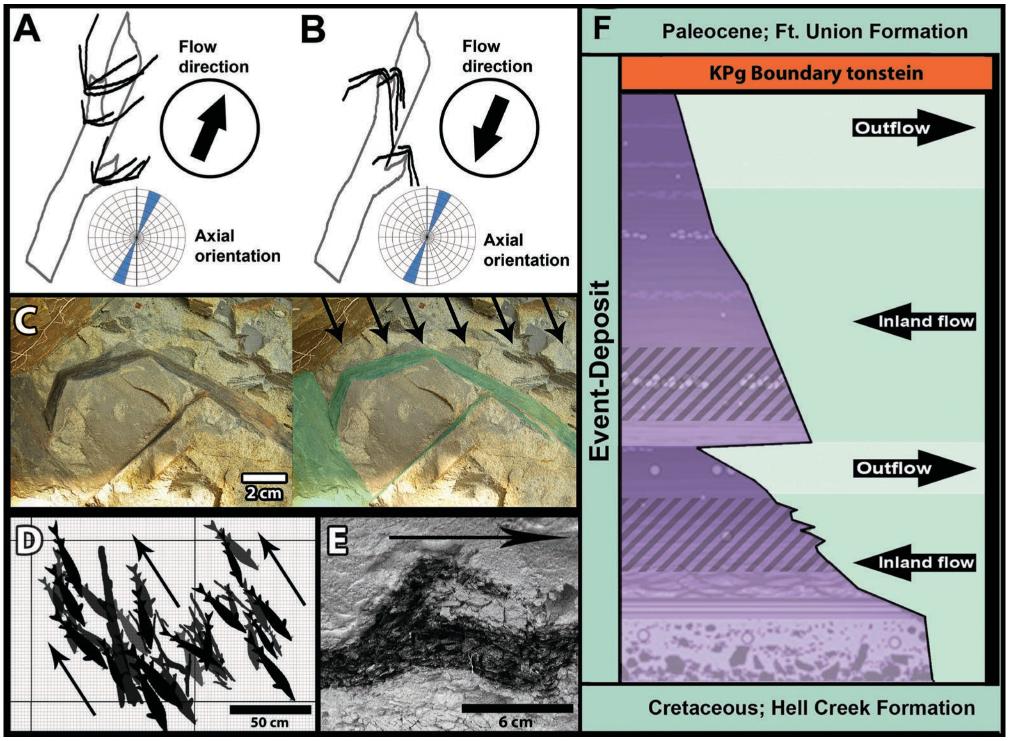

Fig. S7. Select flow-indicators and a simplified cross-section of the Tanis site profile.

(**A, B**), The axial direction of elongated, robust plant debris (i.e. logs, branches, twigs) can indicate that they were oriented by flow currents, however does not necessarily clarify which direction along the 180° axis that the flow came from, unless combined with data from other bio-debris that was bent by the same current. For example, axial orientation of the log in (A) and (B) is identical, however pliable floral debris bent by the flow current indicates a northeastern flow direction for (A), and a southwestern (opposite) flow direction for (B). (**C**), Field photo and interpretive overlay of fronds bent by water current. Orientation of carcasses (**D**), and truncated flame structures (**E**), offer additional indication of flow direction. Arrows = direction of flow. (**F**), Simplified cross-section of the Event-Deposit, showing bi-directional flow, regions of greatest carcass abundance (striped), and stratigraphic placement between the Hell Creek and Ft. Union Formations.

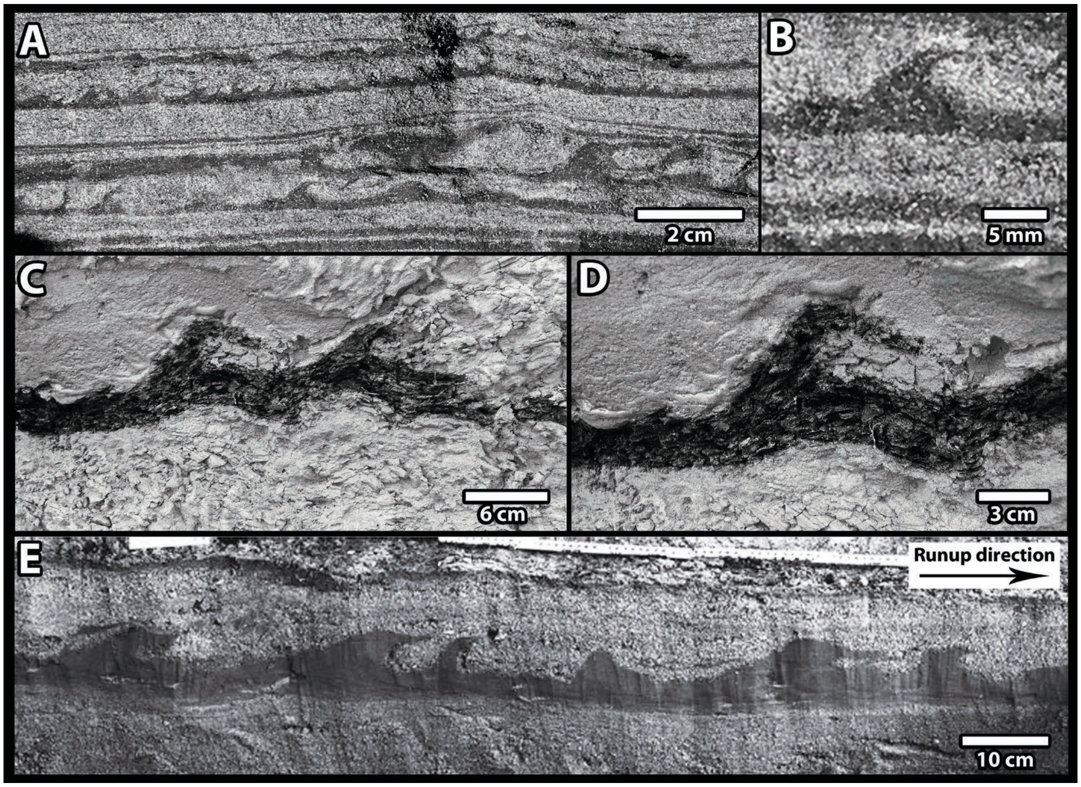

Fig. S8. Truncated flame structures at Tanis.

Truncated flame structures from Tanis (**A**-**D**) are syn-depositional indicators of flow direction, and are uncharacteristic for storm or river-flood deposits. (**C** and **D**), exceptionally dark examples due to high content of plant debris. (**E**), a near-identical example of onshore-deposited truncated flame structures from the 2004 Indian Ocean tsunami (from Matsumoto et al. ref. ***9***). These syn-sedimentary structures are considered by some studies (e.g. ***9, 11***) to be among the most-diagnostic features of onshore inundation. (See Matsumoto et al [***9***] for a more-comprehensive treatment of truncated flame propagation during tsunami events).

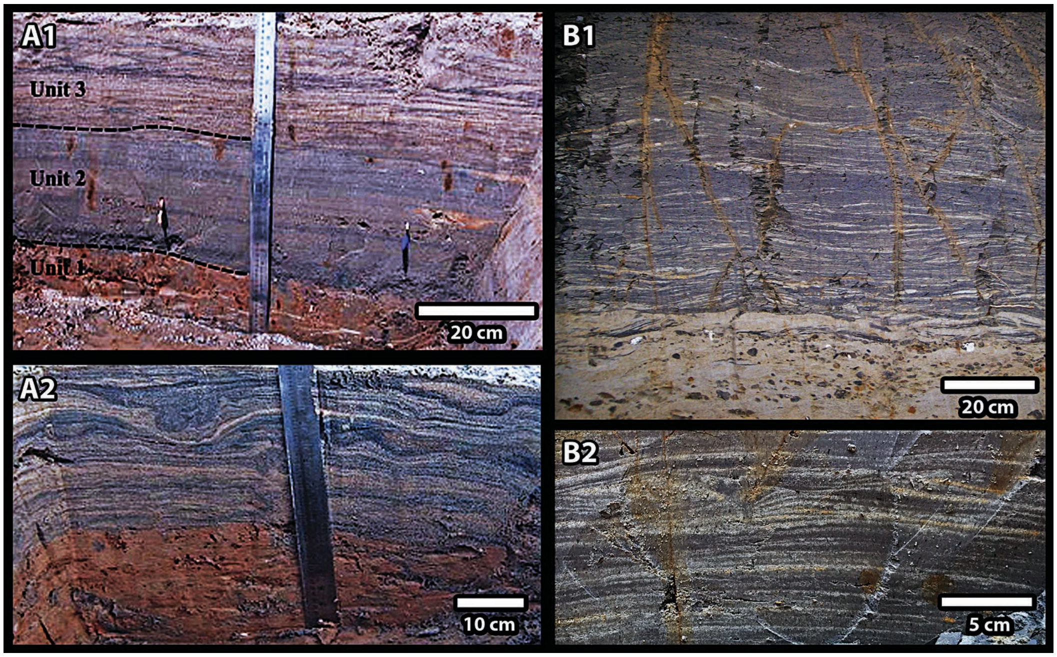

Fig. S9. Visual similarity between Tanis and modern tsunami deposits.

(**A**1, 2),Cross-sections from the southeastern India Tamil Nadu coast deposited by the 2004 Indian Ocean Tsunami show alterations of fine sand and silt laminations and overall structure similar to the deposit at Tanis (**B**1, 2). Images A1, 2 reprinted with permission from ref. ***10***.

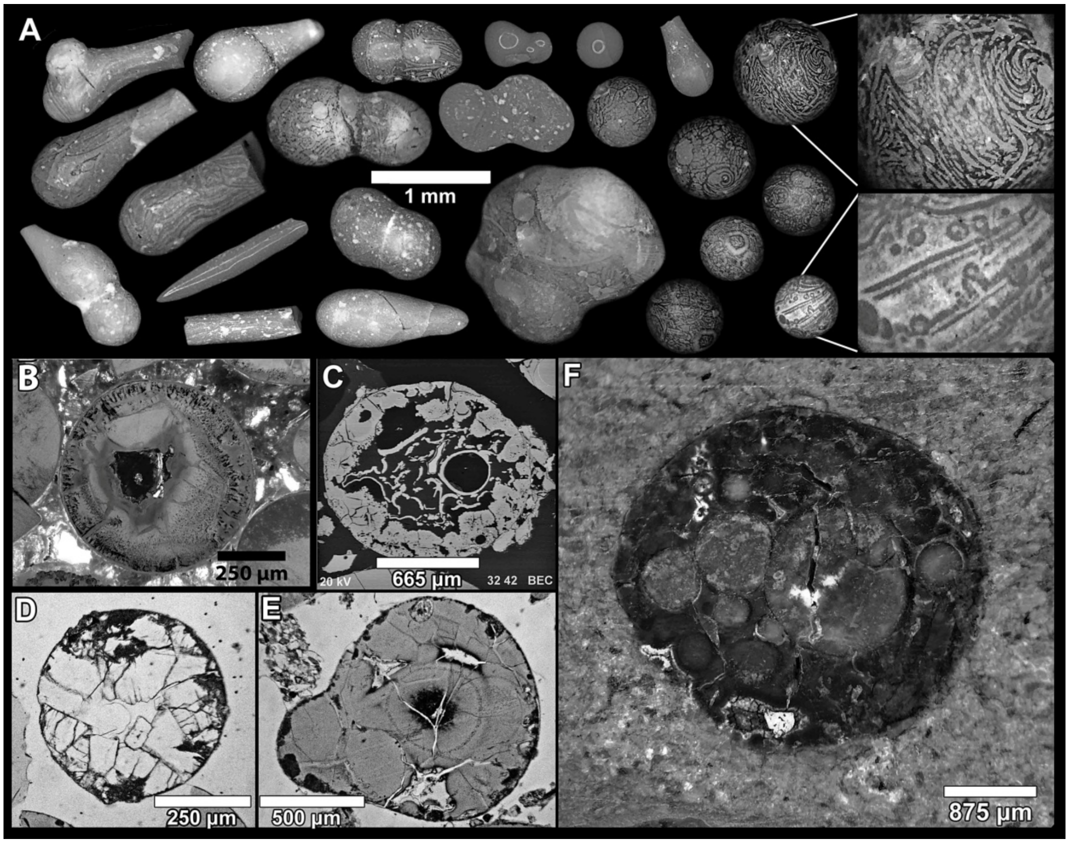

Fig. S10. Ejecta spherules from the Tanis Event-Deposit and tonstein.

(**A**), spherules represent a diversity of aerodynamic shapes, including teardrop, elongated rod, welded dumbbell, amorphous globule, and spherical impact-melt spherules, diagenetically altered from glass to smectitic clay (FAU.DGS.ND.161.33.T). (**B**), photomicrograph of an isolated spherule in thin section (FAU.DGS.ND.161.507.T). (**C**), SEM backscatter electron image of another isolated spherule in thin section (FAU.DGS.ND.161.339.T). (**D**), thin section of a calcite-replaced spherule, rare at the site FAU.DGS.ND.161.664.T). (**E**), thin section of two spherules that welded together while still molten (FAU.DGS.ND.161.619.T). (**F**), sectioned and polished spherule in situ from the outcrop (FAU.DGS.ND.161.619.T).

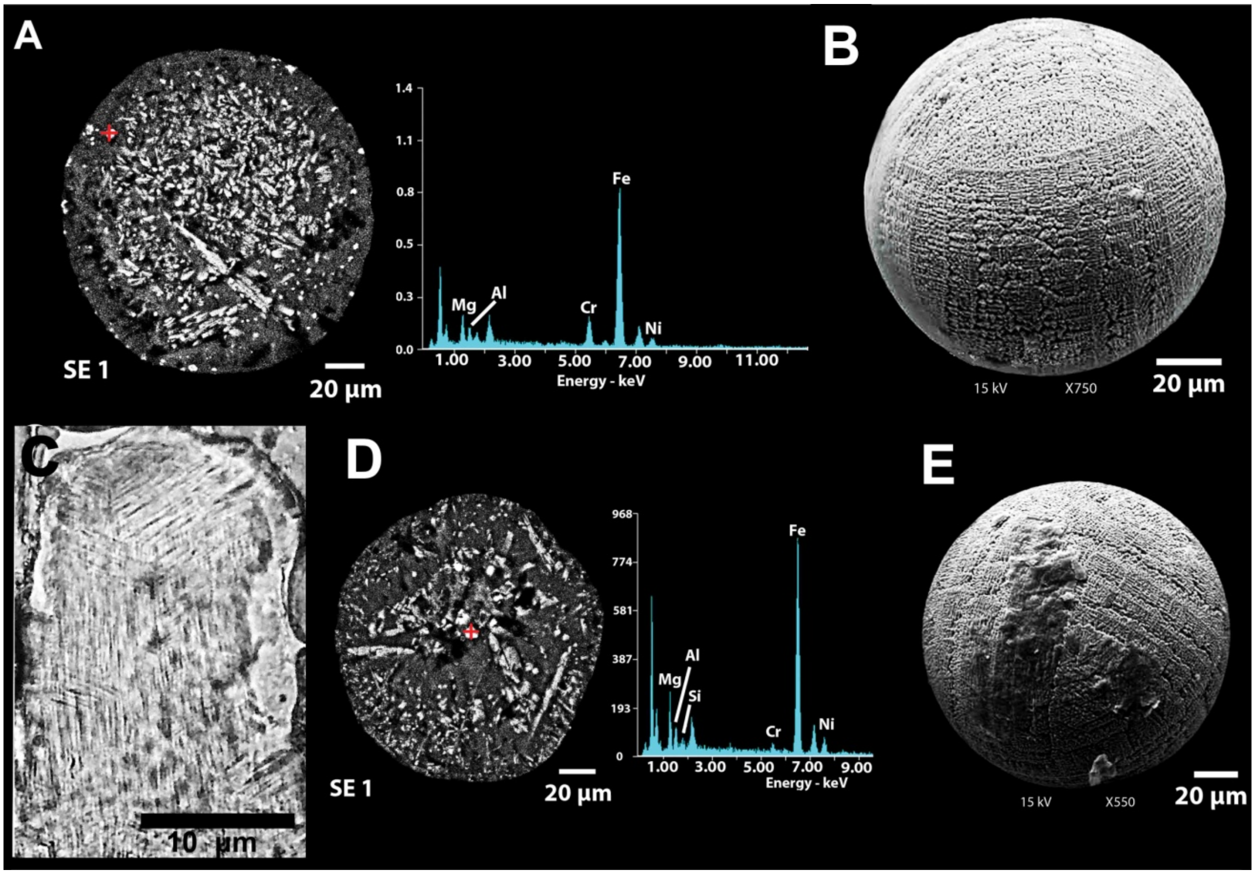

Fig. S11. Microkrystites and shocked minerals from Tanis.

(**A, D**), SEM secondary electron images of Tanis microkrystites in thin section (FAU.DGS.ND.161.550.T, FAU.DGS.ND.161.710.T), showing the complex internal microstructure and well-defined inclusions of iron enriched with chromium and nickel (cross-hairs). (**B, E**), external morphology of two different microkrystites (FAU.DGS.ND.161.687.T, FAU.DGS.ND.161.992.T), showing prominent quench textures. (**C**), Shocked quartz from the KPg tonstein overlying the Event-Deposit (FAU.DGS.ND.161.3.T).

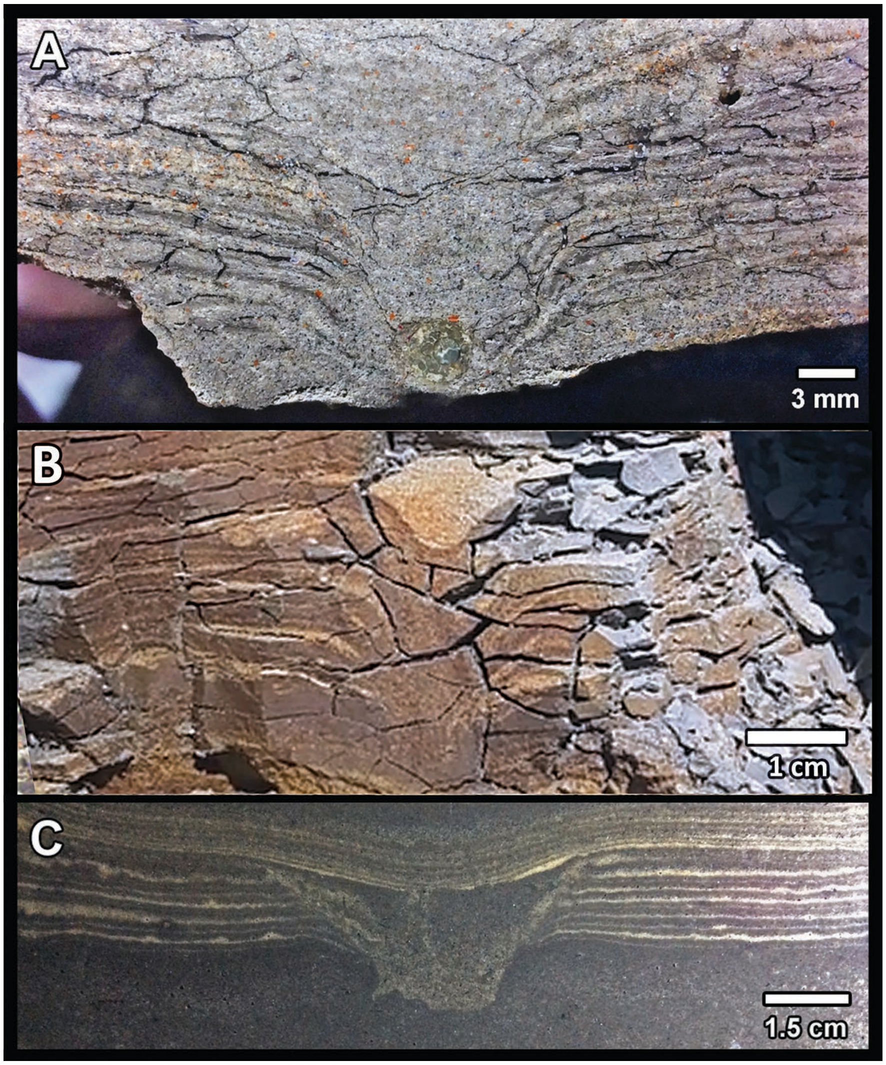

Fig. S12. Syn-depositionally down-warped sediment probably caused by falling impact-melt spherules.

(**A** and **B**), warped cones of focally disturbed laminae in Unit 1c; a single impact-melt spherule can be seen at the base of (A). (**C**), an experimental run in which a glass bead matching the spherule size was plunged into sieved Tanis sediment that was laid down to replicate the natural laminations, resulting in a similar warped structure. (A) photo courtesy Jan Smit. (A): FAU.DGS.ND.161.619.T.

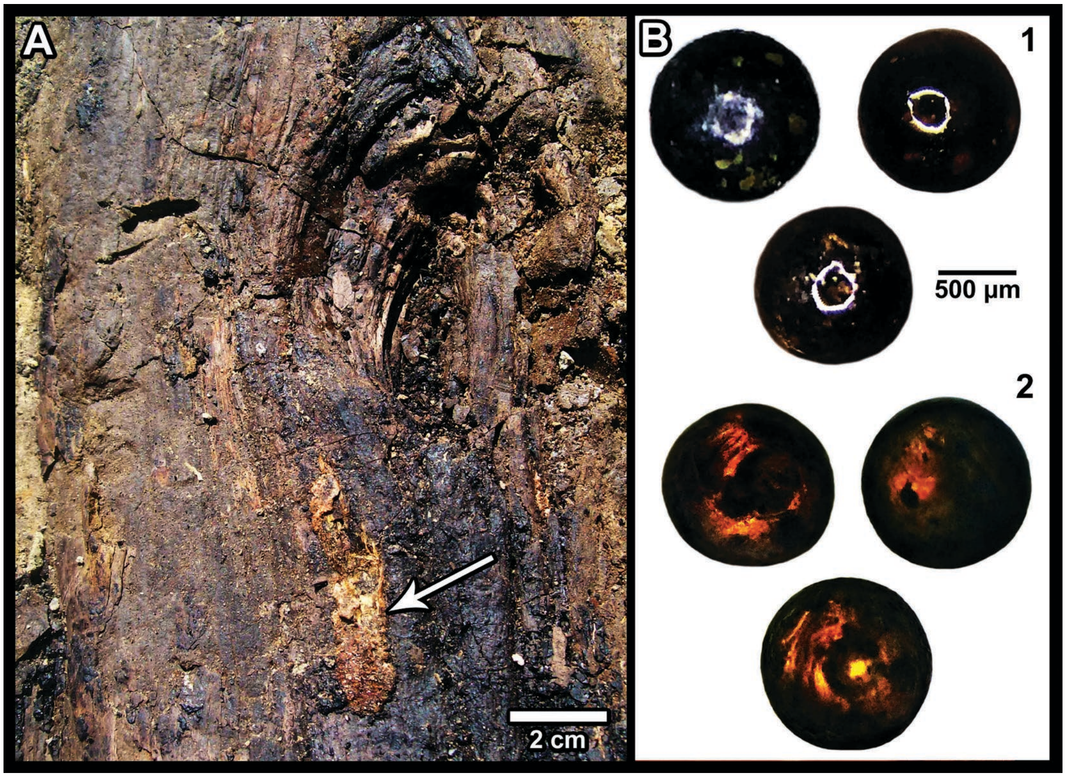

Fig. S13. Amber and unaltered ejecta spherules.

(**A**), an amber runnel on a large fossil tree trunk at Tanis (arrow points to runnel). (**B**), glassy spherules isolated from the amber are seen in overhead light (**B1**) and with light transmitted from beneath (**B2**), showing the translucency of the glass and internal vesicles. The spherules appear to be pristine and unaltered. (B): FAU.DGS.ND.161.109.T.

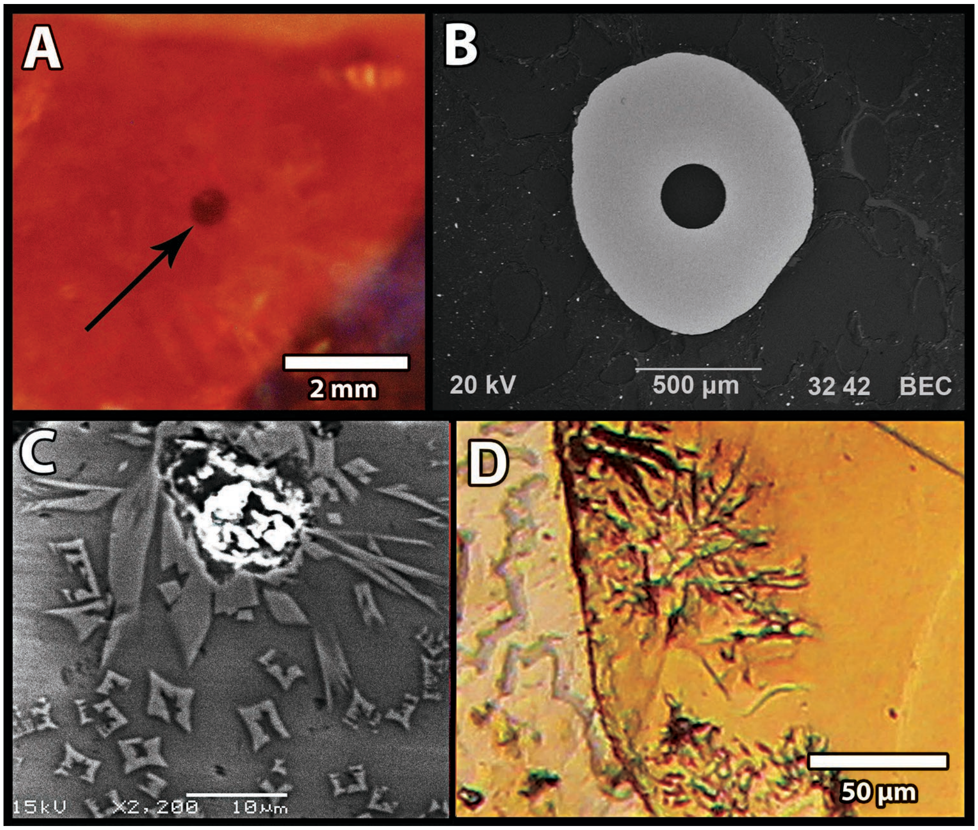

Fig. S14. Amber-spherule SEM, petrographic, and light microscopy.

(**A**), photomicrograph of a spherule still completely embedded in amber (FAU.DGS.ND.161.77.T); (**B**), SEM backscatter image, of a thin-sectioned spherule in amber (FAU.DGS.ND.161.269.T), revealing the smooth, largely homogenous internal structure identical to unaltered glass shards from the spherule cores. (**C**), magnified SEM backscatter image of a glassy spherule rim, showing a cluster of well-defined melilite crystals (FAU.DGS.ND.161.1073.T), which are similar to relict examples seen in diagenetically altered Chicxulub spherules (**D;** FAU.DGS.ND.161.1052.T).

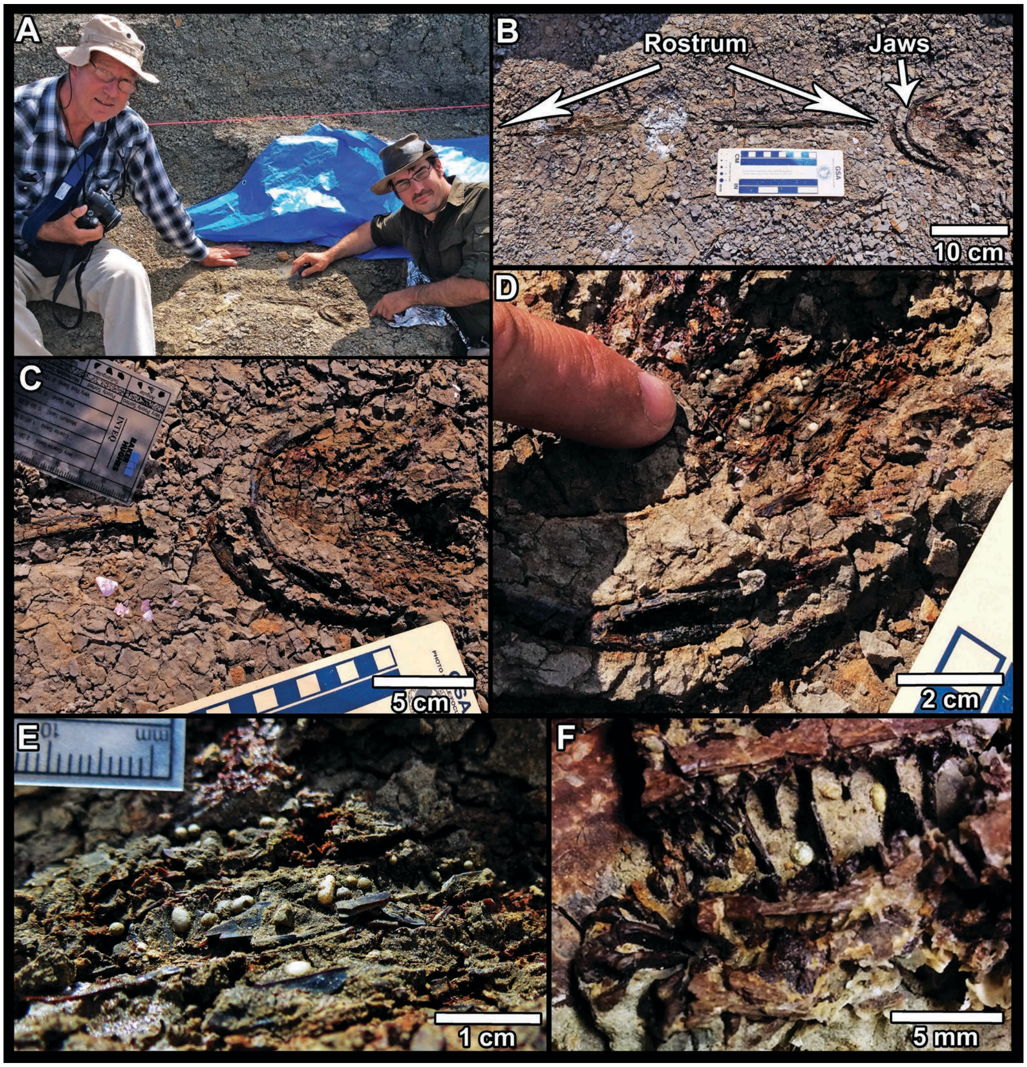

Fig. S15. Spherule-enrichment in the gills of a polyodontid fish (paddlefish).

(**A**), Initial excavation and examination of paddlefish in situ; (**B**-**E**), magnified views of the gill region showing impact-melt spherules clustered between the gill bars. Purple shards in (C) are fragments of ammonite shell; (**F**), magnified view of a sturgeon, partially prepared to reveal the round spherules between the gill rakers. Photo in (A), courtesy Kylie Ruble; B-E, courtesy Jan Smit.(A-E): FAU.DGS.ND.161.688.T, (F): FAU.DGS.ND.161.115.T
.

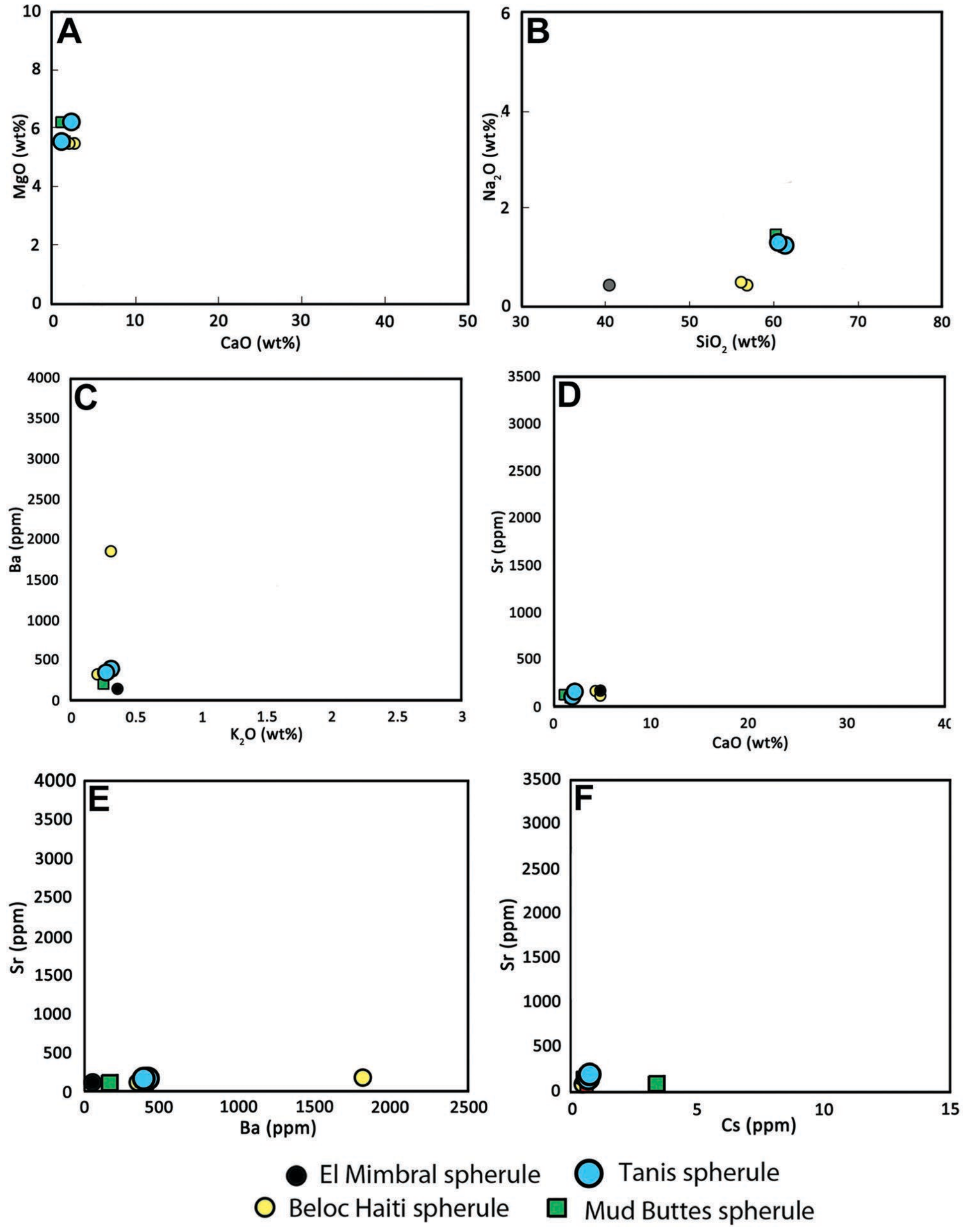

Fig. S16. Bivariate graphs of diagenetically altered spherules from Tanis, compared to KPg examples from elsewhere.

(**A**-**F**),Tanis data added to graphs reprinted with permission from Belza et al. (*22*), illustrating the similarity between Tanis alteration products and Chicxulub spherules from elsewhere.

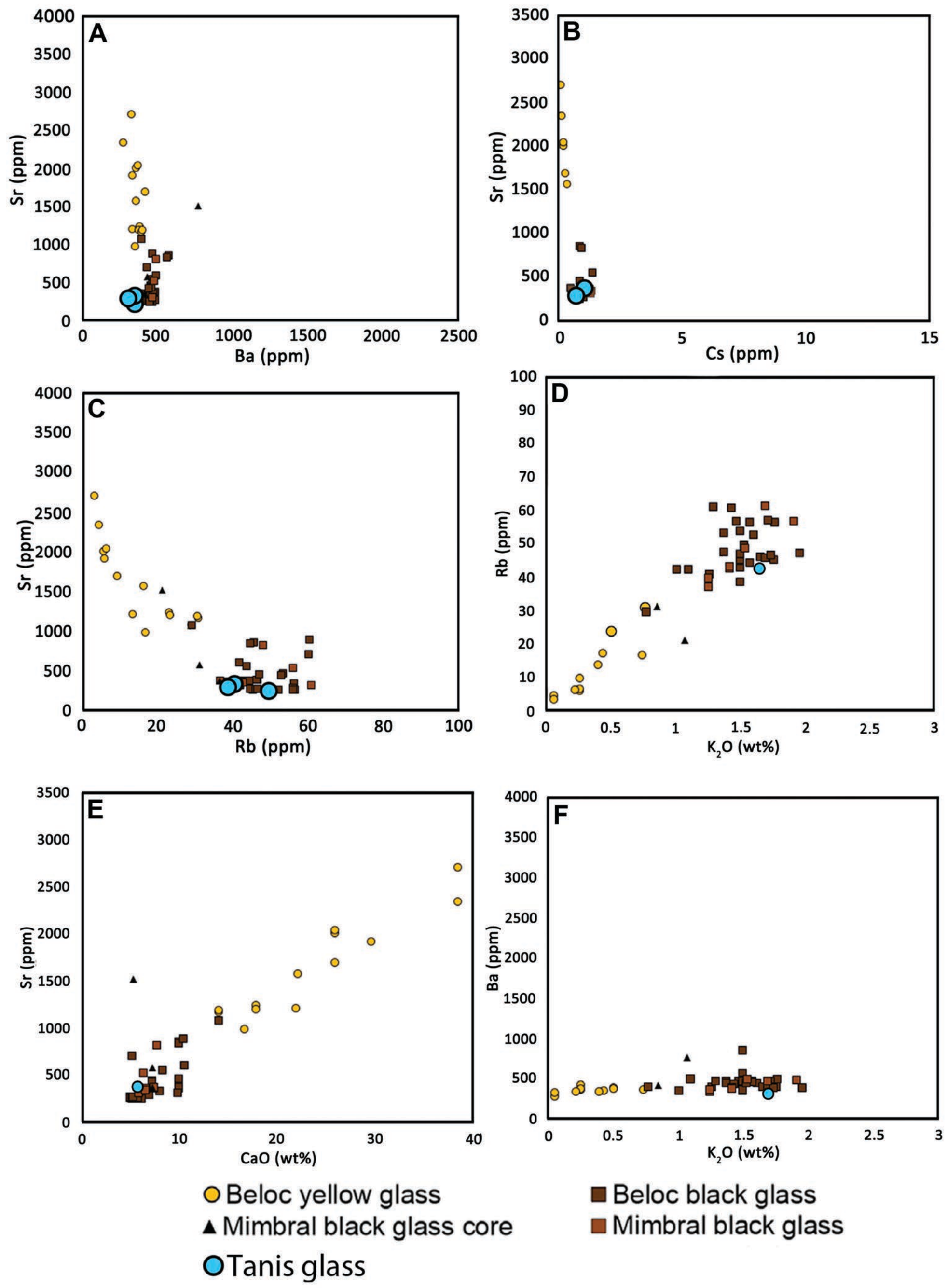

Fig. S17. Bivariate graphs for unaltered glass from Tanis.

(**A**-**F**),Tanis data added to graphs reprinted with permission from Belza et al. (*22*), illustrating the similarity between Tanis glass and ranges exhibited by Chicxulub black glass.

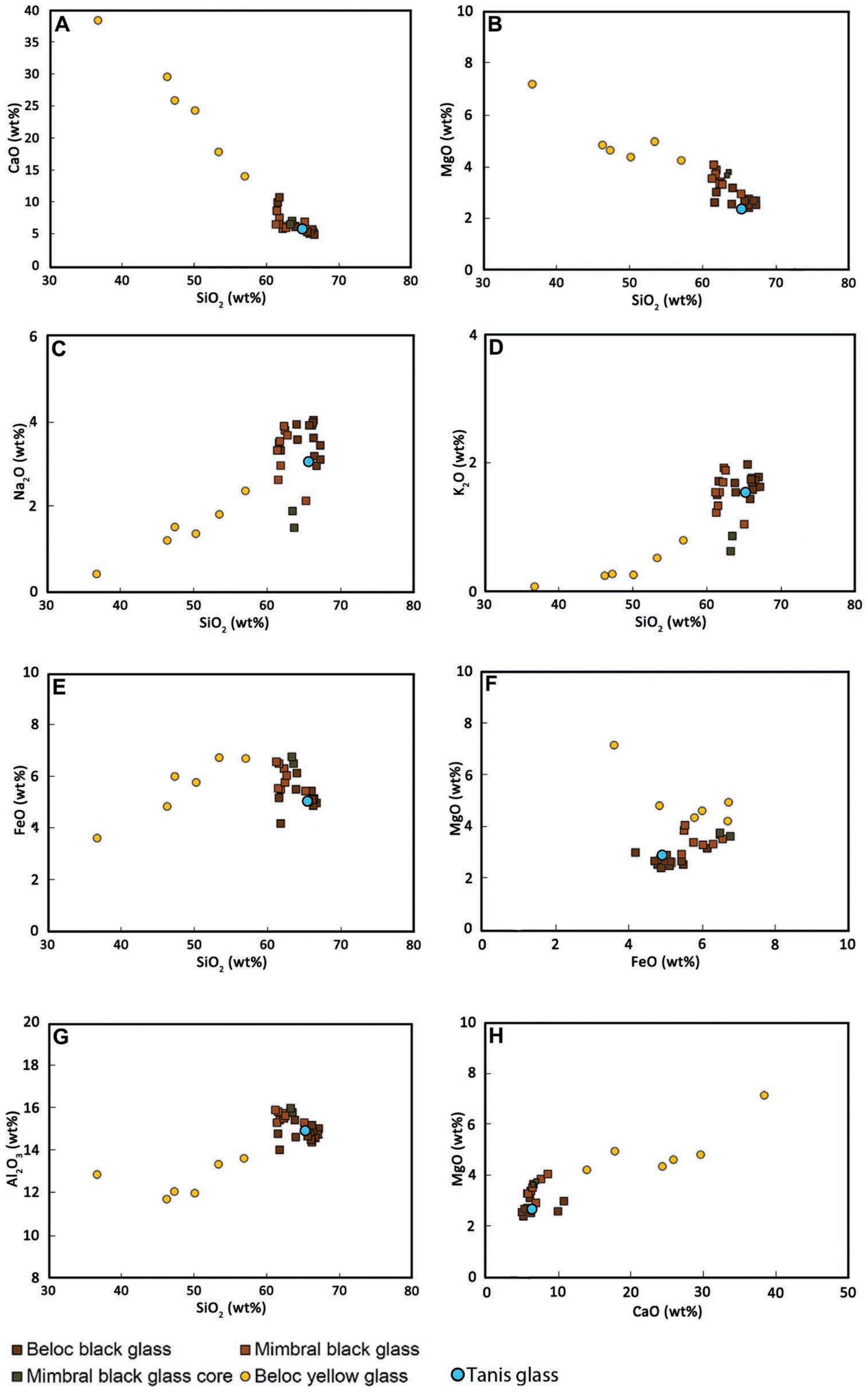

Fig. S18. Bivariate graphs for unaltered glass from Tanis.

(**A-H**), Tanis data added to graphs reprinted with permission from Belza et al. (*22*), illustrating the similarity between Tanis glass and ranges exhibited by Chicxulub black glass.

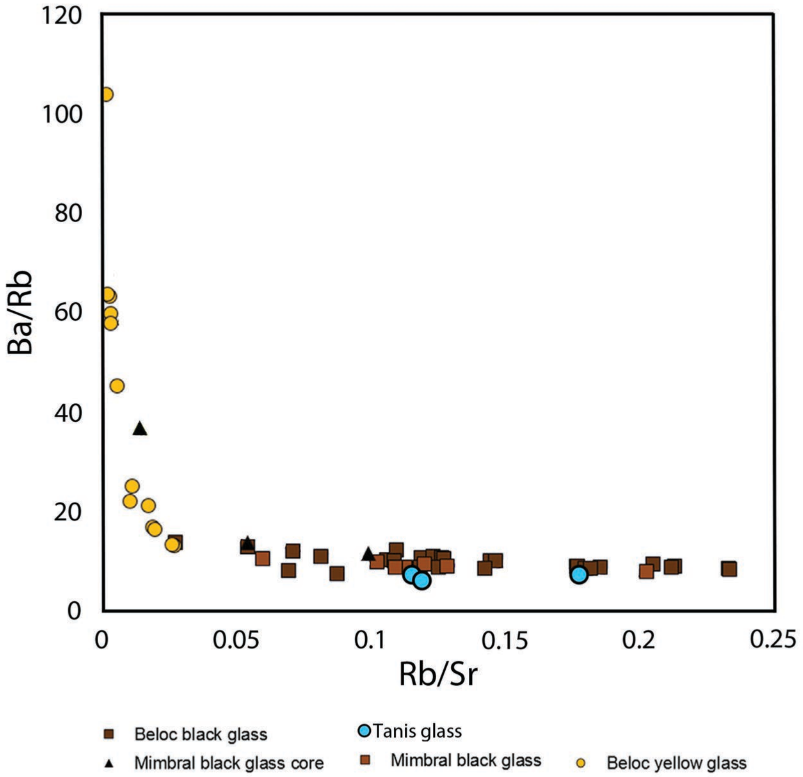

Fig. S19. Ba/Rb versus Rb/Sr plot for unaltered glass from Tanis compared to El Mimbral and Haiti.

The Tanis specimens ally with the ranges for Chicxulub black glass. Data added to graphs reprinted with permission from ref. Data added to graph reprinted with permission from ref. ***22***.

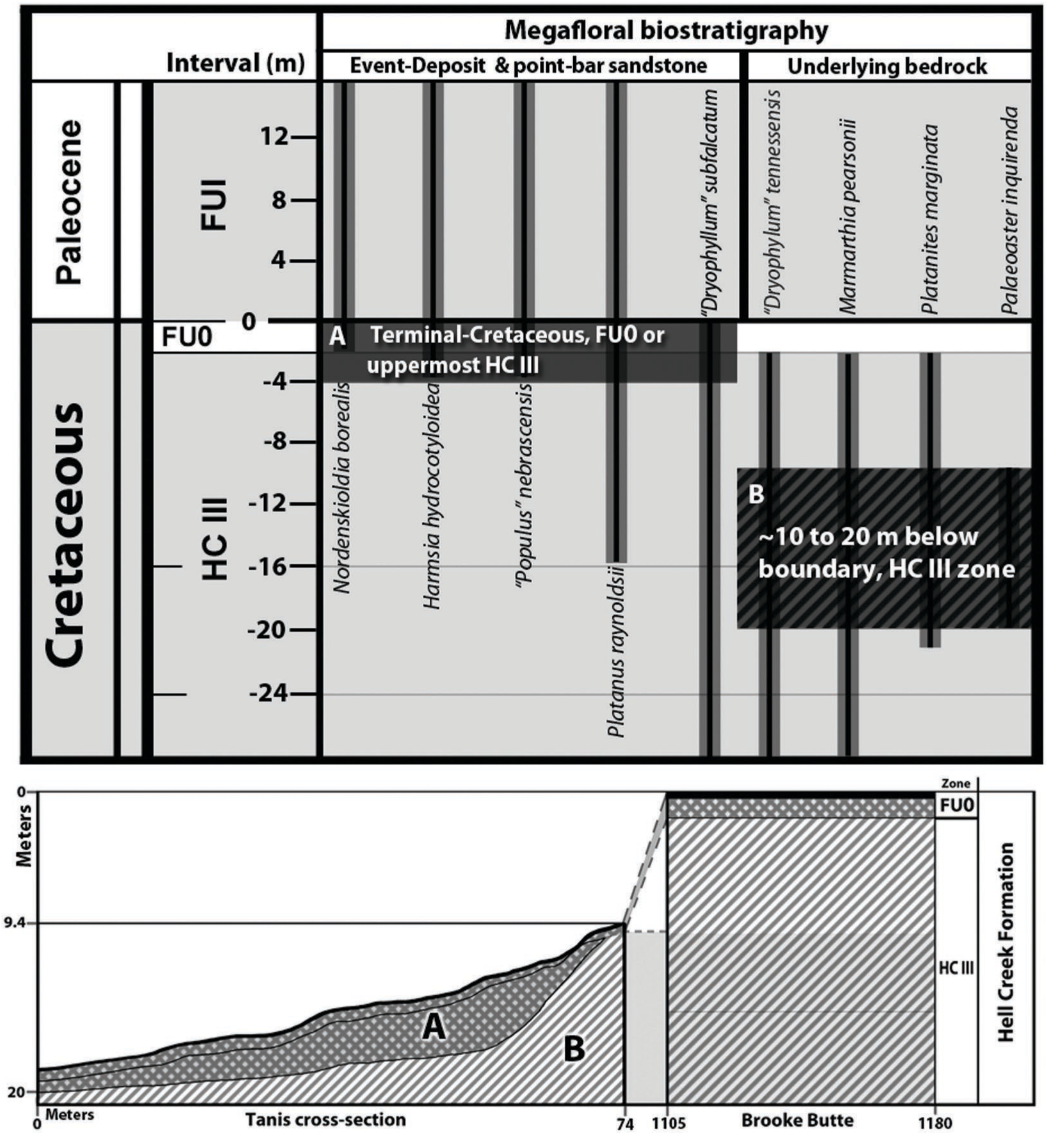

Fig. S20. Megafloral biostratigraphy of the Tanis Event-Deposit, point-bar sandstone, and incised bedrock, showing some representative key megafloral markers.

Megaflora of the incised bedrock (**B**) is consistent with the upper ~10 to 20 meters of the Hell Creek (HCIII floral zone). However, the combined Event-Deposit and point-bar sands (**A**), do not match their depositional elevation and are allied with the much-higher terminal-Cretaceous uppermost HC III or FU0 floral zones, both of which can extend up to and include the KPg boundary. Deposition of the KPg-aged Event-Deposit and point-bar sands at a lower elevation than the surrounding local strata was a result of the incised valley depositional setting. Data from Johnson (***31***).

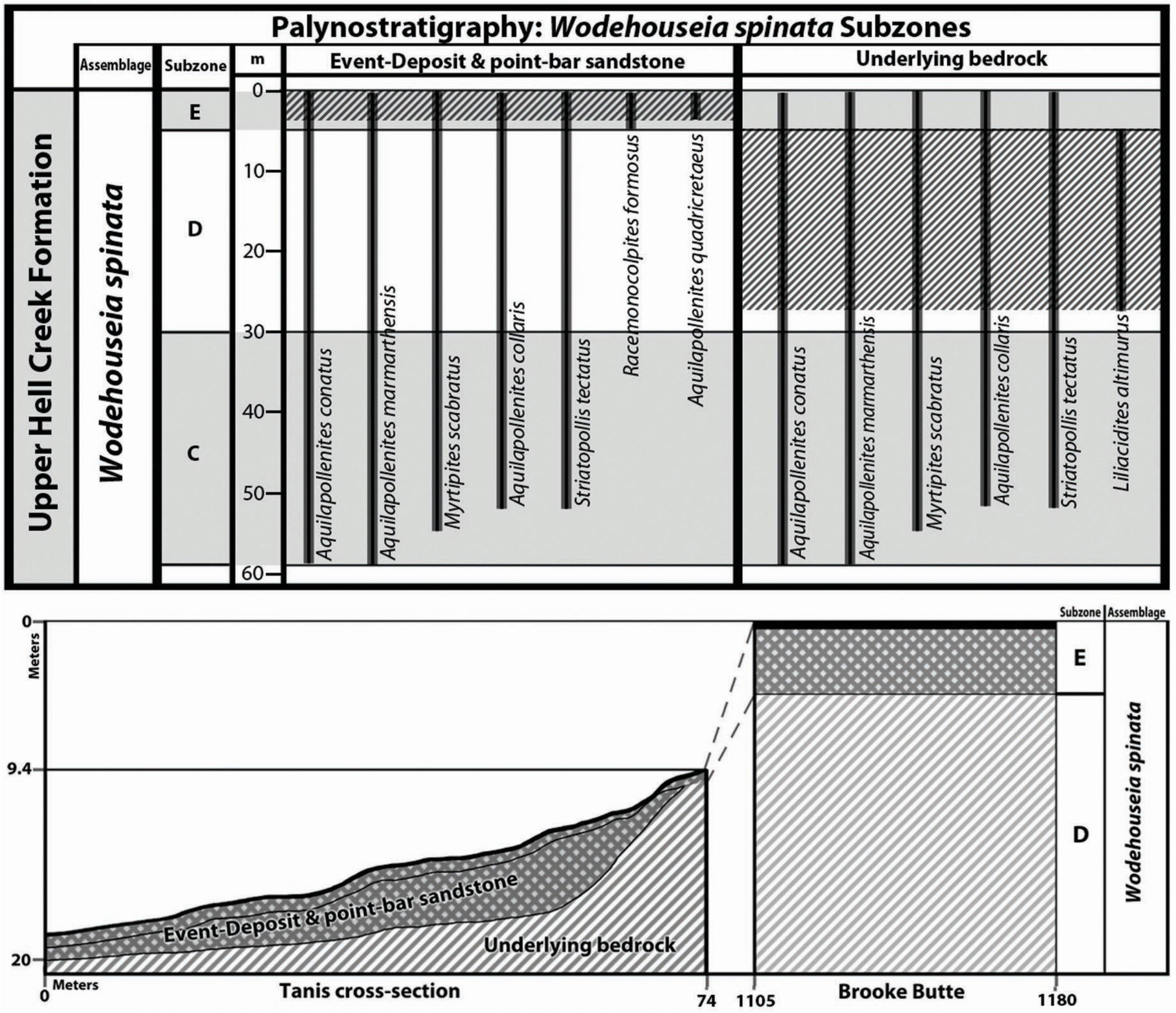

Fig. S21. Palynostratigraphy of the Tanis Event-Deposit, point-bar sandstone, and incised bedrock, showing some representative key palynological markers.

Palynology of the incised bedrock is compatible with the megafloral assessment, and indicates placement within the "D" subzone of the *Wodehouseia spinata* assemblage, sensu Nichols (***32***), which occupies the upper ~5 to 25 meters of the Hell Creek. Also compatible with the megafloral data, the combined Event-Deposit and point-bar sands are allied with much higher sediments, and belong to the "E" palynological subzone, which occupies the upper ~4 meters of the Hell Creek up to and including the KPg boundary. Together, the megafloral and palynological data indicate that the Event-Deposit and point-bar sands bear an uppermost, terminal-Cretaceous signature, consistent with an event precisely at the KPg boundary. Data from Nichols (***32***).

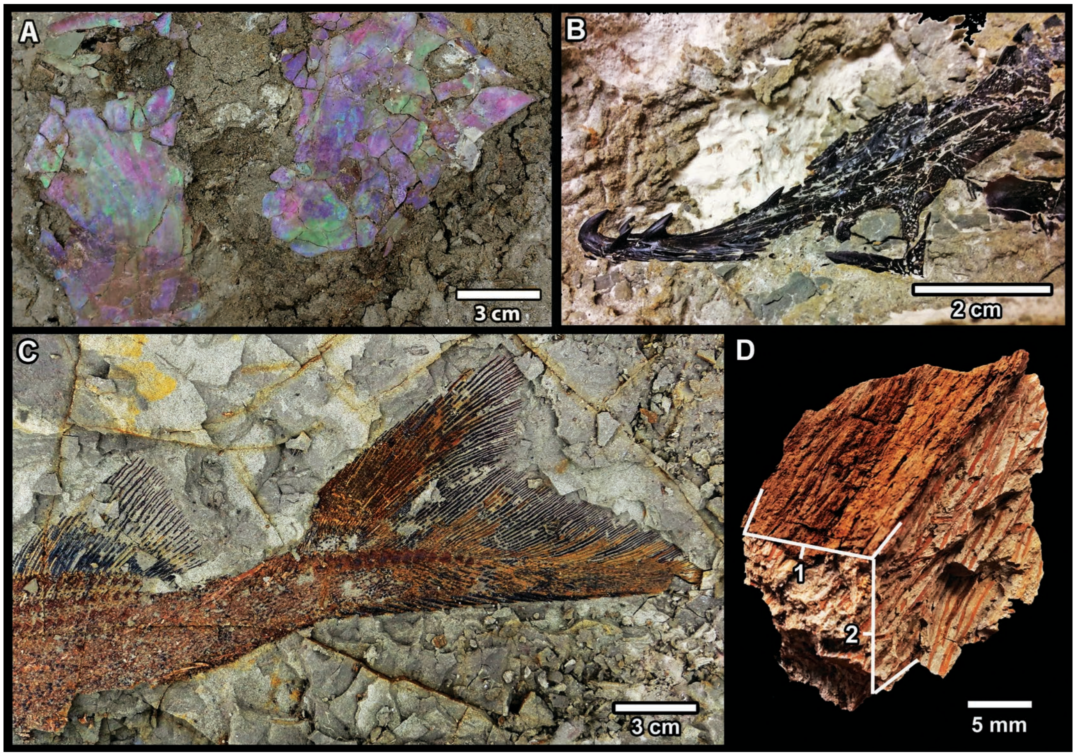

Fig. S22. Representative fossil preservation from the Tanis deposit.

(**A**), a large section of ammonite shell (FAU.DGS.ND.161.116); (**B**), magnified view of a sturgeon skull (FAU.DGS.ND.161.766.T); (**C**), magnified view of polyodontid tail (FAU.DGS.ND.161.688); and (**D**), a fragment of wood showing three-dimensional preservation that is common at Tanis but scarce elsewhere in the Hell Creek Formation, with well-preserved exterior surface (**D1**) and internal vascular microstructure throughout its interior (**D2**), rather than just a flat impression or lignified throughout as is common with other Hell Creek plants (FAU.DGS.ND.161.369.PH.T).

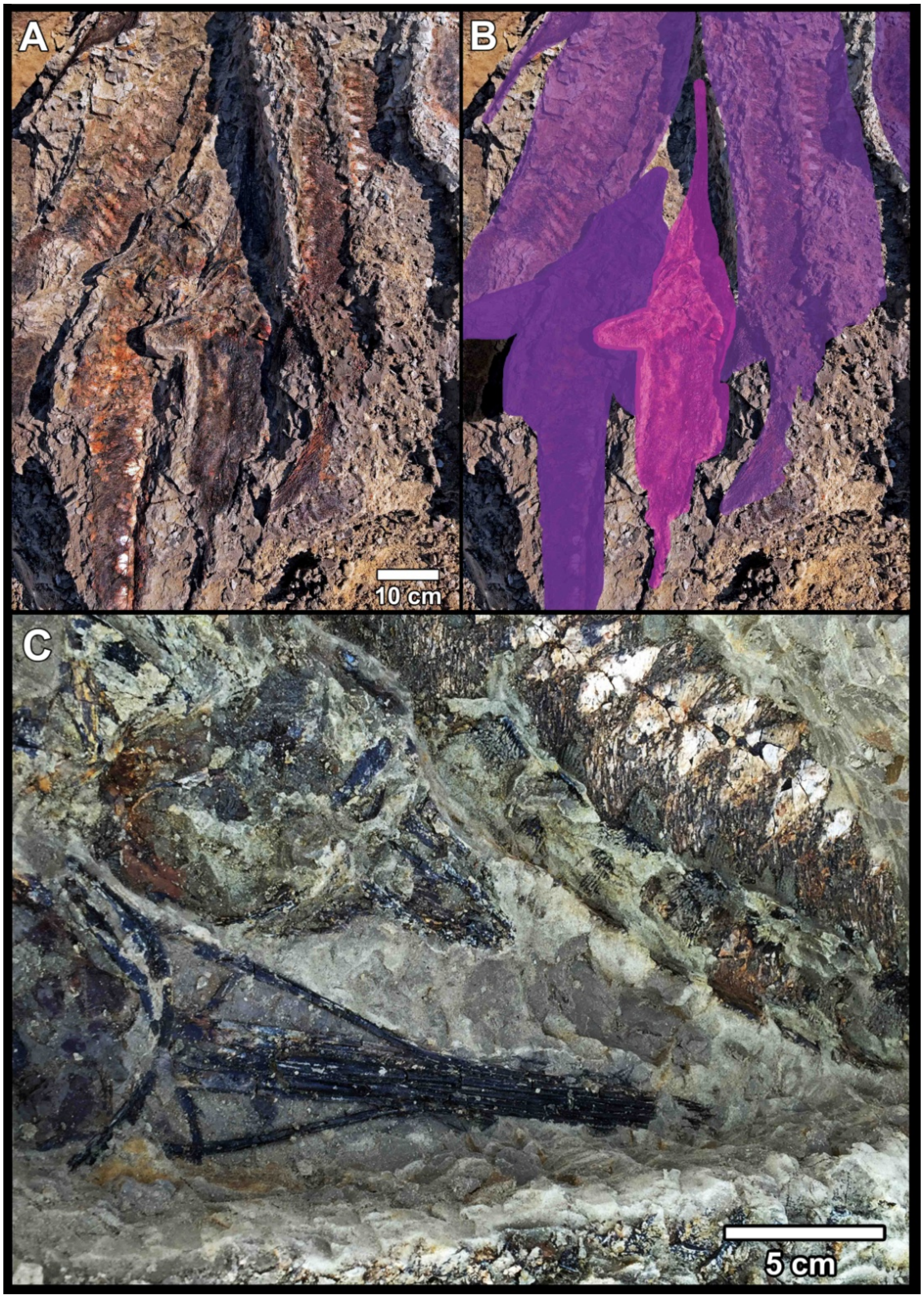

Fig. S23. Field photos of the Tanis Konservat-Lagerstätte.

(**A**), natural image; (**B**), individual carcasses colored for clarity, showing numerous acipenseriform fish carcasses and other organic debris clustered tightly together and oriented in the direction of flow. (**C**), magnified view of paddlefish and sturgeon.

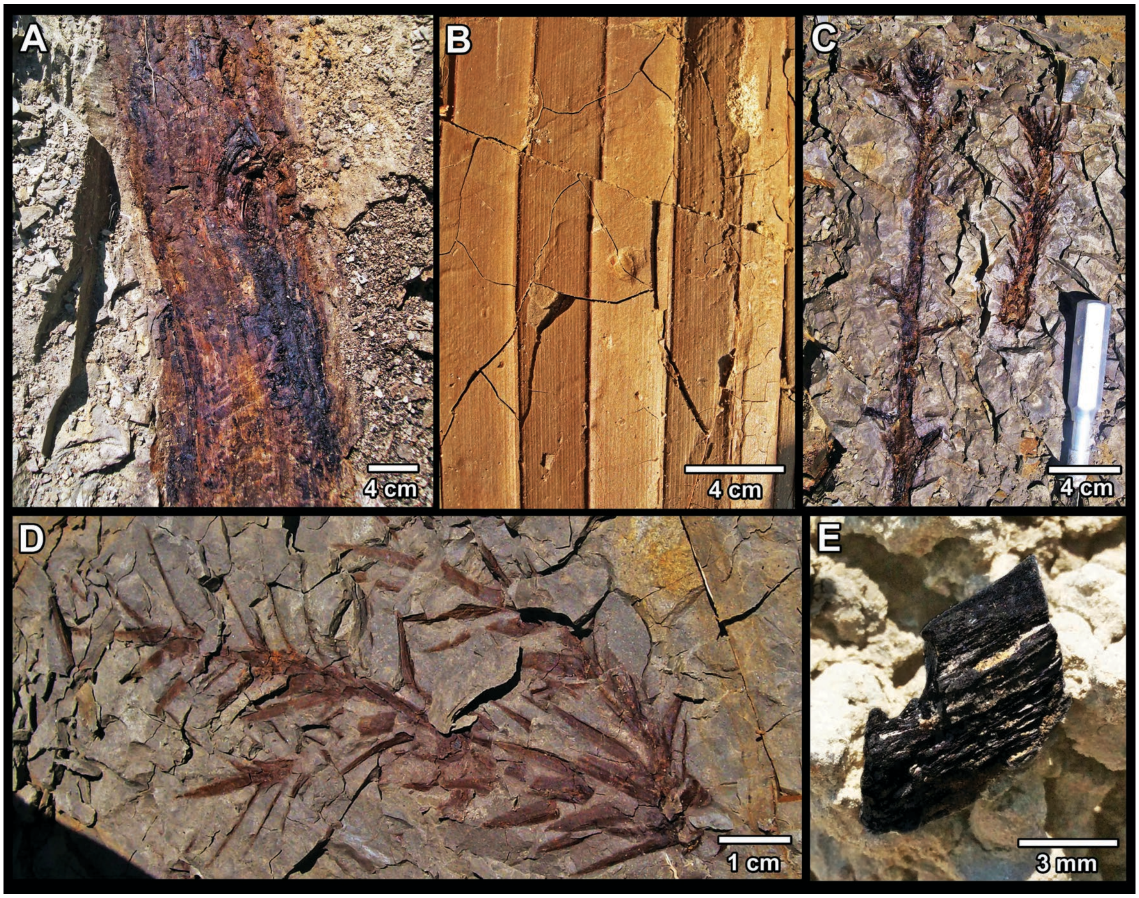

Fig. S24. A selection of plant material from Tanis (field photos).

(**A**), a large tree trunk; (**B**), a section of palm frond c.f. *Sabalites*; (**C**), branches of Cheirolepidiaceous foliage with needles attached three-dimensionally (FAU.DGS.ND.161.1011.T); (**D**), magnified view of Cheirolepidiaceous foliage (c.f. *Araucaria*) with three-dimensional needle arrangement (FAU.DGS.ND.161.1023.T); (**E**), highly angular piece of fossil charcoal, common at the site.

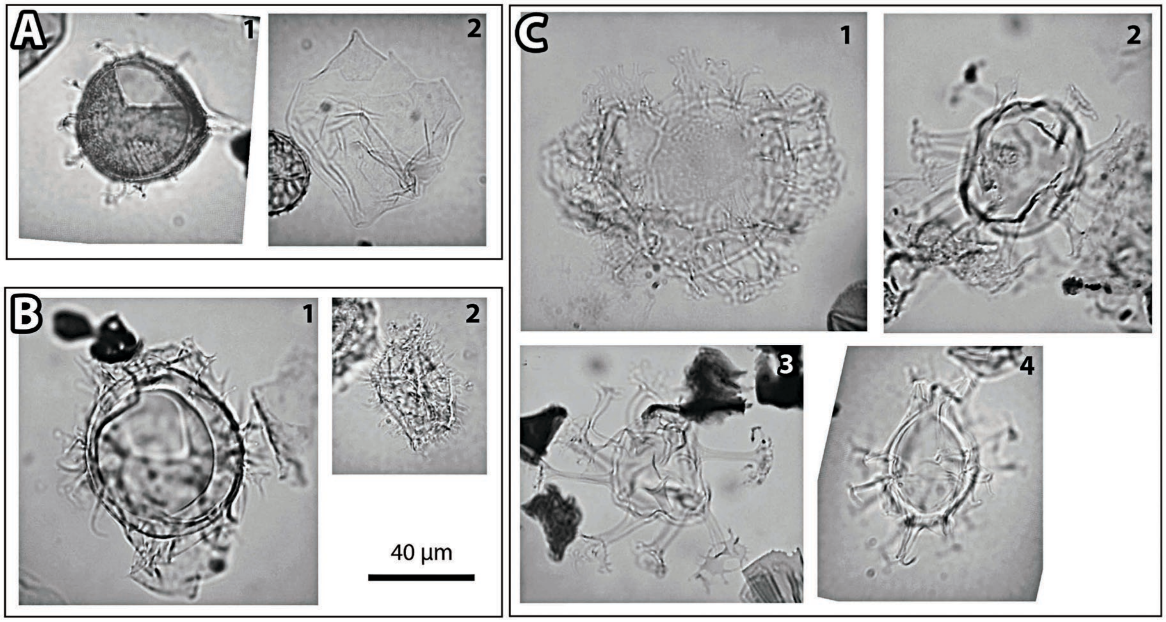

Fig. S25. Representative marine dinoflagellates from the Event-Deposit.

Taxa consist of contemporaneous latest-Maastrichtian markers such as *Pierceites pentagonus*(**A**1) and *Hafniasphaera septata* (**A**2) that are mixed with temporally broad taxa that span the Maastrichtian and hence are still compatible as contemporaneous (**C**), and older examples from the Late Campanian-Early Maastrichtian (**B**) that were churned and reworked from the bottom of the seaway during scouring/inundation and certainly pre-date the Tanis event. **A1**: *Hafniasphaera septata*; **A2**: *Pierceites pentagonus*; **B1**: *Triblastula utinensis*; **B2**: *Palaeohystrichophora infusoroides*. **C1**: *Areoligera* sp.; **C2**: *Hystrichosphaeridium tubiferum*; **C3**: *Oligosphaeridium pulcherrimum*; **C4**: *Spiniferites ramusus*. FAU.DGS.ND.161.1069.T.

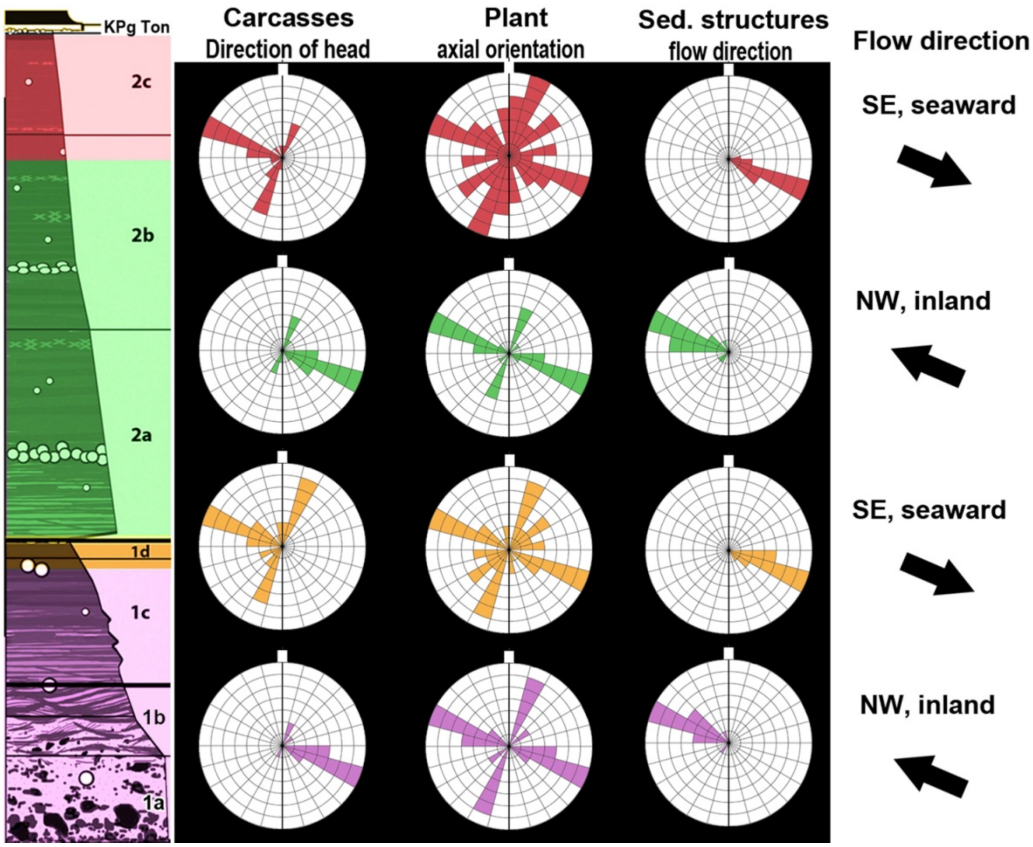

Figure S26. Paleocurrent direction within the Tanis sediment column, corroborated by multiple indicators.

Carcasses, plants, and sedimentological structures provide mutually reinforcing flow indication that documents at least two observable periods of 180° reversal, and a less prominent, secondary orientation of bio-debris 90° to flow, a characteristic of orientation via aqueous transport (***56***). (North is oriented to top of rose diagrams.)

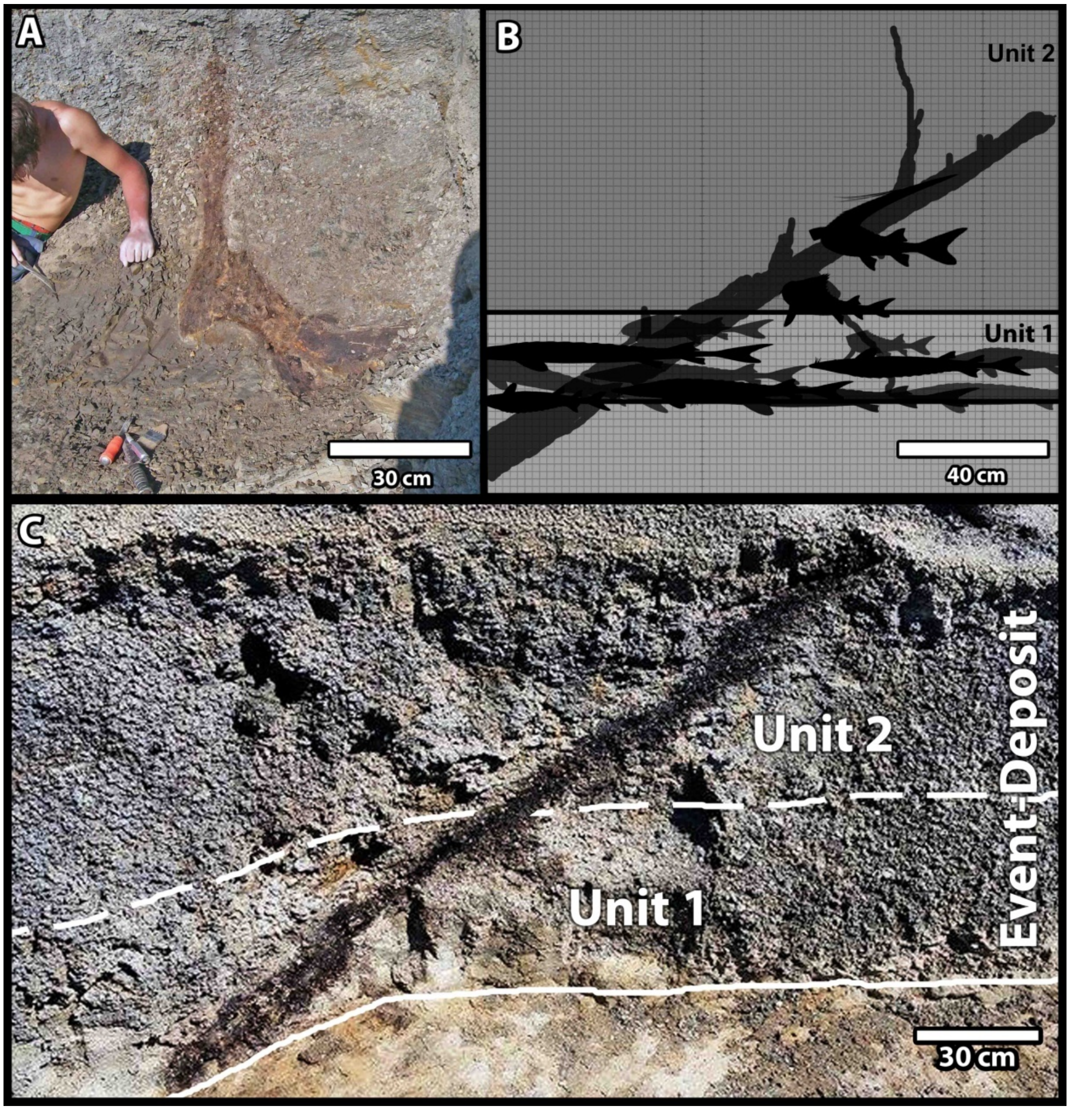

Fig. S27. Biological debris obliquely cross-cutting the sediment package at Tanis.

(**A**), the trunk of a gymnosperm tree oriented nearly vertically in the deposit. The trunk extended all the way to the upper margin of the Event-Deposit. (**B**), a vertical cross-section diagram of the trench at Tanis, showing a tree trunk and animal carcasses that are oriented by flow, many cross-cutting the entire deposit. (**C**), another tree in situ, showing an obliquely oriented trunk cross-cutting the deposit. Biological debris would have decayed rapidly and not extended through the entire vertical thickness of the deposit if it there was a long hiatus between Units 1 and 2.

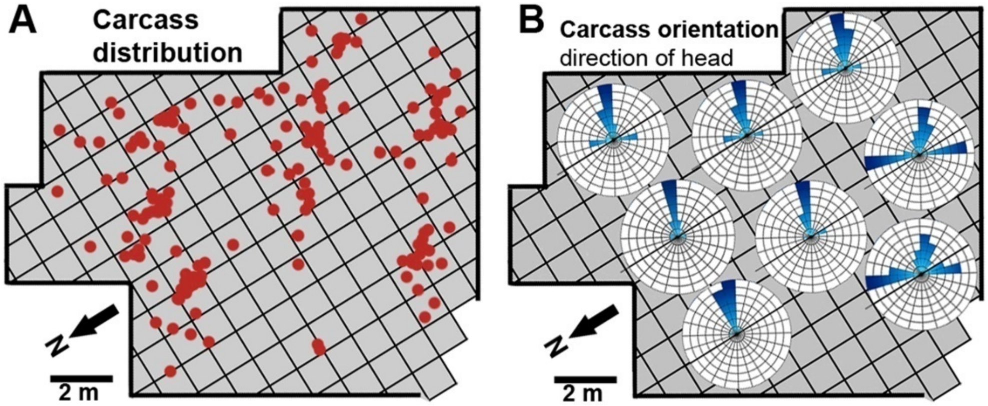

Figure S28. Carcass distribution and directional orientation of fish in the densest carcass-bearing portion of Tanis, Unit 2a.

(**A**), carcass distribution shows slightly elongated clustering along an ~east-west axis (carcasses represented by single dot). (**B**), data for orientation of fish (direction of head) plotted as rose diagrams, indicating a preferred inland-directed flow from ~east to west.

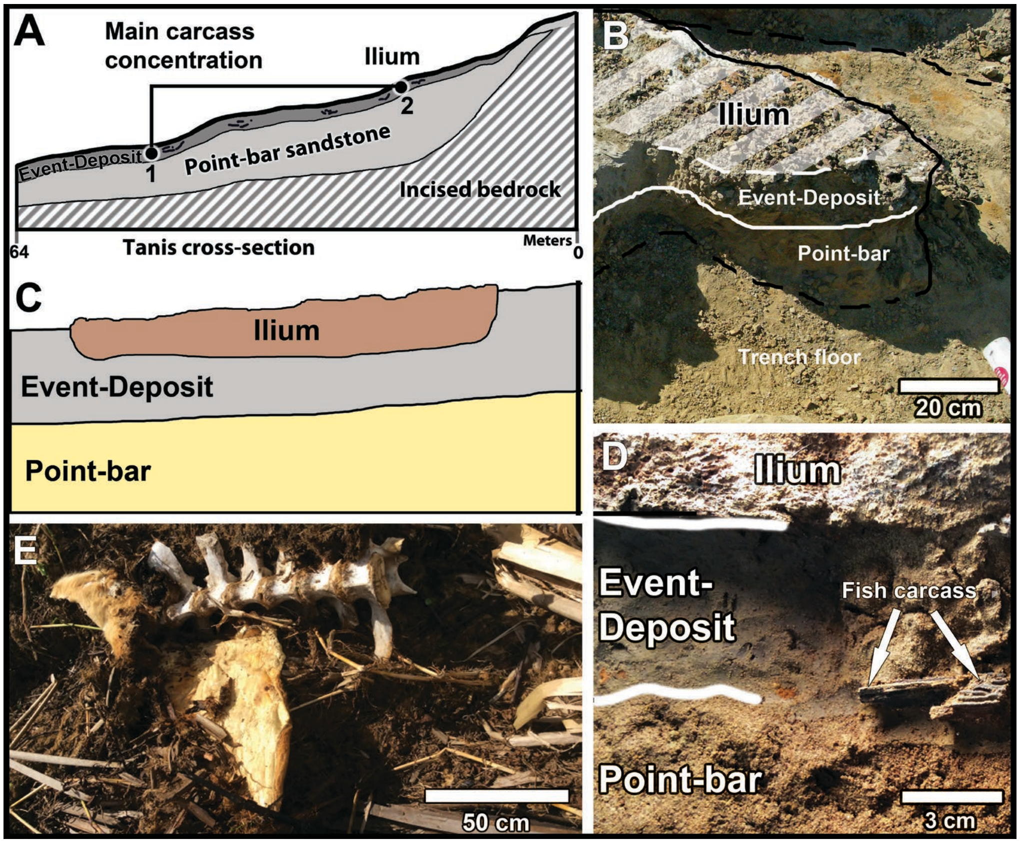

Figure S29. Context for the ceratopsian ilium at Tanis.

(**A**), cross-section schematic showing the relative location of the densest accumulation of carcasses (**A1**), and the ceratopsian ilium (**A2**; FAU.DGS.ND.161.1088.T) within the angled slope of the Event-Deposit. (**B**), field photo showing the ilium in context within the Event-Deposit, overlying the point-bar sands. (**C**), magnified schematic depicting the ilium's position within the Event-Deposit at the time of excavation. (**D**), a magnified field photo of the ilium in cross section as it was pedestaled during excavation, showing the ilium embedded in the gray Event-Deposit siltstone, separated from the tan point-bar sandstone beneath. (**E**), A string of articulated bovid vertebrae with attached swatches of sun-hardened skin, which were transported over 150 meters from their place of death by strong water currents and flooding from hurricane Irma at Lake Okeechobee, Florida, 2017. The bovid died 1.5 years prior, had since completely decayed and disarticulated, and the desiccated condition with high surface area likely aided in transport. This modern example, which demonstrates the ease by which completely or nearly skeletonized carcasses can be transported by flowing water, is a taphonomic analog of the ceratopsian ilium recovered at Tanis.

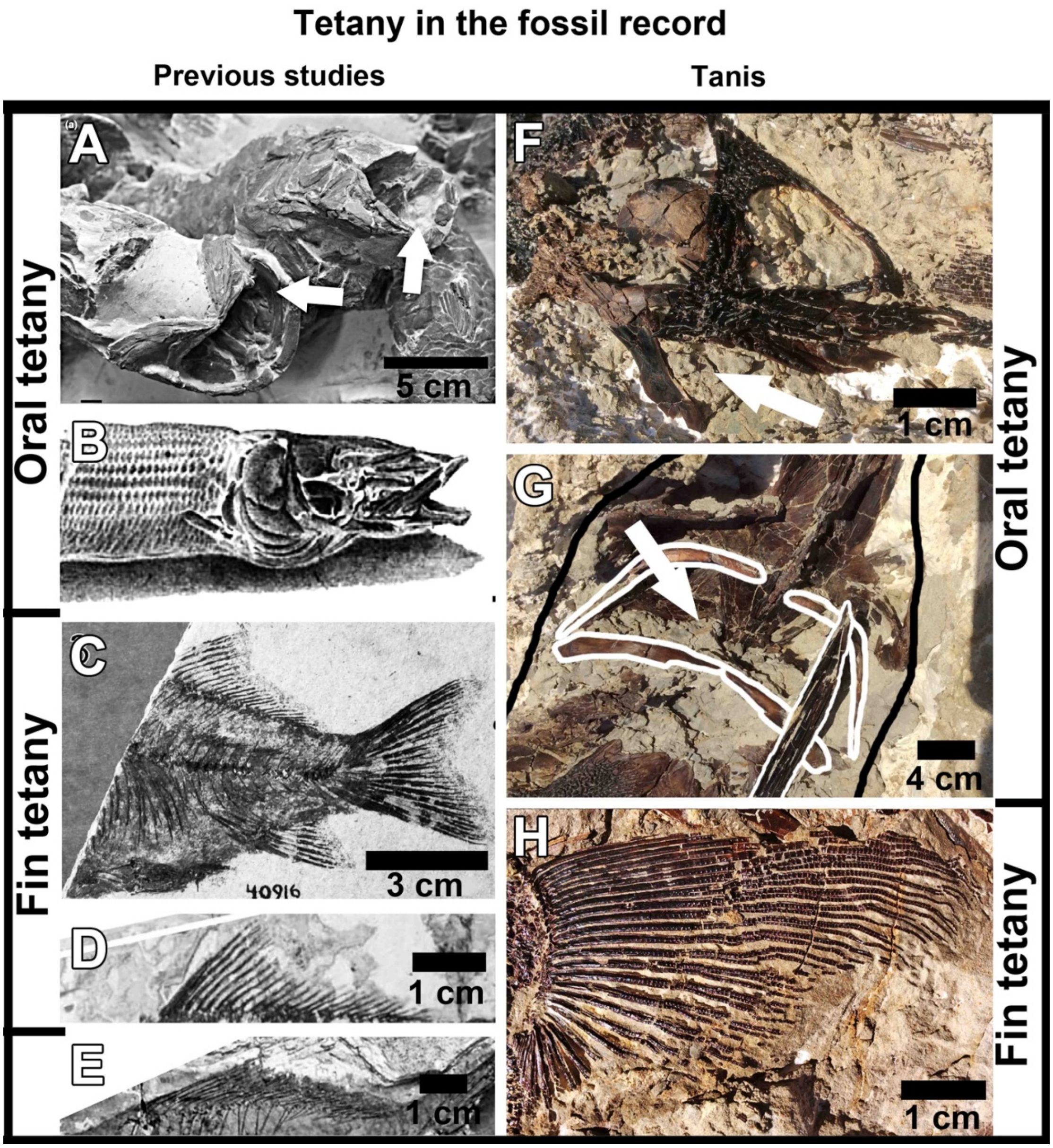

Figure S30. Examples of tetany in the fossil record.

(**A**, **B**) widely gaped mouths from fish in the London Clay (***63***) and (**C**, **D**) strongly extended, flabellate fin rays in fish from the Eocene of British Columbia (***62***), both diagnostic of tetany. The splayed fin arrangement contrasts with the default fin arrangement which is reclined close to the body, as seen in another fish from the Eocene (**E;** ***62***). (**F**, **G**), two sturgeon from Tanis, exhibiting widely gaped mouths, a common feature at the site ([**F**] is a right-lateral exposure, [**G**] is a ventral exposure; long narrow object in the lower right of [**G**] is a paddlefish rostrum lying parallel to the sturgeon body). (**H**), the hyperextended fin of a paddlefish from Tanis, comparing favorably with tetany in other fish fossils (e.g. **C**, **D**). The widespread signs of tetany among the carcasses at Tanis suggest that the same mode of death likely affected the entire assemblage of articulated carcasses, and thereby further supports that they represent a single death-event. (F): FAU.DGS.ND.161.547.T; (G, H): FAU.DGS.ND.161.115.T.

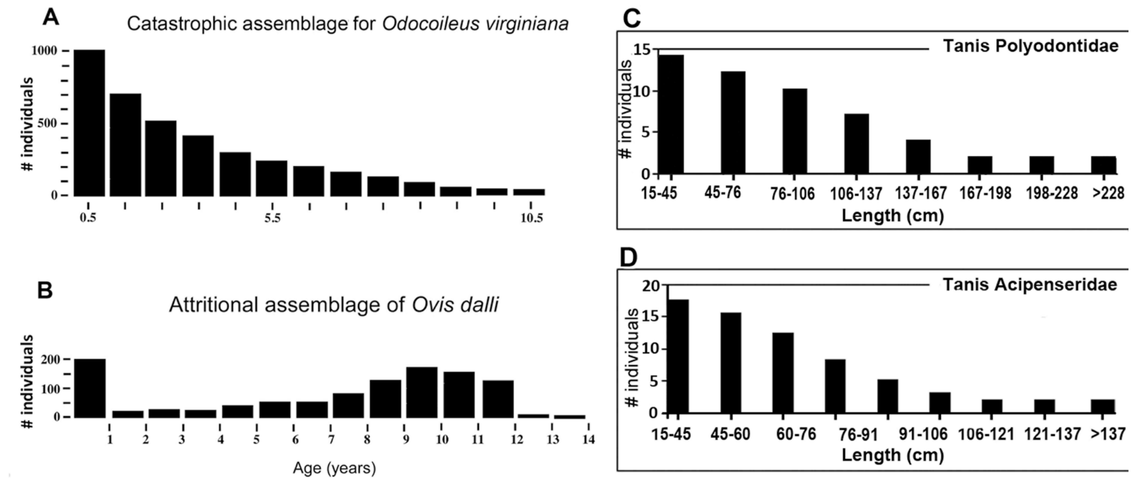

Figure S31. Tanis carcass size distribution compared to catastrophic and attritional assemblages.

Catastrophic assemblages representing a standing population (**A**) are compositionally distinct from attritional assemblages (**B**), which are bimodal and contain spikes in juvenile and older individuals with a depletion of intermediate stages. The acipenseriform fish at Tanis, the most abundantly represented carcasses, compare favorably with a catastrophic assemblage (**C, D**), reinforcing that the articulated carcasses at Tanis represent a catastrophic mass-death assemblage rather than an attritional assemblage. (**A**, **B**) reprinted with permission from ref. ***65***.

Table S1. LA-ICP-MS instrument parameters

LA-ICP instrument parameters		
**Parameter**	**Set A Dp sherules**	**Set B Dp V**
Laser wavelength	213 nm	213 nm
Ablation mode	Line (300µm)	Line (400µm)
Spot size	100 µm	100 µm
Power	100%E	40 %E
Laser energy	19.8 J/cm^2	2 J/cm^2
Repetition rate	10 Hz	5 Hz
Scan rate	10 µm/s	40 µm/s
Ar flow after the cell	~0.9 L/min	~0.9 L/min
He flow through cell	~0.9 L/min	~0.9 L/min
Calibrator reference standard materials	NIST 612 and BHVO	BHVO

Table S2. Analytical ^{40}Ar/^{39}Ar data and summary calculations

Table 2. ^{40}Ar/^{39}Ar analytical data											
**Location**	**36Ar(a) [fA]**	**37Ar(ca) [fA]**	**38Ar(cl) [fA]**	**39Ar(k) [fA]**	**40Ar(r) [fA]**	**Age ± 2σ (Ma)**		**40Ar(r) (%)**	**39Ar(k) [%]**	**K/Ca ± 2σ**	
**Tanis**											
TanisK21	0.0013777	4.66653	0.0000000	3.93119	29.2453	65.11	± 0.42	98.60	1.22	0.362	± 0.004
TanisK21	0.0071942	10.90314	0.0000000	6.09107	45.6539	65.59	± 0.37	95.50	1.89	0.240	± 0.002
TanisK21	0.0021434	2.99951	0.0000000	1.36669	10.0972	64.67	± 1.14	94.03	0.42	0.196	± 0.003
TanisK21	0.0035808	10.69334	0.0000000	4.29316	32.2376	65.71	± 0.41	96.78	1.33	0.173	± 0.002
TanisK21	0.0006872	4.42342	0.0000000	3.06652	22.8364	65.17	± 0.45	99.10	0.95	0.298	± 0.003
TanisK21	0.0083990	19.75239	0.0000000	13.84127	106.0811	67.04	± 0.17	97.68	4.29	0.301	± 0.003
TanisK21	0.0085288	18.47409	0.0000000	8.14920	61.3950	65.92	± 0.27	96.01	2.53	0.190	± 0.002
TanisK21	0.0195844	17.61742	0.0000000	7.51550	56.0849	65.31	± 0.29	90.55	2.33	0.183	± 0.002
TanisK21	0.0025072	6.63318	0.0000000	4.45919	33.3872	65.52	± 0.35	97.80	1.38	0.289	± 0.003
TanisK21	0.0059186	13.80736	0.0000000	7.18498	56.7055	69.00	± 0.29	96.97	2.23	0.224	± 0.002
TanisK21	0.0082082	19.70522	0.0000000	10.91835	83.0021	66.51	± 0.22	97.12	3.38	0.238	± 0.002
TanisK21	0.0067842	21.29167	0.0000000	9.62078	72.5582	65.99	± 0.22	97.27	2.98	0.194	± 0.002
TanisK21	0.0034912	8.46181	0.0025714	8.82237	68.0364	67.45	± 0.24	98.48	2.74	0.448	± 0.005
TanisK22	0.0249411	14.93381	0.0000000	6.81722	51.2859	65.83	± 0.29	87.31	2.11	0.196	± 0.002
TanisK22	0.0003995	2.78792	0.0000000	1.90149	14.3584	66.07	± 0.79	99.16	0.59	0.293	± 0.003
TanisK22	0.0020302	15.35280	0.0000000	7.32024	54.9113	65.64	± 0.25	98.90	2.27	0.205	± 0.002
TanisK22	0.0009866	4.14174	0.0000000	2.91032	21.7412	65.37	± 0.61	98.65	0.90	0.302	± 0.004
TanisK22	0.0014823	2.93203	0.0000000	1.17973	8.9327	66.25	± 1.60	95.27	0.37	0.173	± 0.003
TanisK22	0.0134280	30.86103	0.0000000	13.79650	103.7281	65.79	± 0.19	96.27	4.28	0.192	± 0.002
TanisK22	0.0045102	13.99845	0.0000000	9.60424	72.6283	66.16	± 0.24	98.17	2.98	0.295	± 0.003
TanisK22	0.0025755	12.24408	0.0000000	7.02599	52.5938	65.50	± 0.28	98.55	2.18	0.247	± 0.003
TanisK22	0.0328623	22.08154	0.0000000	11.53174	86.6596	65.76	± 0.25	89.82	3.58	0.225	± 0.002
TanisK22	0.0075477	25.68035	0.0000000	13.55629	101.9025	65.77	± 0.22	97.83	4.20	0.227	± 0.002
TanisK22	0.0058619	19.73945	0.0000000	11.69700	88.0008	65.83	± 0.20	98.04	3.63	0.255	± 0.003
TanisK22	0.0101203	24.20409	0.0000000	13.67736	103.0137	65.90	± 0.20	97.14	4.24	0.243	± 0.002
TanisK22	0.0081406	15.34155	0.0000000	10.67429	80.3712	65.88	± 0.22	97.05	3.31	0.299	± 0.003
TanisK22	0.0040965	21.32629	0.0000000	12.52252	94.2231	65.84	± 0.18	98.71	3.88	0.252	± 0.003
TanisK23	0.0043091	7.87991	0.0000000	4.12157	31.0355	65.89	± 0.42	96.01	1.28	0.225	± 0.002
TanisK23	0.0047641	8.13221	0.0000000	5.70177	42.7308	65.58	± 0.41	96.77	1.77	0.301	± 0.003
TanisK23	0.0059873	17.19609	0.0000000	8.57175	64.4706	65.81	± 0.30	97.29	2.66	0.214	± 0.002
TanisK23	0.0028679	7.49279	0.0000000	3.46070	25.6838	64.95	± 0.52	96.76	1.07	0.199	± 0.002
TanisK23	0.0029442	6.28042	0.0000000	4.28499	32.2067	65.77	± 0.41	97.33	1.33	0.293	± 0.003
TanisK23	0.0025904	6.06488	0.0000000	4.03591	30.0710	65.21	± 0.50	97.48	1.25	0.286	± 0.003
TanisK23	0.0075933	12.81009	0.0000000	7.77343	58.8007	66.18	± 0.30	96.28	2.41	0.261	± 0.003

TanisK23	0.0043956	14.87080	0.0000000	8.03962	60.4648	65.81	± 0.27	97.86	2.49	0.232	± 0.002
TanisK23	0.0034701	13.68989	0.0000000	6.96021	52.5799	66.10	± 0.28	98.06	2.16	0.219	± 0.002
TanisK23	0.0078496	18.35901	0.0000000	12.35767	92.5052	65.50	± 0.19	97.52	3.83	0.289	± 0.003
TanisK23	0.0092018	21.08975	0.0000000	10.48366	78.8660	65.82	± 0.26	96.62	3.25	0.214	± 0.002
TanisK23	0.0055881	18.90700	0.0000000	8.13584	61.1529	65.77	± 0.27	97.33	2.52	0.185	± 0.002
TanisK23	0.0070581	21.30749	0.0000000	10.73073	80.2022	65.41	± 0.21	97.43	3.33	0.217	± 0.002
TanisK23	0.0089408	21.74696	0.0000000	11.32789	85.3691	65.94	± 0.24	96.96	3.51	0.224	± 0.002
TanisK23	0.0013830	5.97016	0.0000000	3.09579	23.8188	67.29	± 0.58	98.29	0.96	0.223	± 0.003

Beloc

BelocK24JS	0.0005632	2.10764	0.0000000	1.36999	10.16148	64.92	± 1.22	98.36	1.25	0.280	± 0.004
BelocK24JS	0.0012318	1.65843	0.0000000	0.85896	6.30694	64.27	± 2.06	94.48	0.78	0.223	± 0.005
BelocK24JS	0.0008483	2.66914	0.0000000	1.40662	10.57624	65.79	± 1.10	97.65	1.28	0.227	± 0.004
BelocK24JS	0.0015143	5.45463	0.0000000	3.75139	28.24900	65.89	± 0.44	98.41	3.42	0.296	± 0.003
BelocK24JS	0.0024372	5.83853	0.0000000	3.62226	27.39666	66.17	± 0.48	97.40	3.30	0.267	± 0.003
BelocK24JS	0.0421332	16.78958	0.0000000	8.57468	65.06329	66.38	± 0.33	83.79	7.82	0.220	± 0.002
BelocK24JS	0.0294187	19.43397	0.0000000	11.18146	84.30735	65.97	± 0.26	90.56	10.20	0.247	± 0.002
BelocK24JS	0.0313084	17.58916	0.0000000	8.65185	64.98822	65.73	± 0.27	87.42	7.89	0.212	± 0.002
BelocK24JS	0.0106873	17.42231	0.0000000	10.13591	75.95530	65.57	± 0.26	95.96	9.24	0.250	± 0.003
BelocK24JS	0.0031454	8.96590	0.0000000	4.94130	37.32870	66.10	± 0.29	97.54	4.51	0.237	± 0.003
BelocK24JS	0.0079565	23.65211	0.0000000	10.71716	80.72958	65.91	± 0.23	97.13	9.77	0.195	± 0.002
BelocK24JS	0.0061082	9.93386	0.0000000	6.48028	49.16378	66.37	± 0.32	96.41	5.91	0.281	± 0.003
BelocK24JS	0.0084385	5.57106	0.0000000	2.87172	21.69704	66.10	± 0.56	89.59	2.62	0.222	± 0.003
BelocK25Rd	0.0548150	31.00072	0.0000000	13.80894	103.4771	65.57	± 0.25	86.34	12.59	0.192	± 0.002
BelocK25Rd	0.0043456	7.50991	0.0000000	5.15389	39.05317	66.29	± 0.36	96.77	4.70	0.295	± 0.003
BelocK25Rd	0.1169386	16.99815	0.0000000	7.62985	58.13438	66.65	± 0.54	62.47	6.96	0.193	± 0.002
BelocK25Rd	0.0059533	16.90940	0.0000000	8.50369	64.25191	66.11	± 0.25	97.30	7.75	0.216	± 0.002

IrZ control

IrZK26	0.0008437	0.1085604	0.0000000	9.03207	68.1066	0.0048539	± 0.0000168	99.62	2.27	36	± 1
IrZK26	0.0002969	0.0688473	0.0000000	11.14263	83.6788	0.0048738	± 0.0000144	99.88	2.80	70	± 3
IrZK26	0.0026414	0.1143508	0.0000000	15.85072	118.5418	0.0048941	± 0.0000135	99.33	3.98	60	± 2
IrZK26	0.0059930	0.0959257	0.0000000	15.64645	117.9202	0.0048565	± 0.0000126	98.49	3.93	70	± 2
IrZK26	0.0053694	0.0773618	0.0000000	9.62923	72.4595	0.0048639	± 0.0000164	97.82	2.42	54	± 2
IrZK26	0.0047746	0.1001133	0.0000000	14.02764	104.7429	0.0049018	± 0.0000144	98.65	3.52	60	± 2
IrZK26	0.0006762	0.0692957	0.0000000	10.73102	80.7069	0.0048666	± 0.0000156	99.74	2.69	67	± 3
IrZK26	0.0003432	0.0883686	0.0000000	13.93770	104.7495	0.0048700	± 0.0000137	100.09	3.50	68	± 3
IrZK26	0.0038143	0.0988366	0.0000000	12.60032	94.8298	0.0048633	± 0.0000170	98.80	3.16	55	± 2
IrZK26	0.0013575	0.1045992	0.0000000	19.20494	144.3520	0.0048695	± 0.0000147	99.71	4.82	79	± 3
IrZK26	0.0021087	0.1902937	0.0000000	30.78888	231.6975	0.0048637	± 0.0000111	99.72	7.73	70	± 4
IrZK26	0.0055221	0.1810808	0.0000000	28.22038	212.8471	0.0048527	± 0.0000124	99.22	7.09	67	± 3
IrZK26	0.0048441	0.1549981	0.0000000	27.45323	205.8618	0.0048810	± 0.0000114	99.29	6.89	76	± 3
IrZK26	0.0169697	0.1779599	0.0000000	27.41899	206.3455	0.0048635	± 0.0000117	97.59	6.89	66	± 2
IrZK26	0.0033801	0.1976523	0.0000000	30.92059	232.3459	0.0048708	± 0.0000116	99.56	7.76	67	± 3
IrZK26	0.0033746	0.1486873	0.0000000	24.97244	187.9263	0.0048637	± 0.0000116	99.46	6.27	72	± 3
IrZK26	0.0056352	0.2059933	0.0000000	28.09265	211.3136	0.0048658	± 0.0000119	99.20	7.05	59	± 2
IrZK26	0.0044082	0.1685866	0.0000000	23.59528	177.2536	0.0048722	± 0.0000125	99.25	5.93	60	± 2
IrZK26	0.0032559	0.1649919	0.0000000	24.66318	185.9106	0.0048555	± 0.0000113	99.47	6.19	64	± 2
IrZK26	0.0050445	0.1353050	0.0000000	20.27686	152.3629	0.0048709	± 0.0000137	99.01	5.09	64	± 3

Summary		$^{40}Ar/^{39}Ar_k$ ± 2σ		Age (Ma) ± 2σ		MSWD	N	NTotal
Tek Tanis		7.51482	± 0.00937	65.76	± 0.15	3.10	36	42
Tek Beloc		7.53825	± 0.01735	65.96	± 0.20	3.20	14	17
IrZ (standard)		7.52431	± 0.00491			0.99	17	20

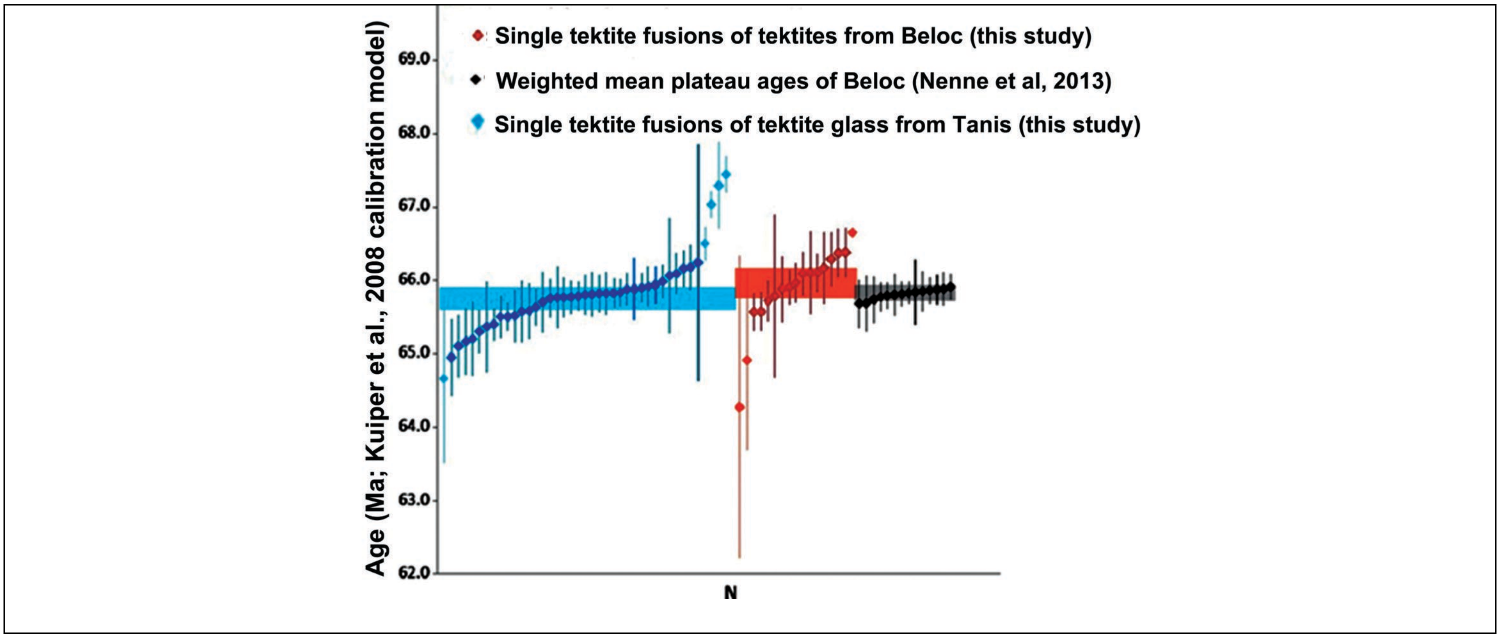
Single tektite fusions of tektites from Beloc (this study)
Weighted mean plateau ages of Beloc (Nenne et al, 2013)
Single tektite fusions of tektite glass from Tanis (this study)
Age (Ma; Kuiper et al., 2008 calibration model)
69.0
68.0
67.0
66.0
65.0
64.0
63.0
62.0
N

Table S3. Geochemistry of diagenetically altered spherules from Tanis.
Sph-EV-DP = spherule from Event-Deposit; Sph-Tonst = spherule from tonstein.

Locality	Brazos	La Sierrita	Haiti	Beloc Haiti	El Mimbral	Mud Buttes	Tanis	Tanis
Material	Spherule	Spherule	Spherule	Spherule	Spherule	Spherule	Sph-EV-DP	Sph-Tonst
References	21	20	This study	17	17	This study	This study	This study
	(wt %)	(wt %)	(wt %)	(wt %)	(wt %)	(wt %)	(wt %)	(wt %)
SiO_2	53.87-60.83	50.12-50.23	52.94-54.92	57.6	41.6	60.20-61-78	60.77-62.30	59.74-60.91
TiO_2	0.06-1.26	0.15-0.19	1.27	0.84	0.09	0.13-0.32	0.25-0.40	0.90-1.12
Al_2O_3	22.21-24.40	29.15-29.55	11.72-11.78	16.3	15	10.94-11.31	11.34-12.27	12.20-12.64
FeO	0.63-4.07	1.49-1.88	6.88	6.76	17.9	3.30-3.57	3.86-4.65	4.53-4.80
MgO	2.76-4.56	2.14-2.35	5.62-5.63	5.61	9.24	6.00-6.17	6.18-6.45	5.85-6.07
CaO	0.03-0.10	0.56-0.57	3.15	2.59	2.75	0.50-0.53	0.72-0.78	0.61-0.69
Na_2O	0.01-0.09	0.1	0.04	0.03	0.05	1.19-1.25	1.34-1.37	0.98-1.25
K_2O	0.90-1.25	7.14-7.25	0.23	0.34	0.39	0.18-0.24	0.20-0.30	0.32-0.39
MnO	N/D	N/D	0.009	<lod	<LOD	0.00-0.07	0.00-0.02	0.00-0.04
Cr_2O_3	N/D	N/D	0	<lod	<LOD	0.00-0.01	0.00-0.01	0.01-0.03
NiO_2	N/D	N/D	0.02	N/D	N/D	0.00-0.01	0.02-0.03	0
P_2O_5	N/D	N/D	0	N/D	N/D	0.01-0.11	0.00-0.02	0.00-0.01
	(ppm)	(ppm)	(ppm)	(ppm)	(ppm)	(ppm)	(ppm)	(ppm)
Sc	N/D	N/D	20	22.1	18.1	17	21	15
V	N/D	N/D	191	87.6	329	246	91	74
Co	N/D	N/D	14	6.04	9.5	19	6	12
Cu	N/D	N/D	80	64.5	27.9	30	40	40
Zn	N/D	N/D	90	287	82.8	100	70	70
Ga	N/D	N/D	8	11.3	15.2	4	10	8
As	N/D	N/D	15	<lod	<lod	71	<5	<5
Rb	N/D	N/D	7	13	12.3	7	6	5
Sr	N/D	N/D	85	93	61.3	93	77	90
Y	N/D	N/D	8.1	2.75	2.8	44	2.2	3
Zr	N/D	N/D	155	144	46.3	202	190	118
Nb	N/D	N/D	< 0.2	4.03	14.1	1	2.2	1.8
Mo	N/D	N/D	< 2	N/D	N/D	<2	<2	<2
Ag	N/D	N/D	< 0.5	N/D	N/D	<0.5	<0.5	<0.5
Sn	N/D	N/D	3	N/D	N/D	4	2	1
Sb	N/D	N/D	43.6	N/D	N/D	36.7	2.7	8
Cs	N/D	N/D	0.62	0.71	<lod	3.5	0.67	0.4
Ba	N/D	N/D	321	1966	59.6	302	351	396
La	N/D	N/D	15.6	1.95	0.63	15.6	4.85	3.49
Ce	N/D	N/D	18	3.74	1.85	31	9.54	0.07
Pr	N/D	N/D	18	0.54	0.32	3.12	0.82	0.79
Nd	N/D	N/D	8.85	2.08	1.1	10.7	3.06	3.54
Sm	N/D	N/D	0.99	0.56	<lod	1.4	0.82	0.62
Eu	N/D	N/D	0.225	<lod	<lod	4.2	0.201	0.188
Gd	N/D	N/D	0.78	<lod	<lod	1.38	0.61	0.64
Tb	N/D	N/D	0.37	<lod	<lod	0.23	0.04	0.1
Dy	N/D	N/D	0.75	0.6	0.74	1.12	0.47	0.49
Ho	N/D	N/D	0.12	<lod	<lod	0.18	0.1	0.9
Er	N/D	N/D	0.37	0.34	0.96	0.49	0.3	0.22
Tm	N/D	N/D	0.1	<lod	<lod	0.69	0.04	0.034
Yb	N/D	N/D	0.37	0.4	0.71	0.64	0.19	0.29
Lu	N/D	N/D	0.057	<lod	<lod	0.077	0.026	0.042
Hf	N/D	N/D	2.5	4.27	1.42	4.5	5.5	0.39
Ta	N/D	N/D	0.19	6.47	N/D	0.02	0.65	0.34
W	N/D	N/D	10.4	<lod	0.69	13.1	92.9	4.8
Pb	N/D	N/D	11	5.23	88.2	<5	21	7
Th	N/D	N/D	12	5.8	1.14	18.3	5.31	4.43
U	N/D	N/D	< 0.2	<lod	3.93	1.32	0.72	0.35
Cr	N/D	N/D	40	36.2	44.1	120	70	30
Ni	N/D	N/D	<20	78	29.4	40	40	90

Table S4. Geochemistry of unaltered Chicxulub impact glass from Tanis.

GL-Amber = glass spherule from amber; GL-EV-DP = glass from Event-Deposit; GL-Tonst = glass from tonstein.

Locality	El Mimbral	Gorgonilla	El Mimbral	Beloc Haiti	Haiti	Mud Buttes	Tanis	Tanis	Tanis
Material	Relict glass	Black glass	Black glass	Black glass	Black glass	Relict glass	GL-Amber	GL-EV-DP	GL-Tonst
References	25	24	22	22	Thus study	This study	This study	This study	This study
	(wt %)	(wt %)	(wt %)	(wt %)	(wt %)	(wt %)	(wt %)	(wt %)	(wt %)
SiO_2	64.6	46.43-68.41	61.3	64.2	65.08-66.78	63.60-64.76	64.4	65.14-65.63	63.82-66.03
TiO_2	0.6	.42-.68	0.73	0.59	0.69-0.71	0.55-0.70	0.61	0.56-0.61	0.50-0.72
Al_2O_3	14.5	8.69-15.85	15.3	14.4	15.31-15.68	15.04-15.37	15.33	15.13-15.50	15.06-15.96
FeO	4.9	3.68-6.45	6.06	5.15	4.31-4.93	4.47-4.71	4.61	4.41-4.83	4.49-5.19
MgO	2.7	1.88-4.84	3.47	2.75	2.64-2.74	2.52-2.92	2.63	2.43-2.75	2.57-2.74
CaO	5.5	5.45-30.26	6.76	6.45	4.84-5.21	5.67-6.01	5.88	5.29-5.39	5.14-5.93
Na_2O	2.8	.97-4	3.21	3.41	2.04-2.72	2.36-2.82	2.88	2.10-3.40	2.92-3.07
K_2O	1.7	.28-1.87	1.41	1.59	1.50-1.71	1.61-1.73	1.62	1.36-1.84	1.62-1.79
MnO	0.1	.12-.16	0.14	0.13	0.14-0.22	0.15-0.31	0.14	0.08-0.22	0.13-0.34
Cr_2O_3	N/D	N/D	<lod	<LOD	0.00-0.02	0.00-0.01	0.01	0.00-0.01	0.00-0.01
NiO_2	N/D	N/D	N/D	N/D	0.00-0.01	0.0-0.02	0.01	<0.01	0.00-0.01
	(ppm)	(ppm)	(ppm)	(ppm)	(ppm)	(ppm)	(ppm)	(ppm)	(ppm)
Sc	N/D	N/D	26.7	20.2	15.09	15	15.8	14	15
V	N/D	N/D	175	110	70.87	107	109	108	126
Co	N/D	N/D	17.9	9.5	11.63	10	9.8	10	12
Cu	N/D	N/D	35.3	27.5	15.15	30	28	30	66
Zn	N/D	N/D	74.4	82.8	11.42	<30	41	40	120
Ga	N/D	N/D	17.8	15.2	10.2	10	11.2	11	9
As	N/D	N/D	>lod	>lod	<lod	36	32	57	72
Rb	N/D	N/D	41.6	45.3	49.45	43	44	38	40
Sr	N/D	N/D	380	449	218.63	369	322	314	359
Y	N/D	N/D	25.7	22.3	19.58	24	21.2	19.9	22.1
Zr	N/D	N/D	130	132	108.26	142	132	145	177
Nb	N/D	N/D	6.1	6	6.74	6.76	6.71	6.74	5.5
Mo	N/D	N/D	N/D	N/D	4	3	3	< 2	6
Ag	N/D	N/D	N/D	N/D	<lod	< 0.5	<0.5	< 0.5	< 0.5
Sn	N/D	N/D	N/D	N/D	3.5	< 0.1	<0.1	< 0.1	< 0.1
Sb	N/D	N/D	N/D	N/D	142	3	3	2	6
Cs	N/D	N/D	1.13	0.99	0.7	84.2	91.2	163	132
Ba	N/D	N/D	416	558	455.51	0.6	84	0.6	0.8
La	N/D	N/D	19.2	19.6	17.93	447	225	461	461
Ce	N/D	N/D	38.8	41.2	41.06	21.6	37.4	20.7	25.1
Pr	N/D	N/D	4.72	4.72	5.22	46.2	28.8	41.1	45.1
Nd	N/D	N/D	15.4	18.4	17.94	5.38	6.1	4.78	5.19
Sm	N/D	N/D	4.9	4.38	4.52	16.8	12.9	14.8	18.2
Eu	N/D	N/D	1.22	1.14	1.17	5.33	3.72	3.17	4.17
Gd	N/D	N/D	5.14	4.07	3.64	1.23	2.23	0.724	1.15
Tb	N/D	N/D	<lod	<lod	0.437	4.17	2.78	3.88	3.31
Dy	N/D	N/D	5.2	4.16	3.72	0.66	0.88	0.59	0.65
Ho	N/D	N/D	1	0.8	0.71	3.98	3.22	3.57	3.96
Er	N/D	N/D	2	2.45	1.98	0.73	0.89	0.69	0.78
Tm	N/D	N/D	0.45	0.39	0.334	2.07	0.98	1.89	2.22
Yb	N/D	N/D	2.91	2.7	1.95	0.433	0.59	0.404	0.423
Lu	N/D	N/D	0.56	0.4	0.42	3.1	3.3	2.9	3.1
Hf	N/D	N/D	4.57	3.96	2.85	0.495	0.477	0.484	0.499
Ta	N/D	N/D	0.41	0.42	0.23	2.6	2.9	3	3.9
W	N/D	N/D	0.79	0.69	9.2	0.49	0.38	0.22	0.25
K	N/D	N/D	N/D	N/D	1.29	7.8	9.6	11.2	4.2
Pb	N/D	N/D	3.29	4.38	14	12	14	13	15
Th	N/D	N/D	7.33	6.33	5.36	5.89	6.12	6	5.14
U	N/D	N/D	1.85	1.4	1.45	1.35	1.38	1.26	1.81
Cr	N/D	N/D	104	44.1	23	20	21	20	140
Ni	N/D	N/D	31.7	29.4	47	30	38	70	30

Table S5. Refractive index, water content, and iridium concentratons for Tanis.

EV-DP = glass from Event-Deposit; Tonst = glass from tonstein band; P2 overlying Tonst = Paleogene above tonstein; Ev-2 = Event-Deposit immediately beneath tonstein; Ev-1 = lowermost Event-Deposit.

Locality	**El Mimbral**	**Haiti**	**Haiti**	**Mud Buttes**	**Tanis**	**Tanis**
Material	**Relict glass**	**Relict glass**	**Relict glass**	**Relict glass**	**Glass- EV-DP**	**Glass- Tonst**
References	**26**	**23**	**This study**	**This study**	**This study**	**This study**
**Index of refraction**	1.51-1.55	1.51-1.55	1.52-1.54	1.53-1.56	1.52-1.54	1.53-1.55
**Water content**	0.05%	nd	0.04%	0.03%	0.02%	0.03%
**Locality**	**Tanis**	**Tanis**	**Tanis**	**Tanis**		
**Horizon**	P2 overlying Tonst	Tonstein	Ev-2 under Tonst	Ev-1 under Ev-Dep		
**Iridium ppb**	<0.02	3.87	<0.02	<0.02		

Table S6. Representative terrestrial and marine palynology.

Pal-ovr-tonstein = Paleogene strata over lignitic horizon; Event-Dep = Event-Deposit; Pt-bar = Point-bar; Incsd bdrk = Incised bedrock beneath point-bar.

Terrestrial Palynology				
**Terrestrial palynology: Tanis**	Pal-ovr-tonst	Event-Dep	Pt-bar	Incsd bdrk
**Fern spores (%)**				
**Trilete**	41.6	2.3	2.3	23.9
**Monolete**	14.6	2.3	3.1	2.8
**Pollen(%)**				
Taxodiaceapollenites	0.7	2.3	/	2.8
Pinuspollenites	21.9	2.3	1.6	19.7
Aquilapollenites	/	69.2	66.9	19.7
Wodehouseia spinata	0.7	10	16.5	4.4
Cranwellia striata	2.1	1.5	/	/
Tricolpites microreticulata	/	/	0.8	1.4
***Tschudypollis* sp.**	/	/	/	1.4
**Triporate**	13.1	3.1	4.7	14.1
**Tricolpate**	0.7	1.5	0.8	/
**Stephanoporate- 4 pores**	2.2	2.3	1.7	1.4
**Stephanoporate- 5 pores**	/	0.8	0.8	/
***Cheno-am* type**	/	/	/	5.6
**Unknown**	2.4	2.4	0.8	2.8

Marine Palynology		
**Designation**	**Taxon**	**Temporal range**
1	***Hystrichosphaeridiium tubiferum***	**Aptian-Eocene**[40, 42]
2	***Spiniferites ramosus***	**Santonian-?Neogene**[44, 45]
3	***Hafniasphaera septata***	**Latest Maastrichtian-Paleocene**[38]
4	***Pierceites pentagonus***	**Latest Maastrichtian-Paleocene**[38, 39]
5	***Cordosphaeridium fibrospinosum***	**Late Cetaceous-Paleogene**[39]
6	***Areoligera senoniensis***	**Late Campanian-Paleogene**[39]
7	***Achomosphaera ramulifera***	**Late Cretaceous-Paleogene**[44]
8	***Coronifera striolata***	**Santonian-?Paleogene**[39, 44]
9	***Oligosphaeridium pulcherrimum***	**Barremian-Paleocene**[40, 45]
10	***Triblastula utinensis***	**Early-Late Maastrichtian**[39, 44]
11	***Isabelidinium cooksoniae***	**Campanian-Late Maastrichtian**[39, 46]
12	***Coronifera oceanica***	**Santonian- ?Late Maastrichtian**[39, 44]
13	***Cribroperidinium wilsonii***	**Santonian-Late Campanian**[38, 44]
14	***Cyclonephelium compactum***	**Albian-Santonian**
15	***Rottnestia wetzelii***	**Campanian-Late Maastrichtian**
16	***Alterbidinium acutulum***	**Campanian-Early Maastrichtian**[57, 46]
17	***Palaeohystrichophora infusorioides***	**Cenomanian-Early Maastrichtian**[45]
18	***Odontochitina operculata***	**Barremian-Early Maastrichtian**[38, 45, 46]
19	***Trichodinium castanae***	**Valanginian-Campanian**[45]

Table S7. Fundamental criteria for identifying tsunamite, and applicability to Tanis.

Criteria diagnostic for tsunamite, which can be equally effective for seiche (see text). Data from Peters and Jaffe (***11***) and Morton et al (***13***).

Diagnostic criteria for tsunamite, and applicability to Tanis		
(Sensu Peters & Jaffe, 2010 [11])		Applicability to Tanis
Sharp or erosional basal contact		Yes
Great thickness (usually between ~20 and 150 cm thick)		Yes; thicker than typical storm or river-flood deposits
Few depositional subdivisions (~1 to 7)		Yes; few lithological subdivisions
Normal grading		Yes
Abundant basal rip-up clasts		Yes
Contribution from coastal sediments and/or bioclasts		Yes; marine bioclasts present
Capped with mud, fines, vegetation		Yes
Variable bimodal grainsize distribution		Yes; portions are bimodal
Atypical allochthonous marine organic debris		Yes; allochthonous macro- and microscopic marine organisms
Bedforms indicating rapid sedimentation		Yes; steady succession from high to low flow regime
Truncated flame structures		Yes
Bidirectional flow indicators		Yes
(Sensu Morton et al., 2007 [13])		
Evidence for short-lived event		Yes
Contextually atypical sediment package		Yes; starkly contrasts with the upper and lower bounding strata
Run-up distance exceeds that of storms		Yes; minimum runup of ~9 meters
Predominantly deposited out of suspension		Yes; Climbing ripples, succession from upper to lower flow regime
Drape of even thickness over macrotopography		Yes; broad, even drape over the steeply angled and uneven paleosurface
Shells scattered, not present in discrete laminae		Yes; marine mollusk shells are scattered and not in discrete laminae
Rafts of organic material		Yes; densest among the carcasses and capping the deposit
Few subdivided layers		Yes; few lithological subdivisions
Normally graded sequence		Yes

References

1. Fastovsky D (1987) Paleoenvironments of vertebrate-bearing strata during the Cretaceous-Paleogene transition, eastern Montana and western North Dakota. *Palaios* 2: 282-295.

2. Hartman J, Murphy R, Kirkland J (2002) Brackish and marine mollusks of the Hell Creek Formation of North Dakota: evidence for a persisting Cretaceous seaway. *The Hell Creek Formation and the Cretaceous-Tertiary Boundary in the Northern Great Plains: an integrated continental record of the end of the Cretaceous*. (Geological Society of America Special Paper 361) pp. 271-296.

3. Grande L, Bemis W (1991) Osteology and phylogenetic relationships of fossil and recent paddlefishes (Polyodontidae) with comments on the interrelationships of Acipenseriformes. *Journal of Vertebrate Paleontology* 11(s1): 1-121.

4. High Jr L, Picard M (1974) Reliability of cross-stratification types as paleocurrent indicators in fluvial rocks. *Journal of Sedimentary Petrology* 44: 158-168.

5. Winker C, Kidwell S (1986) Paleocurrent evidence for lateral displacement of the Pliocene Colorado River delta by the San Andreas fault system, southeastern California. *Geology* 1(4): 788-791.

6. Rigby Jr J et al. (1987) Dinosaurs from the Paleocene part of the Hell Creek Formation, McCone County, Montana. *Palaios* 2: 296-302.

7. Pearson D, Schaefer T, Johnson K, Nichols D, Hunter J (2002) Vertebrate biostratigraphy of the Hell Creek Formation in southwestern North Dakota and northwestern South Dakota. *The Hell Creek Formation and the Cretaceous-Tertiary Boundary in the Northern Great Plains: an integrated continental record of the end of the Cretaceous*. (Geological Society of America Special Paper 361) pp. 145-168.

8. Jobe Z, Lowe D, Morris W (2012) Climbing-ripple successions in turbidite systems: depositional environments, sedimentation rates and accumulation times. *Sedimentology* 59(3): 867-898.

9. Matsumoto D et al. (2008) Truncated flame structures within a deposit of the Indian Ocean tsunami: evidence of syn-sedimentary deformation. *Sedimentology* 55: 1559-1570.

10. Srinivasalu S et al. (2009) Characteristics of 2004 tsunami deposits of the northern Tamil Nadu coast, southeastern India. *Boletin de la Sociedad Geologica Mexicana* 61: 111-118.

11. Peters R, Jaffe B (2010) Identification of tsunami deposits in the geologic record: developing criteria using recent tsunami deposits (U.S. Geological Survey Open-File Report 2010-1239).

12. Nanayama F, Shigeno K, Shitaoka Y, Furukawa R (2011) Geological study of unusual tsunami deposits in the Kuril Subduction Zone for mitigation of tsunami disasters. *The tsunami threat- research and technology* (InTech, Rijeka, Croatia), pp. 283-298.

13. Morton R, Gelfenbaum G, Jaffe B (2007) Physical criteria for distinguishing sandy tsunami and storm deposits using modern examples. *Sedimentary Geology* 200: 184-207.

14. Allen J (1970) A quantitative model of climbing ripples and their cross-laminated deposits. *Sedimentology* 14: 5-26.

15. Baas J, Van Dam R, Storms J (2000) Duration of deposition from decelerating high-density turbidity currents. *Sedimentary Geology* 136: 71–88.

16. Bercovici A, Vajda V, Pearson D, Villanueva-Amadoz U, Kline D (2012) Palynostratigraphy of John's Nose, a new Cretaceous-Paleogene boundary section in southwestern North Dakota, USA. *Palynology* 36(1): 36-47.

17. Belza J et al. (2015) High spatial resolution geochemistry and textural characteristics of 'microtektite' glass spherules in proximal Cretaceous–Paleogene sections: Insights into glass alteration patterns and precursor melt lithologies. *Geochimica et Cosmochimica Acta* 152: 1-38.

18. Kring D, and Boynton W (1991) Altered spherules of impact melt associated relic glass from the K/T boundary sediments in Haiti. *Geochimica et Cosmochimica* Acta 55(6): 1737-1742.

19. Schulte P, Kotny A (2005). Chicxulub impact ejecta from the Cretaceous-Paleogene (K-P) boundary in northeastern Mexico. *GSA Special Paper on Large Meteorite Impacts* 384: 191-221.

20. Schulte P et al. (2003) Fe-rich and K-rich mafic spherules from slumped and channelized Chicxulub ejecta deposits in the northern La Sierrita area, NE Mexico. *International Journal of Earth Sciences* 92: 114-142.

21. Schulte P, Speijer R, Mai H, Kontny A (2006) The Cretaceous-Paleogene (K-P) boundary at Brazos, Texas: sequence stratigraphy, depositional events and the Chicxulub impact. *Sedimentary Geology* 184: 77-109.

22. Belza J et al. (2015) High spatial resolution geochemistry and textural characteristics of 'microtektite' glass spherules in proximal Cretaceous–Paleogene sections: Insights into glass alteration patterns and precursor melt lithologies. *Geochimica et Cosmochimica Acta* 152: 1-38.

23. Schulte P, Kotny A (2005) Chicxulub impact ejecta from the Cretaceous-Paleogene (K-P) boundary in northeastern Mexico. *GSA Special Paper on Large Meteorite Impacts.* 384: 191-221.

24. Bermudez H et al. (2015) The Cretaceous-Paleogene boundary at Gorgonilla Island, Colombia, South America. *Terra Nova* 28: 83-90.16

25. Premo W, Izett G, Meeker G (1995) Major-element and isotopic compositions of relict tektites and glass-like shards from the K-T boundary spherule bed at El Mimbral, Mexico. *Abstracts of the Lunar and Planetary Science Conference* 26: 1139.

26. Swisher C et al. (1992) Coeval 40Ar/39Ar ages of 65.0 million years ago from Chicxulub crater melt rock and Cretaceous-Tertiary boundary tektites. *Science* 257: 954-958.
32. Kuiper K et al. (2008) Synchronizing the rock clocks of Earth history. *Science* 320(5875): 500–504.

27. Kuiper K et al. (2008) Synchronizing the rock clocks of Earth history. *Science* 320(5875): 500–504.

28. Renne P et al. (2013) Time scales of critical events around the Cretaceous-Paleogene boundary. *Science* 339(6120): 684-687 doi:10.1126/science.1230492.

29. Renne P, Balco G, Ludwig K, Mundil R, Min K (2011) Response to the comment by W.H. Schwarz et al. on "Joint determination of ^{40}K decay constants and ^{40}Ar*/^{40}K for the Fish Canyon sanidine standard, and improved accuracy for ^{40}Ar/^{39}Ar geochronology by Renne et al. (2010). *Geochim. Cosmochim. Acta* 75(17): 5097–5100.

30. Bercovici A, Vajda V, Sweet A (2012) Pollen and spore stratigraphy of the Cretaceous-Paleogene mass-extinction interval in the Northern Hemisphere. *Journal of Stratigraphy* 36(2): 166-178.

31. Johnson K (2002) Megaflora of the Hell Creek and lower Fort Union Formations in the western Dakotas" Vegetational response to climate change, the Cretaceous-Tertiary boundary event, and rapid marine transgression. *The Hell Creek Formation and the Cretaceous-Tertiary Boundary in the Northern Great Plains: an integrated continental record of the end of the Cretaceous*. (Geological Society of America Special Paper 361) pp. 329-392.

32. Nichols D (2002) Palynology and palynostatigraphy of the Hell Creek Formation in North Dakota: a microfossil record of plants at the end of Cretaceous time. *The Hell Creek Formation and the Cretaceous-Tertiary Boundary in the Northern Great Plains: an integrated continental record of the end of the Cretaceous*. (Geological Society of America Special Paper 361) pp. 393-456.

33. Witts J et al. (2018) A fossiliferous spherule-rich bed at the Cretaceous-Paleogene (K-Pg) boundary in Mississippi, USA: Implications for the K-Pg mass extinction event in the Mississippi Embayment and Eastern Gulf Coastal Plain. *Cretaceous Research* 91: 147-167.

34. Dawson A (2000) Tsunami deposits. *Pure and Applied Geophysics* **157**: 875-897.

35. Maurrasse F, Lamolda M, Aguado R, Peryt D, Sen G (2004) Spatial and Temporal variations of the Haitian K/T Boundary record: implications concerning the event or events. *Journal of Iberian Geology* 31: 113-133.

36. Sawai Y et al. (2009) Diatom assemblages in tsunami deppsits associated with the 2004 Indian Ocean tsuami at Phra Thong Island, Thailand. *Marine Micropaleontology* 73: 70.

37. Yawsangratt S et al. (2012) Evidence of probable paleotsunami deposits on Kho Khao Island, Phang Nga Province, Thailand. *Natural Hazards* 63(1): 151-163.

38. Slimani H, Louwye S, Dusar M, Lagrou D (2011) Connecting the Chalk Group of the Campine Basin to the dinoflagellate cyst biostratigraphy of the Campanian to Danian in

borehole Meer (northern Belgium). *Netherlands Journal of Geosciences – Geologie en Mijnbouw* 90(2): 129-164.

39. Schiøler P, Wilson G (1993) Maastrichtian dinoflagellate zonation in the Dan Field, Danish North Sea. *Review of Palaeobotany and Palynology* 78: 321-351.

40. Verdier J (1975) Les kystes de dinoflagellés de la section de Wissant et leur distribution stratigraphique au Crétacé moyen. *Rév. Micropaléontol* 17(4): 191–197.

41. Duxbury S (1977) A palynostratigraphy of the Berriasian to Barremian of the Speeton clay of Speeton, England. *Palaeontographica B* 160: 17-67.

42. Mudge D, Bujak J (1996) An integrated stratigraphy for the Paleocene and Eocene of the North Sea. *Geological Society, London, Special Publications* 101: 91-113.

43. Schiøler P, Brinkhuis H, Roncaglia L, Wilson G (1997) Dinoflagellate biostratigraphy and sequence stratigraphy of the Type Maastrichtian (Upper Cretaceous), ENCI Quarry, The Netherlands. *Marine Micropaleontology* 31: 65-95.

44. Prince I, Jarvis I, Tocher B (1999) High-resolution dinoflagellate cyst biostratigraphy of the Santonian-basal Campanian (Upper Cretaceous): new data from Whitecliff, Isle of Wight, England. *Review of Palaeobotany and Palynology* 105**:** 143-169.

45. Williams G, Brinkhuis H, Pearce M, Fensome R, Weegink J (2004) Southern Ocean and global dinoflagellate cyst events compared: index events for the Late Cretaceous-Neogene. *Proc. ODP, Sci. Results* 189: 1-98.

46. Radmacher W, Pérez-Rodríguez I, Arz J, Pearce M (2014) Dinoflagellate biostratigraphy at the Campanian-Maastrichtian boundary in Zumaia, northern Spain. *Cretaceous Research* 51: 309-320.

47. Radmacher W, Mangerud G, Tyszka J (2015) Dinoflagellate cyst biostratigraphy of Upper Cretaceous strata from two wells in the Norwegian Sea. *Review of Palaeobotany and Palynology* 216: 18-32.

48. Kennedy W, Landman N, Christensen W, Cobban W, Hancock J (1998) Marine connections in North America during the late Maastrichtian: palaeogeographic and palaeobiogeographic significance of *Jeletzkytes nebrascensis* Zone cephalopod fauna from the Elk Butte Member of the Pierre Shale, SE South Dakota and NE Nebraska. *Cretaceous Research* 19: 745-775.

49. Kidwell S, Bosence D (1991) Taphonomy and time-averaging of marine shelly faunas. *Taphonomy: Releasing the Data Locked in the Fossil Record*. (Plenum, New York), pp. 115-209.

50. Landman N, Johnson R, Edwards L (2004) Cephalopods from the Cretaceous/Tertiary Boundary Interval on the Atlantic Coastal Plain, with a Description of the Highest Ammonite Zones in North America. Part 2. Northeastern Monmouth County, New Jersey. *Bulletin of the American Museum of Natural History* 287: 1-107.

51. Hoganson J, Murphy E (2002) Marine Breien Member (Maastrichtian) of the Hell Creek Formation in North Dakota: stratigraphy, vertebrate fossil record, and age. *The Hell Creek Formation and the Cretaceous-Tertiary Boundary in the Northern Great Plains: an integrated continental record of the end of the Cretaceous*. (Geological Society of America Special Paper 361) pp. 247-269.

52. Jeletzky J (1965) Taxonomy and phylogeny of fossil coleoida (=Dibranchiata). *Geological Survey of Canada, paper 65:*42: 71-76.

53. Wroblewski A (2004) New Selachian paleofaunas from "fluvial" deposits of the Ferris and lower Hanna formations (Maastrichtian-Selandian: 66-58 Ma), southern Wyoming. *Palaios* 19: 249-258.

54. Landman N, Remin Z, Garb M, Chamberlain Jr. J (2013) Cephalopods from the Badlands National Park area, South Dakota: Reassessment of the position of the Cretaceous/Paleogene boundary. *Cretaceous Research* 42.

55. Spicer R (1991) Plant taphonomic processes. *Taphonomy: Releasing the Data Locked in the Fossil Record* (Plenum, New York), pp. 71-113.

56. Behrensmeyer A (1991) Terrestrial vertebrate accumulations. *Taphonomy: Releasing the Data Locked in the Fossil Record*. (Plenum, New York), pp. 291-335.

57. Wilson M (1996) Taphonomy of a mass-death layer of fishes in the Paleocene Paskapoo Formation at Joffre Bridge, Alberta, Canada. *Canadian Journal of Earth Sciences* 33: 1487-1498.

58. Allison P, Briggs D (1991) Taphonomy of non-mineralized tissues. *Taphonomy: Releasing the Data Locked in the Fossil Record* (Plenum, New York), pp. 25-70.

59. Grande L (1984) Paleontology of the Green River Formation, with a review of

60. Elde R, Smith G (1988) Fish taphonomy and environmental inference in paleolimnology. *Palaeogeography, Palaeoclimatology, Palaeoecology* 62: 577-592.

61. Bienkowska M (2004) Taphonomy of ichthyofauna from an Oligocene sequence (Tylawa Limestones horizon) of the Outer Carpathians, Poland. *Geological Quarterly* 48(2): 181-192.

62. Barton D, Wilson M (2005) Taphonomic variations in Eocene fish-bearing varves at Horsefly, British Columbia, reveal 10 000 years of environmental change. *Canadian Journal of Earth Sciences* 42: 137-149.

63. Friedman M, Beckett H, Close R, Johanson Z (2015) The English Chalk and London Clay: two remarkable British bony fish Lagerstätten. *Arthur Smith Woodward: His Life and Influence on Modern Vertebrate Palaeontology*. (Geological Society, London, Special Publications), 430.

64. Anderson K, Woods A (2013) Taphonomy of Early Triassic fish fossils of the Vega-Phroso Siltstone Member of the Sulphur Mountain Formation near Wapiti Lake, British Columbia, Canada. *Journal of Palaeogeography* 2: 321-343.

65. Erickson G, Currie P, Inouye B ,Winn A (2010) A revised life table and survivorship curve for *Albertosaurus sarcophagus* based on the Dry Island mass death assemblage. *Canadian Journal of Earth Sciences* 46: 1269-1275.

66. Maurrasse F, Sen G (1991) Impacts, tsunamis, and the Haitian Cretaceous-Tertiary boundary layer. *Science* 252: 1690-1693.

67. Smit J et al. (1992) Tektite-bearing deep-water clastic unit at the Cretaceous-Tertiary boundary in northeastern Mexico. *Geology* 20: 99-103.

68. Archer A (1991) Modeling of tidal rhythmites using modern tidal periodicities and implications for short-term sedimentation rates (Kansas Geological Survey, Bulletin 233), pp. 185-194.

69. Kortekaas S (2002) Tsunamis, storms, and earthquakes: Distinguishing coastal flooding events. Dissertation. Coventry University.

70. Morton R, Paine J (1985) Beach and vegetation-line changes at Galveston Island, Texas. Erosion, deposition, and recovery from Hurricane Alicia (The University of Texas at Austin, Bureau of Economic Geology Geological Circular 85-5).

71. Needham H, and Keim B (2012) A storm surge database for the US Gulf Coast. International Journal of Climatology 32: 2108-2123.

72. Hassler S, Robey H, Simonson B (2000) Bedforms produced by impact-generated tsunami, 2.6 Ga Hamersley basin, Western Australia. *Sedimentary Geology* 135: 283-294.

73. Matsui T, Imamura F, Tajika E; Nakano Y, Fujisawa Y (2002) Generation and propagation of a tsunami from the Cretaceous-Tertiary impact event. *Catastrophic Events and Mass Extinctions: Impacts and Beyond* (Geological Society of America Special Paper 356, Boulder, Colorado), pp. 69–77.

74. Halif M, Sabki S (2005) The Physics of Tsunami: Basic understanding of the Indian Ocean. *American Journal of Applied Sciences* 2(8): 1188-1193.
